# Isolation of an Anionic Dicarbene Embedded Sn_2_P_2_ Cluster and Reversible CO_2_ Uptake

**DOI:** 10.1002/advs.202305545

**Published:** 2023-11-28

**Authors:** Falk Ebeler, Yury V. Vishnevskiy, Beate Neumann, Hans‐Georg Stammler, Rajendra S. Ghadwal

**Affiliations:** ^1^ Molecular Inorganic Chemistry and Catalysis Inorganic and Structural Chemistry Center for Molecular Materials Faculty of Chemistry Universität Bielefeld Universitätsstrasse 25 33615 Bielefeld Germany

**Keywords:** anionic dicarbene, carbon dioxide, cluster, main‐group, tin phosphorus

## Abstract

Decarbonylation of a cyclic bis‐phosphaethynolatostannylene [(ADC)Sn(PCO)]_2_ based on an anionic dicarbene framework (ADC = PhC{N(Dipp)C}_2_; Dipp = 2,6‐*i*Pr_2_C_6_H_3_) under UV light results in the formation of a Sn_2_P_2_ cluster compound [(ADC)SnP]_2_ as a green crystalline solid. The electronic structure of [(ADC)SnP]_2_ is analyzed by quantum‐chemical calculations. At room temperature, [(ADC)SnP]_2_ reversibly binds with CO_2_ and forms [(ADC)_2_{SnOC(O)P}SnP]. [(ADC)SnP]_2_ enables catalytic hydroboration of CO_2_ and reacts with elemental selenium and Fe_2_(CO)_9_ to afford [(ADC)_2_{Sn(Se)P_2_}SnSe] and [(ADC)Sn{Fe(CO)_4_}P]_2_, respectively. All compounds are characterized by multinuclear NMR spectroscopy and their solid‐state molecular structures are determined by single‐crystal X‐ray diffraction.

## Graphical abstract

The Sn_2_P_2_ cluster compound [(ADC)SnP]_2_ based on an anionic dicarbene (ADC) is isolated as a green crystalline solid. [(ADC)SnP]_2_ reversibly binds with CO_2_ at room temperature to form [(ADC)_2_{SnOC(O)P}SnP] and enables catalytic hydroboration of CO_2_.

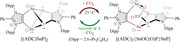

## 1 Introduction

**Scheme 1:**
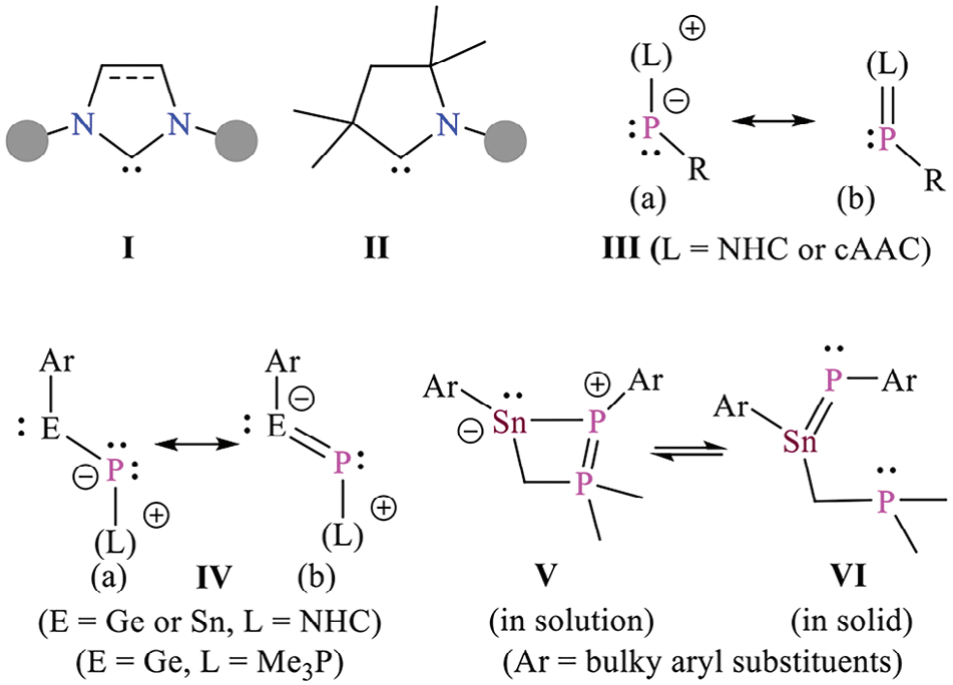
Schematic illustration of NHC (**I**), cAAC (**II**), and phosphinidene adducts (**III**). Selected examples of metallylene‐phosphinidene compounds (**IV**‐**VI**).

Stable compounds with multiple bonds between heavier main‐group elements continue to attract interest owing to their intriguing electronic structures, reactivity profiles, and potential in synthesis.^[^ ^1^ ^]^ The isolation of the first alkene analogs of phosphorus (i.e., diphosphene) by Yoshifuji^[^ ^2^ ^]^ and silicon (i.e., disilene) by West^[^ ^3^ ^]^ in 1981 was an important milestone. The advent of stable carbenes, such as N‐heterocyclic carbenes (NHCs, **I**)^[^ ^4^ ^]^ and cyclic alkyl amino carbenes (cAACs, **II**),^[^ ^5^ ^]^ furnished new tools to tame the reactive, including unsaturated, main‐group species (**Scheme** **1**).^[^ ^6^ ^]^ Early synthesis of the first NHC‐stabilized phosphinidene **III** (R ≐ Ph),^[^ ^7^ ^]^ monomer of a diphosphene, emphasized the potential of NHCs as potent Lewis bases.^[^ ^8^ ^]^ Depending on the π‐acceptor property of carbene, **III** may be regarded as base‐stabilized phosphinidenes (a) or phosphaalkenes (b).

Stable phosphaalkynes RC≡P,^[^ ^9^ ^]^ the heavier analogs of nitriles (RC≡N), have been known since 1981.^[^ ^10^ ^]^ The related heavier Group 14 compounds RE≡P (E = Si, Ge, Sn, Pb) remained however rather elusive species because of their low thermodynamic stability and high propensity to oligomerize. This is generally attributed to the reluctance of heavier main‐group elements to form multiple covalent bonds.^[^ ^1^ ^]^ Theoretical calculations predicted that the vinylidene isomers RP═E are thermodynamically more stable than those of alkyne analogs RE≡P.^[^ ^11^ ^]^ Recently, Inoue^[^ ^12^ ^]^ and Tan^[^ ^13^ ^]^ reported the syntheses of Lewis base‐stabilized germanium and tin compounds **IV** (Scheme 1) with a partial E–P double bond. A base‐stabilized metallylene‐phosphinidene description (a) appears more appropriate for **IV** as shown by the isolation of its Sn‐bound B(C_6_F_5_)_3_ adduct.^[^ ^12^ ^]^ In 2022, Aldridge and colleagues described the synthesis of phosphino‐phosphinidene tethered stannylenes **V**.^[^ ^14^ ^]^ In solutions, **V** exist as P‐donor stabilized stannylenes featuring a four‐membered SnCP_2_ heterocycle. Using sterically very bulky substituents, Aldridge et al. were able to characterize a stannaphosphene **VI**, featuring a polar Sn═P bond, in the solid state by X‐ray diffraction.

**Scheme 2:**
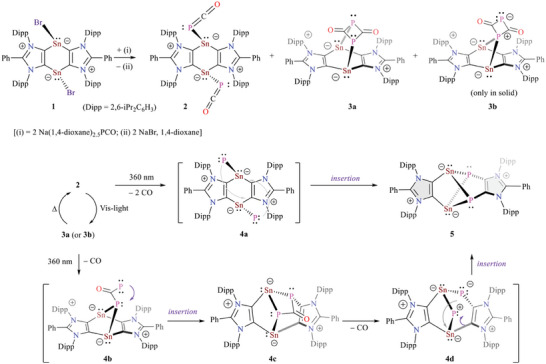
Synthesis of **2**, **3**, and **5**.

**Scheme 3:**
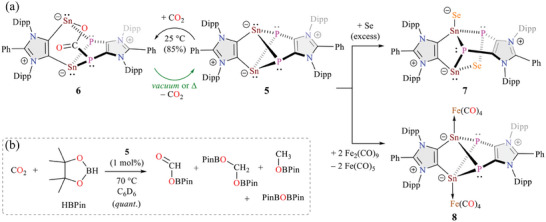
a) Reactions of **5** with CO_2_, selenium, and Fe_2_(CO)_9_ to **6**, **7**, and **8**, respectively. b) Catalytic hydroboration of CO_2_ with **5**.

In the absence of an external Lewis base (L), the putative phosphinide species (RE–P), generated usually by the decarbonylation of the corresponding phosphaethynolato‐compounds (RE–PCO),^[^ ^15^ ^]^ undergoes head‐to‐head or head‐to‐tail dimerization to give diphosphene (RE–P=P–ER) or cyclobutadiene (with a E_2_P_2_ ring) derivatives.^[^ ^15^, ^16^ ^]^ Herein, we report the isolation of an unprecedented Sn_2_P_2_ cluster compound [(ADC)SnP]_2_ (**5**) as green crystals by the decarbonylation of a cyclic bis‐phosphaethynolatostannylene [(ADC)Sn(PCO)]_2_ (**2**) (ADC = PhC{N(Dipp)C}_2_; Dipp = 2,6‐*i*Pr_2_C_6_H_3_) as well as the exploration of its structure and reactivity (**Schemes** **2** and **3**).

## 2 Results and Discussion

Treatment of [(ADC)SnBr]_2_ (**1**)^[^ ^17^ ^]^ with Na(1,4‐dioxane)_2.5_PCO afforded a mixture of [(ADC)Sn(PCO)]_2_ (**2**) and [(ADC)Sn{P(µ‐CO)]_2_ (**3a**) in 3:2 ratio as a yellow solid (Scheme 2). Heating a benzene suspension of **2** and **3a** at 70 °C for 1 h led to the conversion of **3a** into **2**, which precipitated out in benzene at room temperature. **2** was isolated by filtration in 84% yield as a bright yellow solid. The soluble part contained a mixture of **2** and **3a**, which could not be separated because of their comparable solubility (see below). Compound **2** is stable under an inert gas atmosphere but slowly isomerizes in daylight to give the OC‐bridged species **3a**. Therefore, a pure sample of **3a** (free from **2**) could not be obtained because of the incomplete conversion of **2** into **3a** and their almost similar solubility. Thus, NMR data of **3a** were extracted from the spectra measured for a sample containing **2** and **3a**. In addition to the expected ^1^H and ^13^C NMR signals for the ADC moiety, the ^31^P{^1^H} (−361 ppm) and ^119^Sn{^1^H} (−165.5 ppm, ^1^ *J*_P‐Sn_ = 637 Hz) NMR spectra of **2** show a singlet and a doublet, respectively. The ^119^Sn{^1^H} NMR signal for **2** compares well with those of Lewis base stabilized stannylenes with a three‐coordinated Sn atom.^[^ ^18^ ^]^ The IR spectrum of **2** shows a strong band for the unsymmetric stretching frequency of the phosphaketene unit at ν_asym_ = 1860 cm^−1^.^[^ ^15a^ ^]^ The ^31^P{^1^H} (singlet at 170.1 ppm) and ^119^Sn{^1^H} (doublet at –148.1 ppm with ^1^ *J*_P‐Sn_ = 668 Hz) NMR signals for **3a** are consistent with those of OC‐bridged OCP‐containing compounds.^[^ ^19^ ^]^

**Figure 1:**
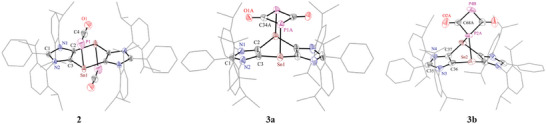
Solid‐state molecular structures of **2**, **3a**, and **3b**. Aryl groups are shown as wireframe models. H atoms, minor occupied disordered atoms, and solvent molecules are omitted for clarity. Thermal ellipsoids are shown with 50% probability. Selected bond lengths (Å) and angles (°) for **2**: C2–C3 1.377(7), C3–Sn1 2.198(5), Sn1–P1 2.677(2), P1–C4 1.503(8), C4–O1 1.280(9), and C2–Sn1–P1 95.1(1), C4–P1–Sn1 92.2(3), C2–C3–Sn1 133.1(4), O1–C4–P1 176.1(6); for **3a**: C2–C3 1.359(8), C2–Sn1 2.225(6), Sn1–P1A 2.711(2), P1A–C34A 1.862(9), C34A–O1A 1.249(11), and C2–Sn1–P1A 94.5(2), C34A–P1A–Sn1 100.2(3), C2–C3–Sn1 129.6(4), O1A–C34A–P1A 130.4(7), C34A–P1A–C34A 80.2(4), P1A–C34A–P1A1 99.8(4); for **3b**: C36–C37 1.361(8), C36–Sn2 2.240(7), Sn2–P2A 2.697(2), P2A–C68A 1.885(9), P4B–C68A 1.807(9), C68A–O2A 1.212(9), C36–Sn2–P2A 77.1(2), C68A–P2A–Sn2 120.3(2), C36–C37–Sn2 125.8(5), O2A–C68A–P2A 128.3(7), P4B–C68A–P2A 98.1(4), C68A–P4B–C68A 84.0(5).

Suitable crystals for single crystal X‐ray diffraction (sc‐XRD) were grown by storing a *n*‐hexane layered concentrated THF solution of **2** at −40 °C. Two nearly identical molecules of **2** were found inside the asymmetric unit. Both tin atoms of the central almost planar C_4_Sn_2_ ring of **2** (**Figure** **1**) show threefold coordination. The linear PCO substituents at the tin atoms are present in a *trans* fashion and positioned inwards the C_4_Sn_2_ ring. Single crystals of **3a** were obtained by storing a benzene solution of a mixture of **2** and **3a** at 5 °C and picked up by visual inspection. The crystals of **3a** also contained the isomer **3b**, both sharing the same positions on two different twofold axes of the space group I2. The asymmetric unit thus contains two half‐disordered molecules of **3a** and **3b** in the ratio 70:30 and 17:83, respectively (see Scheme 2). In solution NMR studies, **3b** could not be detected. Thus, the formation of **3b**, featuring one four‐coordinated and one two‐coordinated phosphorus atoms of a dimeric phosphaethynolate species, is likely due to crystal packing effects.^[^ ^19^ ^]^ The Sn–C bond lengths in **2**, **3a**, and **3b** (Figure 1) range from 2.198(5) to 2.240(7) Å, which are comparable with related tin compounds based on ADCs.^[^ ^18a,b^ ^]^ The Sn–P (2.677(2) Å) and P–C (1.503(8) Å) bond lengths of **2** are consistent with a terminal phosphaketene unit P═C═O.^[^ ^15d^ ^]^ As expected, the P–C bond lengths in **3a** (1.862(9) Å) and **3b** (1.885(9) Å) are longer than those of **2** but are similar to related OC‐bridged compounds.^[^ ^19^ ^]^

At elevated temperature (>80 °C), the solution of **2** begins to decarbonylate, leading to the formation of a new species, i.e., **5** (Scheme 2). The conversion is however rather slow (≈20% after 3 h). The irradiation of an orange fluorobenzene solution of **2** (or a mixture of **2** and **3a**) under UV light (360 nm) for 3 h cleanly affords compound [(ADC)SnP]_2_ (**5**) as the sole product (90% isolated yield). Compound **5** is a green solid and stable under an inert gas atmosphere but readily decomposes when exposed to air. Interestingly, **5** is remarkably thermal stable and no change, as monitored by NMR spectroscopy, was observed when a sample of **5** was heated at 150 °C for 3 h. **5** exhibits well‐resolved ^1^H and ^13^C{^1^H} NMR signals for the ADC moieties. The ^31^P{^1^H} NMR spectrum of **5** shows a singlet at −110 ppm with tin satellites. Albeit with a smaller ^1^ *J*_P‐Sn_ coupling constant (416 Hz), the ^31^P NMR signal of **5** (−110 ppm) is consistent with those of diphosphanylstannylene compounds (R_2_P)_2_Sn (−53 to −129 ppm; ^1^ *J*_P‐Sn_ = 1000–2000 Hz).^[^ ^20^ ^]^ The ^119^Sn{^1^H} spectrum of **5** exhibits a triplet at 748.1 ppm (^1^ *J*_P‐Sn_ = 416 Hz). These data indicate the presence of Sn–P single bonds and the coupling of tin atoms with two magnetically equivalent ^31^P nuclei. The remarkably downfield shifting of the ^119^Sn NMR signal and a rather smaller coupling constant for **5** (748.1 ppm, ^1^ *J*_P‐Sn_ = 416 Hz) with respect to that of **2** (−165.5 ppm, 643.5 Hz) and **3a** (−148.1 ppm, 663.7 Hz) is likely due to the Sn_2_P_2_‐ring strain and polar Sn–P bonds (see below).^[^ ^21^ ^]^ The ^119^Sn NMR signal of **5** is comparable with the chemical shifts reported for phosphastannirane (716 ppm)^[^ ^22^ ^]^ as well as homonuclear tin cluster compounds [Sn(2,6‐Mes_2_C_6_H_3_)]_4_ (773 ppm)^[^ ^23^ ^]^ and [Sn_8_(2,6‐Mes_2_C_6_H_3_)_4_] (750 ppm).^[^ ^24^ ^]^ Note, the values of ^31^P NMR chemical shifts (−91.4–−98.6 ppm) and ^1^ *J*_P‐Sn_ (539–656 Hz) for the three coordinated phosphorus atom of **V** (Scheme 1)^[^ ^14^ ^]^ are comparable to those of **5**. While the ^1^ *J*_P‐Sn_ (1648 Hz) for **IV** (E = Sn)^[^ ^12^ ^]^ with a partial Sn≐P bond is larger than that of **5**.

Thermal or photo‐induced decarbonylation of phosphaethynolato‐compounds (RE–PCO) is known to generate phosphinidene species (RE–P),^[^ ^15^ ^]^ which can also be isolated as monomeric compounds.^[^ ^25^ ^]^ The exact mechanism for the formation of **5** is currently unknown. The decarbonylation of **2** is likely to result in a putative bis‐phosphinidene species **4a** (Scheme 2), which spontaneously undergoes insertions into the Sn–C bonds to form **5**. Our preliminary calculations predict a triplet ground state for **4a** (see the Supporting Information), which is energetically 104 kcal mol^−1^ higher than **5**. The exposure of a C_6_D_6_ sample of **2** to white light enhances the conversion of **2** into **3a**. Therefore, an alternative mechanism in which **3a** (via **3b**) step‐wise undergoes decarbonylation and Sn–C bond insertion (via **4b → 4c → 4d**) to ultimately yield **5** cannot be ruled out. In situ NMR monitoring of a sample, however, indicates the presence of **2**, **3a**, and **5**, with no sign of any additional species such as **4c**.

**Figure 2:**
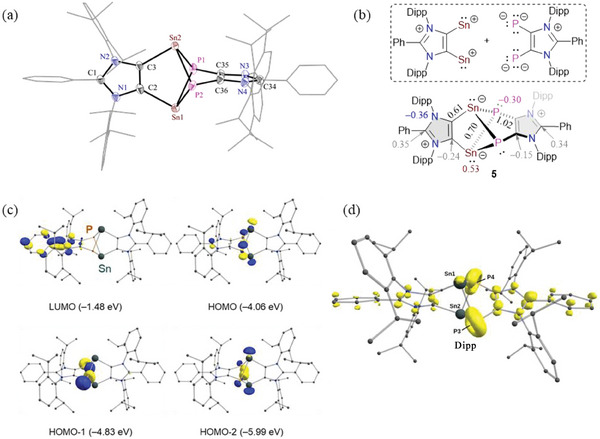
a) Solid‐state molecular structure of **5**. Aryl groups are shown as wireframe models. H atoms, minor occupied disordered atoms, and solvent molecules are omitted for clarity. Thermal ellipsoids are shown with 50% probability. Selected bond lengths (Å) and angles (°) for **5**: C2–C3 1.372(4), C35–C36 1.378(4), Sn1A–C2 2.218(3), Sn2A–C3 2.245(3), Sn1A–P1A 2.643(3), Sn1A–P2A 2.672(4), Sn2A–P1A 2.695(3), Sn2A–P2A 2.647(5), P1A–C35 1.822(4), P2A–C36 1.853(5), and C2–Sn1A–P2A 90.5(1), C2–Sn1A–P1A 88.6(1), Sn1A–P1A–C35 86.1(1), Sn1A–P1A–Sn2A 83.7(1), P1A–Sn1A–P2A 80.9(1), Sn1A–C2–C3 119.6(2), P1A–C35–C36 123.3(3). b) Selected NBO charges and WBIs for **5**. c) Selected frontier molecular orbitals (at PBE0/def2‐TZVPP) of **5**. d) FOD plot of **5**.

The solid‐state molecular structure of **5** (**Figure** **2**a) features a tricyclo[3,3,0,0] C_2_Sn_2_P_2_C_2_ core. The central twisted Sn_2_P_2_ ring shows a positional disorder by interchanging the connection of C2 and C3 to P (30%) or Sn (70%), respectively. The fold angle of this ring along the Sn–Sn axis is 59.7(1)° and 59.8(1)° respectively, and along the P–P axis is 59.5(1)° and 59.8(1)° respectively. The Sn_2_P_2_‐ring is capped by peripheral 1,3‐imidazole units comprising of a plane angle of 77.3°. Each of the phosphorus and tin atoms of **5** is three‐coordinated. The C–P (1.822(4)–1.853(5) Å), C–Sn (2.218(3)–2.245(3) Å), and Sn–P (2.639(3)–2.721(8) Å) bond lengths of **5** are essentially in line with single bond lengths of tin‐phosphorus compounds.^[^ ^26^ ^]^ The sum of the angles at the Sn (Σ = 260.01°) and P (Σ = 257.43°) atoms are consistent with the presence of a stereoactive lone‐pair.

To shed further light on the electronic structure **5**, we performed quantum chemical calculations (see the Supporting Information for details). The DFT optimized structure of **5** (Figure S60, Supporting Information) at the PBE0‐D3BJ/def2‐TZVPP level of theory is in good agreement with its X‐ray diffraction structure (Figure 2a).

Calculations suggest a closed‐shell singlet ground state for **5**, which is 26.9 kcal mol^−1^ lower than the triplet state. The reluctance of heavier main‐group elements to participate in π‐bonding interactions is evident in the frequent formation of clusters and cages.^[^ ^27^ ^]^ Thus, like the main‐group phosphanediides with bridging RP^2−^ ligands,^[^ ^28^ ^]^ **5** may be regarded as a Sn(II) phosphanediide species (see Figure 2b), in which Sn–P bonds are polarized toward phosphorus atoms. The values of NBO (Natural Bonding Orbital) charges and WBIs (Wiberg Bond Indices) for **5** (Figure 2b) are consistent with this description. The HOMO and HOMO–2 of **5** (Figure 2c) are the σ‐lone‐pairs at the tin atoms with a considerable contribution of phosphorus p‐orbitals. The LUMO is located mainly on the ligand, while the HOMO–1 resides largely at the phosphorus atoms. The UV–vis spectrum of **5** exhibits three absorption bands at *λ*_max_ = 306, 420, and 688 nm. Based on TD‐DFT calculations, they may be assigned to HOMO–2 → LUMO+2, HOMO–1 → LUMO+1, and HOMO → LUMO transitions, respectively (Table S7, Supporting Information).

We also performed fractional occupation number weighted density (FOD) calculations (at the PBE0/def2‐TZVPP level of theory) originally introduced by Grimme et al. as a static electron correlation (SEC) diagnostic.^[^ ^29^ ^]^ FOD analyses provide reliable information on the localization of “hot” (strongly correlated and chemically active) electrons in a molecule. The FOD plot (Figure 2d) and the resulting *N*_FOD_ number (3.07 *e*) indicate relatively large electron correlation in **5**. To further analyze the static electron correlation in **5**, we performed SS‐CASSCF (state‐specific complete active space self‐consistent field) calculations with a CAS(8,8) active space (see the Supporting Information for details). The singlet ground state solution at the CASSCF/def2‐TZVPP level with an occupation pattern of “22220000” (91%) and four other double excited configurations (1.1–1.5% each) point to a rather negligible diradical character^[^ ^30^ ^]^ of **5**.

Compounds **IV** (E = Sn, Scheme 1) with a partial Sn≐P double bond was found to be inert toward H_2_, CO, and CO_2_ but underwent the reaction with Ph_2_C═C≐O to form a [2+2]‐cycloaddition product with a four‐membered SnPCO ring.^[^ ^12^ ^]^ We prompted to reason that the ring strain and highly polarized nature of Sn–P bonds may be attributed to the interesting reactivity of **5**. We therefore decided to explore the reactivity of **5** with small molecules.^[^ ^31^ ^]^ No reaction between **5** and carbon monoxide (1 atm) to give **2** or **3a** was observed even at elevated temperatures. Similarly, no reaction between **5** and H_2_ (1 atm) was observed. The exposure of a green C_6_D_6_ solution of **5** to carbon dioxide (1 atm) at room temperature led to a wine‐red solution after 3 h. NMR studies revealed the presence of a mixture of **5** (15%) and **6** (85%) (Scheme 3a). Warming the reaction mixture led to the depletion of **6** and the restoration of **5**. In addition, removal of the volatiles under vacuum at room temperature resulted in the complete regeneration of **5**. These data collectively suggest reversible CO_2_ uptake with **5** (Scheme 3a). Such reversible reactions with main‐group compounds are of considerable significance for their potential in sustainable synthesis.^[^ ^1^, ^31^, ^32^ ^]^ In contrast, compound **5** irreversibly reacts with CS_2_, yielding a new species that is completely different than **6** according to NMR analyses. Further characterization of this species in currently underway in this laboratory. Reaction of **5** with CO_2_ to give **6** is calculated to be thermodynamically favored by 16.9 kcal mol^−1^, while the further reaction of **6** with CO_2_ to yield **6**‐CO_2_ is endothermic by 3.3 kcal mol^−1^ (see Figure S66, Supporting Information). Moreover, the comparison of the energy of HOMO of **5** (−4.06 eV) and **6** (−4.24 eV) suggests that the latter is less basic.

Encouraged by these findings, we sought to investigate the catalytic reduction of CO_2_ with **5**. Indeed, **5** enables catalytic hydroboration of CO_2_ with pinacolborane (HBpin) to form different products (Scheme 3b) depending on the reaction conditions. Catalytic hydroboration of CO_2_ (1 atm) was carried out with different loadings of **5** (1 or 5 mol% with respect to HBpin) and HBpin in C_6_D_6_ either at room temperature or at 70 °C. The conversion was monitored by ^1^H and ^11^B NMR spectroscopy (see the Supporting Information). At room temperature, full consumption of HBpin was achieved after 20 h with 5 mol% of **5**, giving rise to HCO_2_Bpin as the sole product. At 70 °C, the complete consumption of HBPin required 2 h with 1 mol% of **5** to result in a mixture of HCO_2_Bpin, CH_3_OBpin, CH_2_(OBpin)_2_, and O(Bpin)_2_. The use of 5 mol% of **5** at 70 °C led to the formation of HCO_2_Bpin as the main product only after 10 min. No reaction between CO_2_ and HBpin was observed under similar experimental conditions. These preliminary findings emphasize the potential of **5** as a main‐group catalyst in synthesis.^[^ ^31^, ^33^ ^]^

To obtain suitable single crystals of **6** for sc‐XRD, a freshly prepared equilibrium solution of **5** (15%) and **6** (85%) in toluene under CO_2_ atmosphere (1 atm) was stored at −35 °C for three days. The obtained red crystals were analyzed by sc‐XRD. The sc‐XRD data revealed that the resulting crystals contain **5** and **6** in 11:89% ratio (see the Supporting Information). Interesting, the drying of crystals under vacuum also led to the removal of CO_2_ to yield **5**. Thus, a pure sample of **6** for NMR studies could not be obtained. The NMR data for **6** were obtained by analyzing a sample containing **5** and **6** in 15:85 ratio and collected by subtracting the signals due to **5**. As expected, the formation of **6** from **5** leads to the lowering of symmetry. Thus, the ^1^H NMR spectrum of **6** exhibits a doublet and a septet for each of the isopropyl groups, which is consistent with the ^13^C{^1^H} NMR spectrum of **6**. The ^31^P{^1^H} NMR spectrum of **6** shows two distinct doublets at −171.2 and −74.1 ppm (^2^ *J*_P‐P_ = 25.4 Hz) accompanied by the corresponding tin satellites. The former is high‐field (Δ*δ* = 61 ppm) while the latter is downfield (Δ*δ* = 36 ppm) shifted with respect to that of **5** (−110.4 ppm). The ^119^Sn{^1^H} NMR spectrum of **5** reveals one pseudo triplet at 373.9 ppm (^1^ *J*_Sn‐P_ = 663.2 Hz for SnP_2_ unit) and a doublet at 466.6 ppm (^1^ *J*_Sn‐P_ = 666.2 Hz for OSnP moiety). This is consistent with the presence of two magnetically inequivalent phosphorus as well as tin nuclei in **6**.

**Figure 3:**
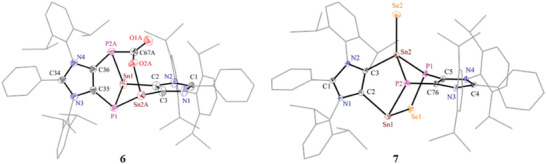
Solid‐state molecular structures of **6** and **7**. Aryl groups are shown as wireframe models and H atoms are omitted for clarity. Selected bond lengths (Å) and angles (°) for **6**: C2–C3 1.391(6), C2–Sn1 2.214(4), C3–Sn2A 2.231(5), Sn2A–O2A 2.137(4), O2A–C67A 1.297(7), O1A–C67A 1.217(7), P2A–C67A 1.853(6), P2A–C36 1.847(5), P1–C35 1.814(5), Sn1–P1 2.595(1), Sn1–P2A 2.672(1), Sn2A–P1 2.611(1), and C3–C2–Sn1 126.1(3), C2–C3–Sn2A 125.5(3), C3–Sn2A–P1 84.7(1), C35–P1–Sn1 89.3(2), C35–P1–Sn2A 97.7(1), P1–Sn1–P2A 87.9(1), C36–P2A–C67A 101.9(2), O1A–C67A–P2A 115.5(4), O1A–C67A–O2A 125.2(5), C67A–O2A–Sn2A 123.3(3); for **7**: C2–C3 1.370(5), C2–Sn1 2.243(3), C3–Sn2 2.158(3), Sn1–Se1 2.681(1), Sn1–P2 2.657(1), Sn2–Se2 2.415(1), Sn2–P1 2.618(1), Sn2–P2 2.551(1), Se1–P1 2.252(1), P1–C5 1.839(4), P2–C76 1.844(3), and C2–Sn1–P2 90.0(1), C2–Sn1–Se1 88.1(1), Sn1–P2–C76 101.3(1), Sn1–Se1–P1 106.4(1), Sn1–P2–Sn2 91.3(1), C3–Sn2–P1 100.9(1), C3–Sn2–P2 97.0(1), P1–C5–C76 128.5(3), C3–Sn2–Se2 118.5(1), P2–C76–C5 127.4(2).

As revealed by the solid‐state molecular structure of **6** (**Figure** **3**), one molecule of CO_2_ has been inserted into one Sn–P bond of **5**. The oxygen atom of the inserted CO_2_ unit binds to the tin (Sn–O, 2.137(4) Å) while the carbon atom is attached to the phosphor atom (P–C, 1.853(6) Å). This is in line with the polarity of Sn–P bonds of **5** (see Figure 2b). The Sn–O and P–C bond lengths of **6** compare well with the CO_2_ adduct of tin/phosphorus‐based frustrated Lewis pair (2.233(2); 1.891(3) reported by Mitzel et al., respectively).^[^ ^34^ ^]^ Other P–C bond lengths of **6** (1.814(5)–1.853(6) Å) correspond to single bonds (1.86 Å). The peripheral 1,3‐imidazole units are slightly less tilted (plane angle of 74.°) than in **5**. The Sn–C bonds (2.214(4) and 2.231(1) Å) are in line with those of **5** (2.218(3)–2.245(3) Å). The C–O bond of the carbonyl oxygen (1.217(7) Å) is slightly shorter than that attached to the tin atom (1.297(7) Å). Both C–O bonds are longer than the C–O double bond of gaseous CO_2_ (1.16 Å).^[^ ^35^ ^]^ The sum of the angles at the Sn (Σ = 273.20°, 271.77) and P (Σ = 287.48°, 287.95) atoms are consistent with the presence of a stereoactive lone‐pair.

Further reactivity studies of **5** were performed with elemental selenium and Fe_2_(CO)_9_ to afford compounds **7** and **8**, respectively (Scheme 3a). Compound **7** was isolated as a red solid in 88% yield, which is formally a mixed‐valent Sn(II)/Sn(IV) species. Insertion of one selenium atom into a Sn–P bond is akin to the formation of **6**. The second tin atom, like stannylenes,^[^ ^18b^ ^]^ reacts with selenium to result in a formal Sn(IV) center. Expectedly, the ^119^Sn{^1^H} NMR spectrum of **7** features two double doublets at 78.1 (^1^ *J*_Sn‐P_ = 804.2, 693.3 Hz) and −66.5 (^1^ *J*_Sn‐P_ = 809.7, 72.6 Hz) ppm, which may be assigned to Sn(IV) and Sn(II) nuclei, respectively. The ^31^P{^1^H} NMR spectrum of **7** shows two doublets at −110.1 and −121.5 (^2^ *J*_P‐P_ = 27 Hz) ppm along with tin satellites. The bis‐stannylene iron(0) complex **8** was isolated as a red‐brown solid in 50% yield. The ^119^Sn{^1^H} NMR spectrum of **8** shows a triplet at 494.9 ppm (^1^ *J*_Sn‐P_ = 493.8 Hz), which is high‐field shifted relative to that of **5** (748.1 ppm, ^1^ *J*_P‐Sn_ = 416 Hz).

**Figure 4:**
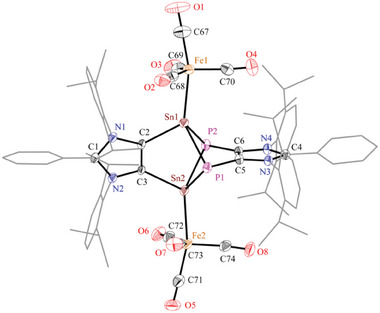
Solid‐state molecular structure of **8**. Aryl groups are shown as wireframe models and H atoms are omitted for clarity. Selected bond lengths (Å) and angles (°): C2–C3 1.367(4), C5–C6 1.378(4), Sn1–C2 2.197(3), Sn2–C3 2.206(2), Sn1–P1 2.586(1), Sn1–P2 2.630(1), Sn2–P1 2.648(1), Sn2–P2 2.584(1), P1–C5 1.823(3), P2–C6 1.825(3), Sn1–Fe1 2.505(1), Fe1–C68 1.784(3), C68–O2 1.153(4), and C2–Sn1–P2 96.3(1), C3–Sn2–P1 95.4(1), Sn1–P1–C5 86.5(1), Sn1–P1–Sn2 78.5(1), Sn1–C2–C3 115.7(2), P1–C5–C6 124.2(2), C2–Sn1–Fe1 122.6(1), P2–Sn1–Fe1 117.8(1).

The solid‐state molecular structures of **7** (Figure 3) and **8** (**Figure** **4**) show the expected atom connectivity and corroborate well with their spectroscopic data. The terminal Sn–Se bond length of **7** (2.415(1) Å) is in good agreement with that of a distannabarrelene derivative (2.388(5) Å) based on an ADC ligand^[^ ^18b^ ^]^ as well as of other tin compounds (2.375(3)−2.394(1) Å)^[^ ^36^ ^]^ featuring a terminal Sn–Se bond. The bridging Sn–Se bond length of **7** (2.681(1) Å) is slightly longer than the Sn─Se single bond lengths (2.55–2.60 Å),^[^ ^37^ ^]^ probably due to the ring strain. The P–Se bond length of **7** (2.252(1) Å) is in line with the P–Se single bond lengths in P_6_Se_6_ macrocycles (2.262(2) Å).^[^ ^38^ ^]^ The slightly shorter Sn(IV)–C (2.158(3) Å) than the Sn(II)–C (2.243(3)) ) Å) bond length in **7** is in line with the formal oxidation state of tin.^[^ ^18b^ ^]^ Consistent with the presence of a stereoactive lone‐pair, the sum of the angles at the Sn(II) of **7** amounts to Σ = 273.39°. In **8** (Figure 4), the central Sn_2_P_2_ cluster remains intact and each of the tin atoms serves as a two‐electron σ‐donor ligand to bind with a Fe(CO)_4_ fragment. Thus, the HOMO and HOMO–2 of **5** (Figure 2c), which essentially depict a lone‐pair of electrons on the Sn centers, interact with the LUMO of Fe(CO)_4_ fragments in resulting **8**. The Sn–P (2.584(1)–2.648(1) Å) and C–Sn bond lengths (2.197(3); 2.206(2) Å) of **8** are slightly smaller compared to those of **5**. This is in line with the transfer of electron density from the tin to the iron atom. The Sn–Fe bond lengths of **8** (2.505(1) Å, 2.504(1) Å) are comparable to the known Sn(II)‐Fe(0) complexes (2.40–2.5 Å).^[^ ^39^ ^]^ The IR spectrum of **8** exhibits three absorption bands at 2050, 1995, and 1910 cm^−1^ for CO stretching vibrations.

## 3 Conclusion

In conclusion, we have shown the isolation of Sn_2_P_2_ cluster compound **5** embedded between two 1,3‐imidazole frameworks as green crystals. Reactivity of **5** has been demonstrated with CO_2_, selenium, and Fe_2_(CO)_9_. Compound **5** reversibly uptakes CO_2_ to form **6**. One CO_2_ molecule inserts into the Sn–P bond of **5** to result in a Sn–OC(=O)–P moiety in **6**. Under vacuum or at elevated temperature, **6** readily releases CO_2_ and regenerates **5**. Catalytic hydroboration of CO_2_ with **5** is presented. The mixed‐valent Sn(IV)/Sn(II) compound **7** and the stannylene‐Fe(0) complex **8** have been isolated as stable crystalline solids. All compounds have been characterized by multinuclear NMR spectroscopy and their solid‐state molecular structures have been unequivocally established by sc‐XRD.

## Conflict of Interest

The authors declare no conflict of interest.

## Supporting information

Supporting Information

Supporting Information

## Data Availability

The data that support the findings of this study are available in the supplementary material of this article.
