# Supplementary material for: Isolation of an Anionic Dicarbene Embedded Sn2P2 Cluster and Reversible CO2 Uptake
Source: Adv Sci (Weinh). 2023 Nov 28;11(5):2305545. doi: 10.1002/advs.202305545 (PMC10837339; doi:10.1002/advs.202305545)
Supplement: Supplementary file 1 — Supporting Information [file ADVS-11-2305545-s001.pdf]

ADVANCED SCIENCE
Open Access

Supporting Information

for *Adv. Sci.*, DOI 10.1002/advs.202305545

Isolation of an Anionic Dicarbene Embedded Sn_2P_2 Cluster and Reversible CO_2 Uptake

*Falk Ebeler, Yury V. Vishnevskiy, Beate Neumann, Hans-Georg Stammler and Rajendra S. Ghadwal**

Supporting Information

for

Isolation of an Anionic Dicarbene Embedded Sn_2P_2 Cluster and Reversible CO_2 Uptake

Falk Ebeler, Yury V. Vishnevskiy, Beate Neumann, Hans-Georg Stammler, and Rajendra S. Ghadwal*

Molecular Inorganic Chemistry and Catalysis, Inorganic and Structural Chemistry, Center for Molecular Materials, Faculty of Chemistry, Universität Bielefeld, Universitätsstrasse 25, D-33615, Bielefeld, German

E-Mail: rghadwal@uni-bielefeld.de; http://www.ghadwalgroup.de

Fax: +49 521 106 6026; Tel: +49 521 106 6167

.

Table of Contents

Experimental Section **S1**

Synthesis of [(ADC)SnBr]$_2$ (**1**) S1

Synthesis of [(ADC)SnPCO]$_2$ (**2**) and (**3a**) S2

Synthesis of [(ADC)SnP]$_2$ (**5**) S3

Reaction of [(ADC)SnP]$_2$ (**5**) with CO_2 to [(ADC)$_2${SnOC(O)P}SnP] (**6**) S4

Synthesis of [(ADC)$_2${Sn(Se)P}SnSeP]$_2$ (**7**) S5

Synthesis of [(ADC)SnP]$_2$[Fe(CO)$_4$]$_2$ (**8**) S6

Catalytic Studies **S7**

Plots of the NMR-Spectra **S13**

UV-Vis Spectra **S32**

Infrared Spectra **S33**

Crystallographic Details **S35**

Molecular Structures S38

Computational Details **S43**

References **S62**

Experimental Section

All experiments and manipulations were carried out under an inert gas atmosphere of argon or nitrogen using standard Schlenk techniques or an MBraun LABmaster Pro glovebox. THF, methyl *tert*-butyl ether (MTBE), toluene, benzene, *n*-hexane (NaK), fluorobenzene and acetonitrile (CaH_2) were dried by refluxing over an appropriate drying agent, distilled prior to use, and stored over 4 Å molecular sieve. Deuterated solvents were dried over appropriate drying agents, distilled, and stored inside a glove box. NMR spectra were recorded on a Bruker Avance III 500 or a III 500HD NMR spectrometer. Chemical shifts are given in δ ppm (d = doublet, t = triplet; pt = pseudo triplet, sept = septet, m = multiplet) and were referenced to the solvent residual peak: $CDCl_3$ (^{1}H, 7.26; ^{13}C, 77.16 ppm); THF-d_8 (^{1}H, 3.58; ^{13}C, 67.21 ppm); C_6D_6 (^{1}H, 7.16, ^{13}C, 128.06).[1] Infrared (IR) spectra were recorded on a Bruker Alpha FT-IR spectrometer equipped with an ATR module at room temperature under nitrogen atmosphere with 32 scans. UV-vis spectra were recorded on an Agilent 8453 UV visible spectroscopy system. (IPr^{Ph})Br (**1**) (IPr^{Ph} = PhC{(DippN)CH}$_2$; Dipp = 2,6-iPr$_2$C$_6$H$_3$) was prepared as described previously by this laboratory.[2] (1,4-dioxane)$_{2.5}$PCO was prepared using the literature reported method.[3] *n*BuLi (2.5 M in hexanes, Sigma Aldrich) and anhydrous tin(II) bromide (abcr), $Fe_2(CO)_9$ (Sigma Aldrich), and Se (Alfa Aesar) were used as received from the suppliers. Carbon dioxide was used in 4.5 purity.

Synthesis of [(ADC)SnBr]$_2$ (1)

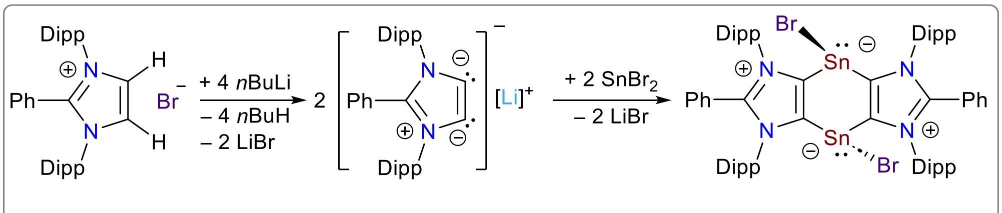

Compound **1** was prepared by adopting a similar method as reported previously by this laboratory for the chloride derivative.[4] To a 50 mL THF suspension of (IPr^{Ph})Br (5.20 g, 9.53 mmol) (IPr^{Ph} = PhC{(DippN)CH}$_2$; Dipp = 2,6-iPr$_2$C$_6$H$_3$) was added *n*BuLi (8.4 ml, 2.5 M, 21 mmol) at –60 °C. The resulting reaction mixture was allowed to warm up to room temperature and stirred for 45 min to obtain Li(ADC) (ADC = PhC{(DippN)C}$_2$; Dipp = 2,6-iPr$_2$C$_6$H$_3$) as a dark-brown solution.[5] This solution of Li(ADC) was transferred to 30 mL THF suspension of $SnBr_2$ (2.65 g, 9.58 mmol). The resulting light-brown mixture was stirred overnight at room temperature. The brown suspension was filtered and the colorless residue was washed with THF (3x 10 ml) to obtain [(ADC)SnBr]$_2$ (**1**) in 55% (3.48 g, 2.62 mmol) yield as a colorless solid. **^{1}H NMR** (500 MHz, CD_3Cl, 298 K): 7.42 (t, J = 7.7 Hz, 4H, p-C$_6H_3$), 7.33 (d, J = 7.2 Hz, 4H, m-C$_6H_3$), 7.14–7.07 (m, 6H, C$_6H_5$, m-C$_6H_3$), 6.97 (t, J = 7.9 Hz, 4H, C$_6H_5$), 6.75 (d, J = 8.0 Hz, 4H, C$_6H_5$), 3.14 (sept, J = 6.7 Hz, 4H, CH(CH$_3$)$_2$), 2.71 (sept, J = 6.6 Hz, 4H, CH(CH$_3$)$_2$), 1.34 (d, J = 6.7 Hz, 12H, CH(CH_3)$_2$), 1.23 (pt, 24H, CH(CH_3)$_2$), 0.19 (d, J = 6.8 Hz, 12H,

CH(CH_3)$_2$) ppm. **^{13}C{^{1}H} NMR** (126 MHz, $CDCl_3$, 298 K): 171.1 (*C*Sn), 146.2, 145.5, 134.7, 130.6 (*p*-*C*$_6$H$_3$, N*C*N), 129.7, 129.2, 128.1, 125.2, 124.7, 124.4 (*C*$_6$H$_3$, *C*$_6$H$_5$), 29.0, 28.2 (*C*H(CH$_3$)$_2$), 26.3, 25.8, 23.4, 23.2 (CH(*C*H$_3$)$_2$) ppm. **^{119}Sn{^{1}H} NMR** (186 MHz, $CDCl_3$, 298 K): –62.7 ppm.

Synthesis of [(ADC)SnPCO]$_2$ (2) and (3a)

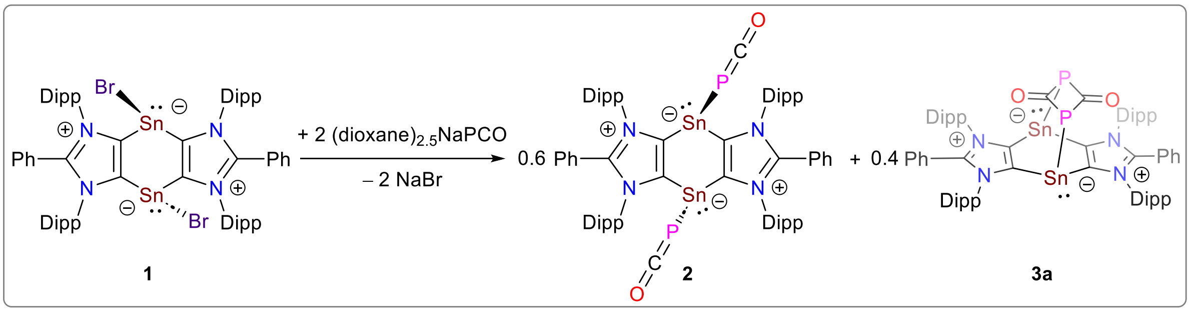

To a Schlenk flask containing [(ADC)SnBr]$_2$ (**1**) (2.00 g, 1.51 mmol) and (dioxane)$_{2.5}$NaPCO (0.91 g, 3.02 mmol) was added 80 mL THF. The resulting pale-yellow suspension was stirred at 50 °C for 12 h. Filtration though a plug of Celite and removal of the volatiles from the filtrate *in vacuo* afforded a yellow residue, which contained a mixture of compounds **2** and **3a** (in 6:4 ratio) as shown by NMR analyses (Figure S19). The mixture was suspended in 20 mL benzene and heated at 70 °C for 1 h with constant stirring. The insoluble material was separated by filtration, washed with benzene (3x 10 mL), and dried *in vacuo* to obtain compound **2** (1.61 g, 1.26 mmol, 84%) as a bright yellow solid **(see below for the workup with soluble part)*. Single crystals suitable for X-ray diffraction were grown by storing a concentrated THF solution of **2** (layered with 2 mL *n*-hexane) at –40 °C. Elemental analysis (%) calculated for (1282.8) $C_{68}H_{78}N_4O_2P_2Sn_2$ **2**: C 63.67, H 6.13, N 4.37; found: C 63.84, H 6.52, N 4.18. **^{1}H NMR** (500 MHz, C_6D_6, 298 K): 7.26 (d, J = 7.6 Hz, 4H, *m*-C$_6$*H*$_3$), 7.20 (t, J = 7.7 Hz, 4H, *p*-C$_6$*H*$_3$), 6.96 (d, J = 7.5 Hz, 4H, C$_6$*H*$_5$), 6.87 (d, J = 7.8 Hz, 4H, *m*-C$_6$*H*$_3$), 6.59 – 6.50 (m, 6H, C$_6$*H*$_5$), 3.95 (sept, J = 6.9 Hz, 4H, C*H*(CH$_3$)$_2$), 2.93 (sept, J = 6.8 Hz, 4H, C*H*(CH$_3$)$_2$), 1.70 (d, J = 6.6 Hz, 12H, CH(C*H*$_3$)$_2$), 1.46 (d, J = 6.5 Hz, 12H, CH(C*H*$_3$)$_2$), 1.35 (d, J = 6.8 Hz, 12H, CH(C*H*$_3$)$_2$), 0.20 (d, J = 6.7 Hz, 12H, CH(C*H*$_3$)$_2$) ppm. **^{13}C{^{1}H} NMR** (126 MHz, C_6D_6, 298 K): 176.6 (SnP*C*O), 167.3 (*C*Sn), 146.3, 145.3, 145.1, 134.9, 130.4 (*p*-*C*$_6$H$_3$, N*C*N), 129.3, 125.3, 124.8 (*m*-*C*$_6$H$_3$, *C*$_6$H$_5$), 28.8, 28.1 (*C*H(CH$_3$)$_2$), 26.5, 24.9, 23.9, 23.0 (CH(*C*H$_3$)$_2$) ppm. **^{31}P{^{1}H} NMR** (202 MHz, C_6D_6, 298 K): –360.8 ppm. **^{119}Sn{^{1}H} NMR** (187 MHz, C_6D_6, 298 K): –165.5 (d, $^1J_{PSn}$ = 643.5 Hz). **FTIR** (in THF): $\tilde{\nu}$ =1860 cm^{-1} (s, C=O).

An NMR sample of **2** stored at room temperature in day light indicated the 30% conversion of **2** into **3a** (**2**:**3a** = 70:30) after two days. Upon warming the sample at 70°C for 1h, the amount of **3a** was diminished significantly (**2**: **3a** = 90:10), indicating the thermal rearrangement of **3a** into **2**.

The filtrate containing mostly **3a** with a small amount of **2** was concentrated and stored at 10 °C for one week (see below).*

***The benzene soluble part was stored at 5 °C, leading to the formation of pale yellow crystals, which were analyzed by single crystal X-ray diffraction (sc-XRD). There were mostly crystals of **2**, but differently appearing crystals were picked up by visual inspection and analyzed by sc-XRD. See X-ray crystallographic details for **3a** and the isomer **3b**. It should be noted that **3b** was not identified in solutions by NMR studies, presumably compound **3b** may be the result of crystal packing effects.

A pure sample containing only **3a** (i.e. free from **2**) could not be obtained due to a rather similar solubility of **2** and **3a**. Therefore, NMR signals for **3a** were tentatively assigned by excluding the signals due to **2** from the spectra measured for a mixture comprising of **2** and **3a**.

NMR Data of 3 (See Figures S19-22: The signals of **2** from the spectral plots were excluded.). **^{1}H NMR** (500 MHz, THF-d_8, 298 K): 7.20 (overlapping signals, 4H, *m*-C_6H_3), 7.08 (t, *J* = 7.6 Hz, 4H, *p*-C_6H_3), 7.03 (d, *J* = 7.3 Hz, 4H, *m*-C_6H_3), 6.92 (d, *J* = 7.6 Hz, 4H, C_6H_5), 6.63 – 6.55 (m, 6H, C_6H_5), 3.28 (sept, *J* = 6.6 Hz, 4H, C*H*(CH_3)$_2$), 2.54 (sept, *J* = 6.8 Hz, 4H, C*H*(CH_3)$_2$), 1.23 (d, *J* = 6.7 Hz, 12H, CH(C*H*$_3$)$_2$), 1.18 (d, *J* = 6.2 Hz, 12H, CH(C*H*$_3$)$_2$), 0.99 (d, *J* = 6.8 Hz, 12H, CH(C*H*$_3$)$_2$), 0.80 (d, *J* = 6.6 Hz, 12H, CH(C*H*$_3$)$_2$) ppm. **^{1}H NMR** (500 MHz, C_6D_6): 7.26 (overlapped signal 4H, *m*-C_6H_3), 7.08 (t, *J* = 7.6 Hz, 4H, *p*-C_6H_3), 7.03 (d, *J* = 7.3 Hz, 4H, *m*-C_6H_3), 6.92 (d, *J* = 7.6 Hz, 4H, C_6H_5), 6.63 – 6.55 (m, 6H, C_6H_5), 3.71 (sept, *J* = 6.3 Hz, 4H, C*H*(CH_3)$_2$), 2.69 (sept, *J* = 6.9 Hz, 4H, C*H*(CH_3)$_2$), 1.56 (d, *J* = 6.7 Hz, 12H, CH(C*H*$_3$)$_2$), 1.27 (d, *J* = 6.9 Hz, 12H, CH(C*H*$_3$)$_2$), 0.97 (pt, 24H, CH(C*H*$_3$)$_2$) ppm. **^{13}C{^{1}H} NMR** (126 MHz, THF-d_8, 298 K): 184.4 (SnP*C*O), 183.7 (SnP*C*O), 167.5 (*C*Sn), 149.0, 147.9, 145.3, 136.4, 135.5, 131.1, 130.8, 130.7, 130.5, 130.4, 130.2, 128.6, 128.4, 125.9, 125.8, 125.4, 125.2, 124.5 (*C*$_6$H$_3$, *C*$_6$H$_5$, N*C*N), 30.0, 29.4 (*C*H(CH_3)$_2$), 26.2, 24.1, 23.3, 23.2 (CH(*C*H$_3$)$_2$) ppm. **^{31}P{^{1}H} NMR** (202 MHz, THF-d_8, 298 K): 170.1 ppm. **^{119}Sn{^{1}H} NMR** (187 MHz, THF-d_8, 298 K): –148.1 (d, $^1J_{PSn}$ = 663.7 Hz) ppm.

Synthesis of [(ADC)SnP]$_2$ (5)

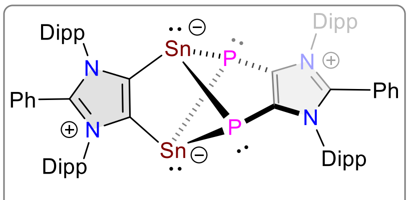

5

A Schlenk flask containing [(ADC)Sn(PCO)]$_2$ (**2**) (1.90 g, 1.48 mmol) in fluorobenzene (15 ml) was irradiated with UV light (λ = 360 nm) for 4h, resulting in a dark green solution The volatiles from the dark green solution were removed *in vacuo*. The resulting solid material was washed with *n*-hexane and dried *in vacuo* to obtain **5** (1.62 g, 1.32 mmol, 90%) as a green solid. Single crystals suitable for sc-XRD were grown by storing a concentrated benzene solution of **6** at 10 °C for one week. Elemental analysis (%) calculated for $C_{66}H_{78}N_4P_2Sn_2$ (1226.8) **5**: C, 64.62; H, 6.41; N, 4.57; found: C, 64.38, H, 6.62, N, 4.11. **^{1}H NMR** (500 MHz, THF-d_8, 298 K): 7.40–7.31 (m, 4H, *p*-C_6H_3), 7.21 (pt, 8H, *m*-C_6H_3), 7.15 (d, *J* = 7.3 Hz, 1H, C_6H_5), 7.09 (t, *J* = 7.7 Hz, 2H, C_6H_5), 7.05 (d, *J* = 8.0 Hz, 2H, C_6H_5), 6.91 – 6.90 (m, 3H, C_6H_5), 6.80 (d, *J* = 7.6 Hz, 2H, C_6H_5), 3.23 (sept, *J* = 6.8 Hz, 4H, C*H*(CH_3)$_2$), 2.95 (sept, *J* = 6.4 Hz, 4H, C*H*(CH_3)$_2$), 1.36 (d, *J* = 6.7 Hz, 12H, CH(C*H*$_3$)$_2$), 1.16 (d, *J* = 6.7 Hz, 12H,

$CH(CH_3)_2$), 1.04 (d, J = 6.9 Hz, 12H, $CH(CH_3)_2$), 1.01 (d, J = 6.9 Hz, 12H, $CH(CH_3)_2$) ppm. **$^{13}C\{^1H\}$ NMR (**126 MHz, THF-d_8, 298 K): 200.9 (*C*Sn), 145.4 (*C*P), 137.0, 135.7, 131.9, 130.3 (*p*-C_6H_3, N*C*N), 130.0, 129.2, 128.7, 128.4, 128.1, 127.9, 125.6 (C_6H_5, C_6H_3), 124.8, 124.7, 124.1 (*m*-C_6H_3), 29.5, 29.4 ($CH(CH_3)_2$), 23.1, 23.0 ($CH(CH_3)_2$) ppm. **$^{31}P\{^1H\}$ NMR** (202 MHz, THF-d_8, 298 K): –110.4 ppm. **$^{119}Sn\{^1H\}$ NMR** (187 MHz, THF-d_8, 298 K): 748.1 (t, J = 416.1 Hz) ppm**. UV/Vis** (THF, λ (nm) (ε (M-1 cm-1))): 306 (9080), 420 (3212), 688 (950).

Reaction of $[(ADC)SnP]_2$ (5) with CO_2 to $[(ADC)_2\{SnOC(O)P\}SnP]$ (6)

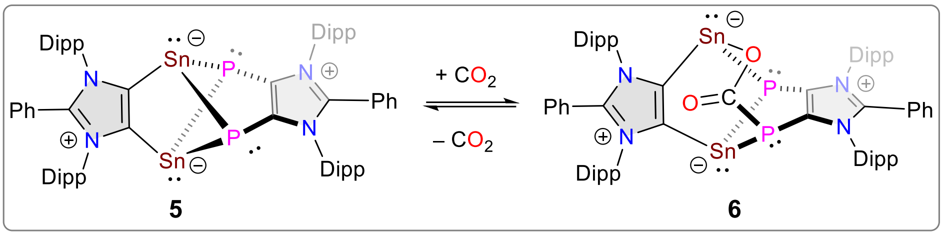

A C_6D_6 solution of **5** (50 mg, 0.04 mmol) in a J-Young NMR tube was exposed to CO_2 (1 atm) at rt. The dark green solution of **6** slowly turned wine red over the course of 3h. The ^1H NMR spectrum measured after CO_2 exposure indicated the conversion of **5** into **6** by 30% after 10 min, 50% after 1h, and 85% after 3h. On warming the reaction mixture at 60 °C in a closed vessel, the amount of **5** was increased, while cooling the vessel to room temperature led to formation of **6** up to 85%. Removal of the volatiles under vacuum led to the restoration of green color and the quantitative formation of **5**, indicating reversible binding of CO_2 with **5**. Single crystals of **6** suitable for X-ray diffraction were grown by storing a toluene solution (containing **5** and **6** in 1:5 ratio) with CO_2 atmosphere (1 atm) at –24 °C for one week. The NMR data for **6** were extracted by excluding the signals for **5** measured for a mixture of **5** and **6** (in 15:85 ratio).

^1H NMR* (600 MHz, C_6D_6, 298 K): 7.25–7.20 (m, 2H, C_6H_3), 7.11–7.02 (m, 10H, C_6H_5, C_6H_3), 6.99–6.98 (m, 2H, C_6H_5, C_6H_3), 6.92 (dd, J = 7.4, 1.8 Hz, 1H, C_6H_3), 6.85 (dd, J = 7.6, 1.6 Hz, 1H, C_6H_3), 6.82 (dd, J = 7.2, 1.9 Hz, 1H, C_6H_3).6.60–6.57 (m, 2H, C_6H_3), 6.54–6.49 (m, 3H, C_6H_5), 3.96 (sept, J = 6.6 Hz, 1H, $CH(CH_3)_2$), 3.82 (sept, J = 6.7 Hz, 1H, $CH(CH_3)_2$), 3.64 (sept, J = 6.7 Hz, 1H, $CH(CH_3)_2$), 3.24 (sept, J = 6.1 Hz, 1H, $CH(CH_3)_2$), 3.13 (sept, J = 6.8 Hz, 1H, $CH(CH_3)_2$), 3.03 (sept, J = 6.8 Hz, 1H, $CH(CH_3)_2$), 2.63 (sept, J = 6.9 Hz, 1H, $CH(CH_3)_2$), 2.54 (sept, J = 6.8 Hz, 1H, $CH(CH_3)_2$), 1.79 (d, J = 6.5 Hz, 3H, $CH(CH_3)_2$), 1.74–1.73 (m, 6H, $CH(CH_3)_2$), 1.66 (pt, 3H, $CH(CH_3)_2$), 1.54 (d, J = 6.7 Hz, 3H, $CH(CH_3)_2$), 1.35–1.32 (m, 6H, $CH(CH_3)_2$), 1.24 (d, J = 6.8 Hz, 3H, $CH(CH_3)_2$), 1.19–1.15i (m, 12H, $CH(CH_3)_2$), 0.84 (d, J = 6.9 Hz, 3H, $CH(CH_3)_2$), 0.75-0.73 (m, 6H, $CH(CH_3)_2$), 0.69 (d, J = 6.8 Hz, 3H, $CH(CH_3)_2$) ppm. **$^{13}C\{^1H\}$ NMR** (151 MHz, C_6D_6, 298 K): 196.0 (*C*Sn), 194.0 (*C*Sn), 189.1 (dd, J = 38.4, 3.4 Hz PCO_2), 162.39 (dd, J = 66.6, 5.5 Hz, NCP), 152.5 (dd, J = 55.1, 10.3 Hz), 147.3, 147.2, 146.9, 146.5, 145.6, 145.5, 145.2, 145.1, 144.6, 144.5, 142.7, 137.1, 136.9, 134.1, 133.9, 130.9, 130.7, 130.5, 130.3, 129.9, 129.8, 129.2, 129.1, 128.6, 125.5, 125.4, 125.3, 125.1, 124.9, 124.9, 124.8, 124.3,

124.2, 123.9, 123.8 (C_6H_3, C_6H_5, NCN), 29.7, 29.3, 29.2, 29.1, 28.9, 28.2, 28.1 ($CH(CH_3)_2$), 26.8, 26.7, 26.2, 26.1, 25.8, 25.4, 25.3, 25.2, 24.0, 23.9, 23.8, 23.5, 23.2, 22.7, 22.6 $CH(CH_3)_2$). **^{31}P{^{1}H} NMR** (202 MHz, C_6D_6, 298 K): –74.2 (d, $^2J_{P\text{-}P}$ = 25.4 Hz, SnPCO$_2$), –171.1 (d, $^2J_{P\text{-}P}$ = 25.4 Hz, PSnO) ppm.**^{119}Sn{^{1}H} NMR** (224 MHz, C_6D_6, 298 K): 466.6 (d, $^1J_{Sn\text{-}P}$ = 666.2 Hz, OSnP), 373.9 (pt, $^1J_{Sn\text{-}P}$ = 663.2 Hz, SnP$_2$) ppm. **FTIR** (in THF): $\tilde{\nu}$ =1670 cm^{-1} (s, C=O).

*The signals of **5** are excluded.

Synthesis of [(ADC)$_2${Sn(Se)P}SnSeP]$_2$ (7)

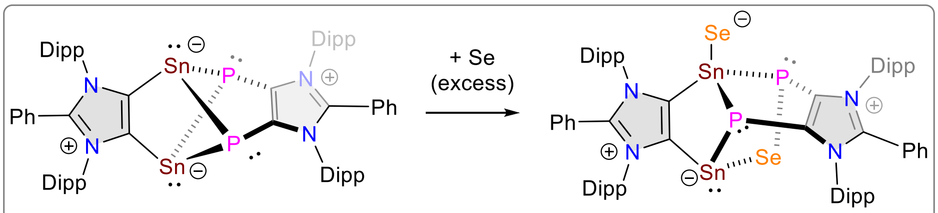

5 **7**

To a Schlenk flask containing [(ADC)SnP]$_2$ (**5**) (0.30 g, 0.24 mmol) and Se (0.08 g, 1.01 mmol), was added flourobenzene (10 ml) at –60°C. The resulting reaction mixture was stirred overnight at room temperature. The dark red suspension was filtered through a plug of Celite. The volatiles were removed *in vacuo* and the residue was suspended in Et_2O. Filtration of the red suspension and the subsequent washing with Et_2O (3x 10 ml) afforded **7** (0.29 g, 0.21 mmol, 88%) as a red solid after drying under vacuum. Single crystals suitable for X-ray diffraction were obtained by storing a pyridine-acetonitrile (1:3) solution of **7** at –40 °C. Elemental analysis (%) calculated for $C_{67}H_{81}N_4P_2Se_2Sn_2$ (1399.7) **7**: C, 57.49; H , 5.83; N, 4.00; found: C, 56.97, H, 5.72, N, 4.10. **^{1}H NMR** (500 MHz, THF-d_8, 298 K): 7.52 – 7.43 (m, 5H, C_6H_3), 7.36 – 7.33 (m, 2H, C_6H_3), 7.28 (d, J = 7.9 Hz, 1H, C_6H_3), 7.21 – 7.17 (m, 5H, C_6H_3), 7.07 – 7.01 (m, 5H, C_6H_5), 6.94 (d, J = 8.1 Hz, 2H, C_6H_5), 6.88 (d, J = 7.9 Hz, 2H, C_6H_5), 3.14 (sept, J = 6.6 Hz, 1H, $CH(CH_3)_2$), 2.93 (sept, J = 6.8 Hz, 1H, $CH(CH_3)_2$), 2.58 (sept, J = 6.8 Hz, 1H, $CH(CH_3)_2$), 2.46 (sept, J = 6.5 Hz, 1H, $CH(CH_3)_2$), 2.40 (sept, J = 6.8 Hz, 1H, $CH(CH_3)_2$), 2.34 (sept, J = 6.9 Hz, 1H, $CH(CH_3)_2$), 2.24 (sept, J = 6.7 Hz, 1H, $CH(CH_3)_2$), 2.19 (sept, J = 6.8 Hz, 1H, $CH(CH_3)_2$), 1.65 (d, J = 6.6 Hz, 3H, $CH(CH_3)_2$), 1.45 (d, J = 6.7 Hz, 3H, $CH(CH_3)_2$), 1.42 – 1.34 (m, 12H, $CH(CH_3)_2$), 1.30 – 1.24m (m, 12H, $CH(CH_3)_2$), 1.16 (d, J = 6.7 Hz, 3H, $CH(CH_3)_2$), 1.12 (d, J = 6.9 Hz, 3H, $CH(CH_3)_2$), 0.66 (d, J = 6.9 Hz, 3H, $CH(CH_3)_2$), 0.45 (d, J = 6.9 Hz, 3H, $CH(CH_3)_2$), 0.41 (d, J = 6.9 Hz, 3H, $CH(CH_3)_2$), 0.36 (d, J = 6.9 Hz, 3H, $CH(CH_3)_2$) ppm. **^{13}C{^{1}H} NMR** (126 MHz, THF-d_8, 298 K): 170.2 (CSn), 148.55 (CP), 147.9 (CC_6H_5), 146.6 (C_6H_3), 146.5, 146.3, 146.1, 145.9, 145.6, 145.4, 145.4, 144.6, 134.8 (C_6H_5, C_6H_3, NCN), 134.1, 133.5, 133.1, 131.6, 131.4, 131.4, 131.2, 131.0, 130.9, 130.3, 130.0, 129.8, 128.8, 128.70, 125.8, 125.5, 125.4, 125.4, 125.3, 125.1, 125.0, 124.9, 124.3, 124.1, 123.8 (C_6H_5, C_6H_3), 30.1, 29.7, 29.5, 29.2, 29.1, 29.0 ($CH(CH_3)_2$), 27.1, 27.0, 26.6, 26.5, 26.2, 26.1, 26.0, 25.6, 24.1, 23.9, 23.6, 23.3, 23.1, 22.7 ($CH(CH_3)_2$) ppm. **^{31}P{^{1}H} NMR** (202 MHz, THF-d_8, 298 K): –110.1 (d, $^2J_{P\text{-}P}$ = 27.3 Hz, SnPSe), –121.5 (d, $^2J_{P\text{-}P}$ = 27.2 Hz, SnPSn) ppm. **^{77}Se NMR** (95 MHz,

THF-d_8, 298 K): 64.4 (dd, J = 231.5, 17.8 Hz, Sn*Se*P), 292.1 (t. J = 10.3, *Se*SnP) ppm. **^{119}Sn{^{1}H} NMR** (186 MHz, THF-d_8, 298 K): 78.1 (dd, $^1J_{Sn-P}$ = 804.2, 693.3 Hz, Se*Sn*P), –66.5 (dd, $^1J_{Sn-P}$ = 809.7, 72.6 Hz, P*Sn*Se) ppm.

Synthesis of [(ADC)SnP]$_2$[Fe(CO)$_4$]$_2$ (8)

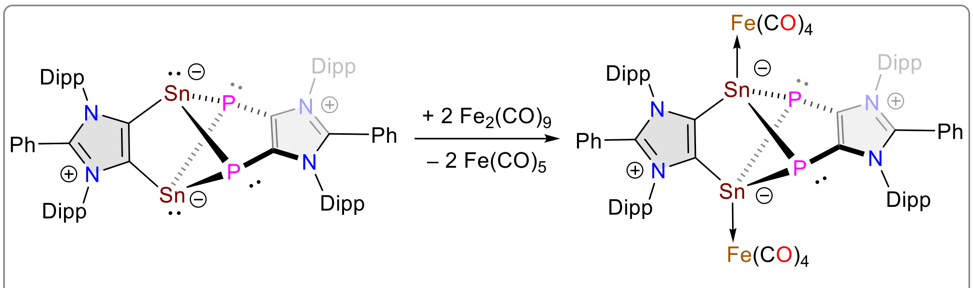

5 **8**

To a Schlenk flask containing [(ADC)SnP]$_2$ (**5**) (0.25 g, 0.20 mmol) and Fe$_2$(CO)$_9$ (0.15 g, 0.41 mmol) was added 15 mL THF at –60°C. The resulting reaction mixture was stirred at room temperature overnight. The volatiles from the red solution were removed *in vacuo* and the residue was suspended in MTBE. Filtration of the brown suspension afforded **8** (0.15 g, 0.10 mmol, 50%) as a red-brown solid. Single crystals suitable for X-ray diffraction were obtained by storing a concentrated solution of **8** in THF at –40 °C. Elemental analysis (%) calculated for $C_{74}H_{78}N_4Fe_2O_8P_2Sn_2$ (1562.5) **8**: C, 56.88; H, 5.03; N, 3.59; found: C, 56.62, H, 4.93, N, 3.67. **^{1}H NMR** (500 MHz, THF-d_8, 298 K): 7.50–7.41 (m, 4H, *p*-C$_6$*H*$_3$), 7.30 (d, J = 7.8 Hz, 8H, *m*-C$_6$*H*$_3$), 7.28–7.19 (m, 2H, C$_6$*H*$_5$), 7.13–7.05 (m, 4H, C$_6$*H*$_5$), 7.02 (d, J = 7.7 Hz, 2H, C$_6$*H*$_5$), 6.75 (d, J = 7.9 Hz, 2H, C$_6$*H*$_5$), 2.83 (sept, J = 6.6 Hz, 4H, C*H*(CH$_3$)$_2$), 2.74 (sept, J = 6.8 Hz, 4H, C*H*(CH$_3$)$_2$), 1.35 (pt, J = 6.9 Hz, 24H, CH(C*H*$_3$)$_2$), 0.99 (d, J = 6.7 Hz, 12H, CH(C*H*$_3$)$_2$), 0.78 (d, J = 6.8 Hz, 12H, CH(C*H*$_3$)$_2$) ppm. **^{13}C{^{1}H} NMR** (126 MHz, THF-d_8, 298 K): 217.0 (*C*O), 166.3 (*C*Sn), 147.3 (N*C*N), 146.6, (*i*-C$_6$H$_3$), 133.6 (d, J = 10.9 Hz, *C*P), 131.8 (*p*-*C*$_6$H$_3$), 130.5, 130.0, 129.1, 128.8 (*C*$_6$H$_5$), 126.1, 125.8 (*m*-*C*$_6$H$_3$), 124.5, 123.4, 29.7, 29.7 (*C*H(CH$_3$)$_2$), 26.4, 23.3, 23.2 (CH(*C*H$_3$)$_2$) ppm. **^{31}P{^{1}H} NMR** (202 MHz, THF-d_8, 298 K): 0.8 ppm. **^{119}Sn{^{1}H} NMR** (187 MHz, THF-d_8, 298 K): 494.9 (t, $^1J_{P-Sn}$ = 493.8 Hz) ppm. **FTIR** (in THF): $\tilde{\nu}$ =2050, 1995, 1910 cm^{-1} (s, C=O).

Catalytic Studies

General Procedure for the Hydroboration of Carbon Dioxide

Preparation of Stock Solutions: The reference 1,2,4,5-tetramethylbenzene (50 mg, 0.37 mmol) was dissolved in 1 ml C_6D_6 to obtain a 0.37 mol/l stock solution. **5** (25 mg, 0.020 mmol) was dissolved in 1 ml C_6D_6 to obtain a 0.02 mol/l stock solution. HBpin (96%, 128 mg, 1.00 mmol) was dissolved in 1 ml C_6D_6 to obtain a 1 mol/l stock solution.

Control Experiment without Catalyst: A J-Young NMR tube with 1,2,4,5-tetramethylbenzene (100 µl, 0.037 mmol, internal standard), HBpin (200 µl, 0.20 mmol) and C_6D_6 (300 µl) was freeze-pump-thaw degassed twice and then exposed to CO_2 gas (1 atm) and subsequently heated (70 °C) for 24 h. No reaction between CO_2 and HBpin was observed based on ^{1}H and ^{11}B NMR analyses.

General Procedure for the Hydroboration of Carbon Dioxide: A J-Young NMR tube containing **5** (100 µl, 0.002 mmol), 1,2,4,5-tetramethylbenzene (100 µl, 0.037 mmol, internal standard) and HBpin (200 µl, 0.20 mmol; 40 µl, 0.04 mmol, respectively) in C_6D_6 (200 µl; 360 µl, respectively) was freeze-pump-thaw degassed twice, exposed to CO_2 gas (1 atm), where applicable heated (70 °C), and monitored *via* ^{1}H and ^{11}B- NMR until full consumption of HBpin. The conversion of HBpin and formation of HC(O)OBpin, $CH_2O(BPin)_2$, CH_3OBPin and $O(Bpin)_2$ was calculated in relation to the integral of the internal standard.

^{1}H/^{11}B NMR Chemical Shifts for the Literature known Boron compounds:[6]

HC(O)OBpin: 8.21 (s, 1H, *H*CO_2B) 0.91 (s, 12H, Bpin-CH_3) ppm.
$CH_2(OBPin)_2$: 5.51 (s, 2H, CH_2), 1.01 (s, 24H, Bpin-CH_3)/ 22.8 ppm.
CH_3OBPin: 3.50 (s, 3H, O-CH_3), 1.04 (s, 12H, Bpin-CH_3)/ 20.3 ppm.
(BPin)O(Bpin): 1.02 (s, 24H, Bpin-CH_3)/ 20.8 ppm.

Table S1. Catalytic hydroboration of CO_2 with **6**.

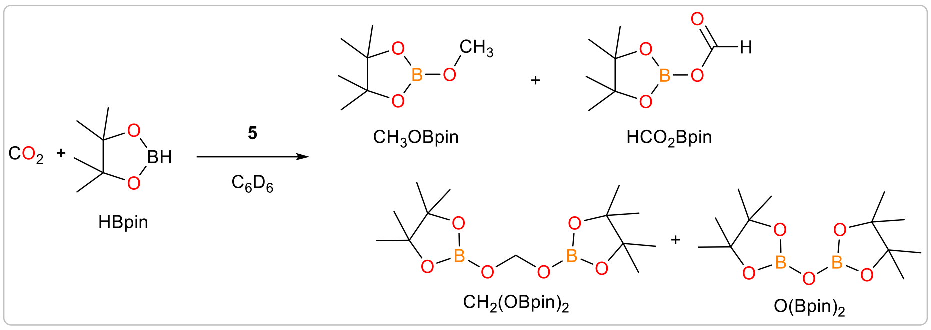

	5 mol%, 70 °C, 10 min	5 mol%, 20 °C, 20 h	1 mol%, 70 °C, 2h	1 mol%, 70 °C,1h
**HCO_2BPin**	58%	62%	/	14%
**$CH_2(OBPin)_2$**	2%	3%	23%	18%
**CH_3OBpin**	1%	1%	28%	14%

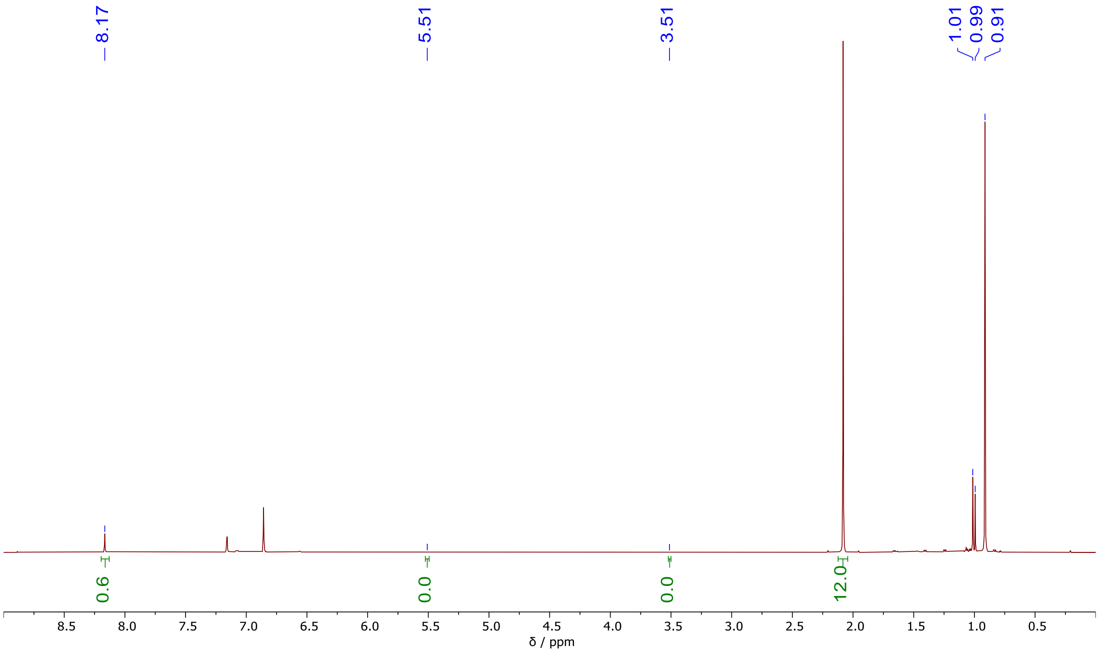

Figure S1. ^{1}H NMR (500 MHz, C_6D_6, 298 K) spectrum of CO_2 hydroboration with **5** (5 mol%) at 70 °C for 10 min.

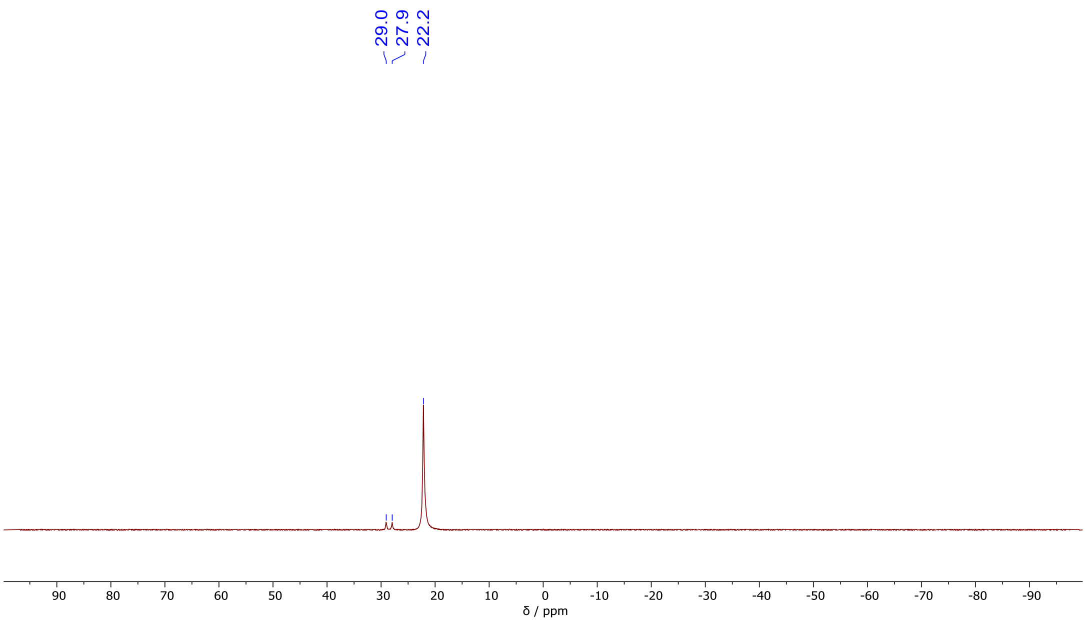

Figure S2. ^{11}B NMR (160 MHz, C_6D_6, 298 K) spectrum of CO_2 hydroboration with **5** (5 mol%) at 70 °C for 10 min.

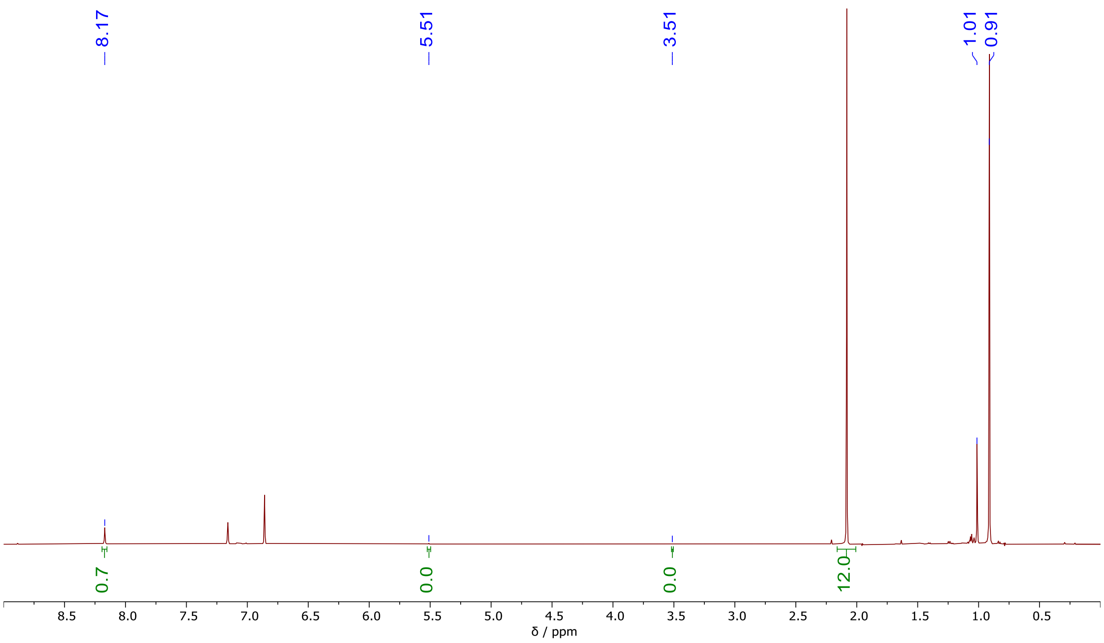

Figure S3. ^{1}H NMR (500 MHz, C_6D_6, 298 K) spectrum of CO_2 hydroboration with **5** (5 mol%) at 20 °C for 20 h.

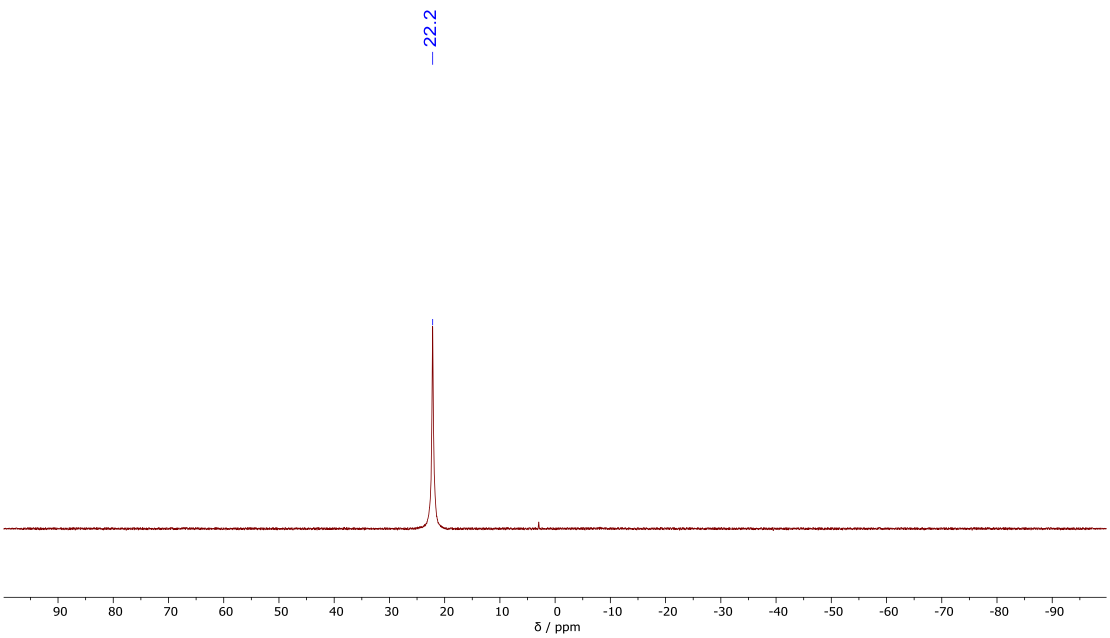

Figure S4. ^{11}B NMR (160 MHz, C_6D_6, 298 K) spectrum of CO_2 hydroboration with **5** (5 mol%) at 20 °C for 20 h.

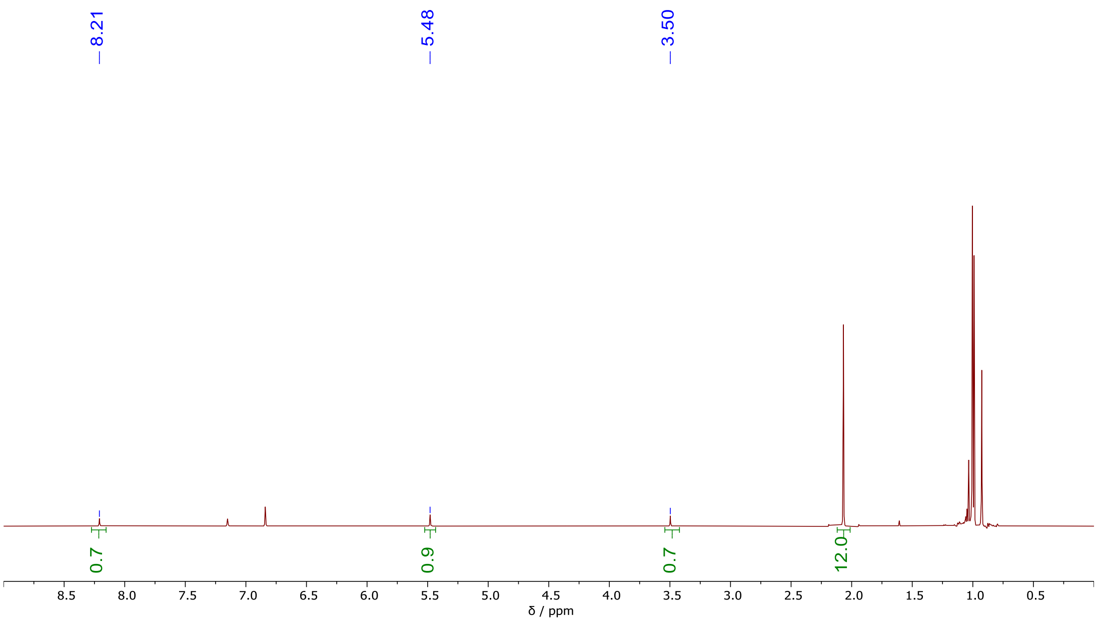

Figure S5. ^{1}H NMR (500 MHz, C_6D_6, 298 K) spectrum of CO_2 hydroboration with **5** (1 mol%) at 70 °C for 1h.

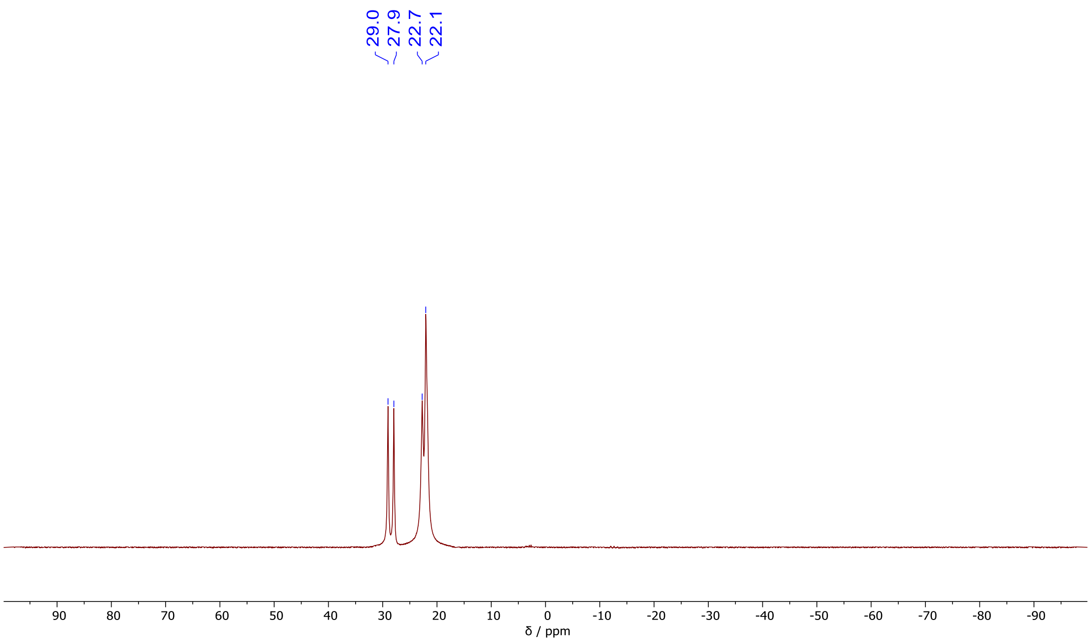

Figure S6. ^{11}B NMR (160 MHz, C_6D_6, 298 K) spectrum of CO_2 hydroboration with **5** (1 mol%) at 70 °C for 1h.

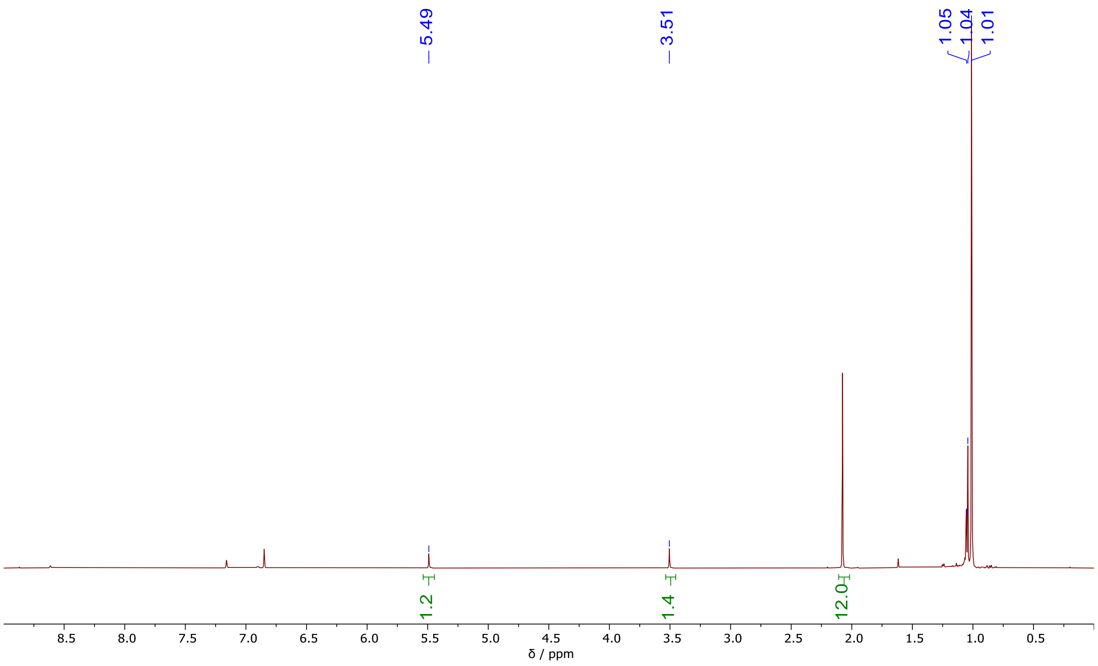

Figure S7. ^{1}H NMR (500 MHz, C_6D_6, 298 K) spectrum of CO_2 hydroboration with **5** (1 mol%) at 70 °C for 2h.

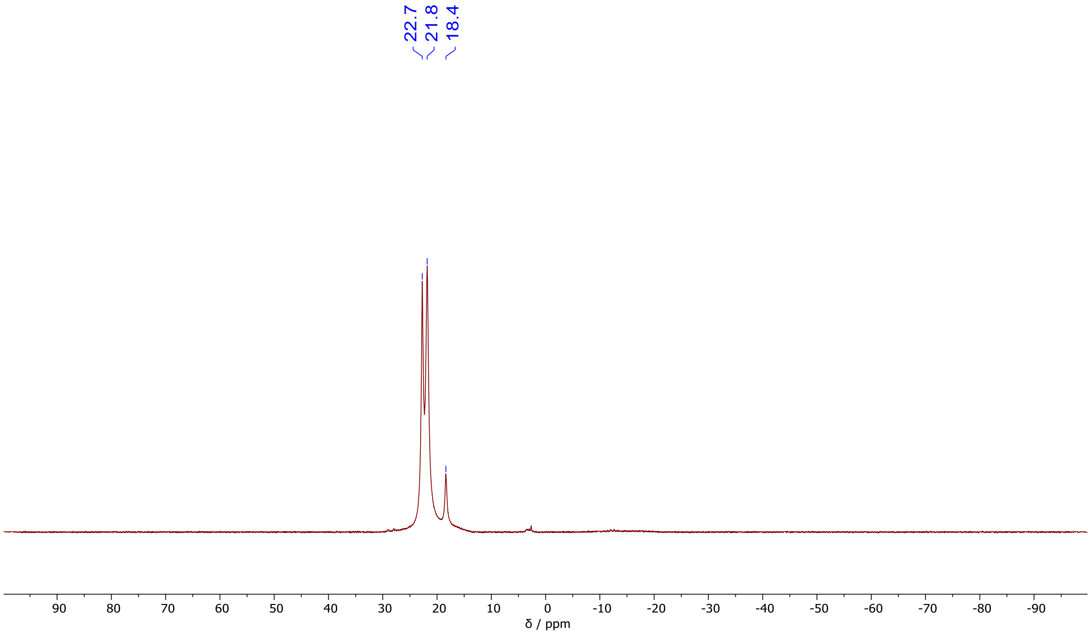

Figure S8. ^{11}B NMR (160 MHz, C_6D_6, 298 K) spectrum of CO_2 hydroboration with **5** (1 mol%) at 70 °C for 2h.

Plots of the NMR-Spectra

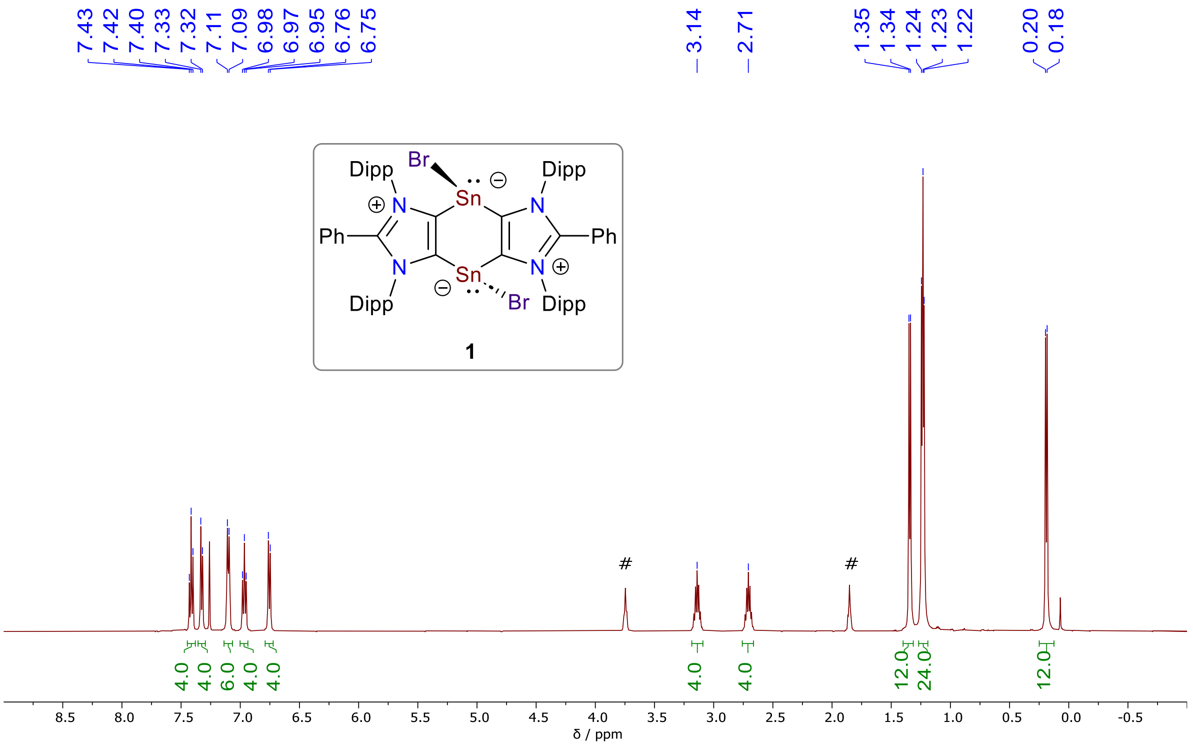

Figure S9. ^{1}H NMR (500 MHz, $CDCl_3$, 298 K) spectrum of compound **1**.(#THF).

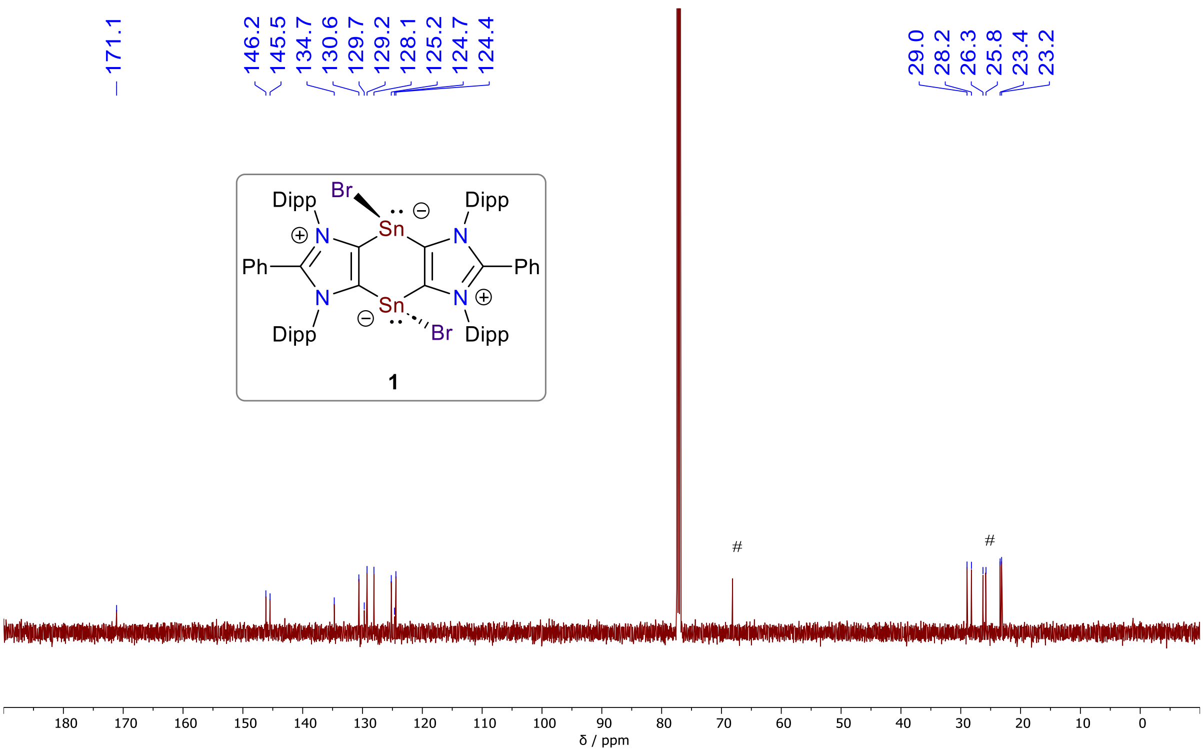

Figure S10. ^{13}C{1H} NMR (126 MHz, $CDCl_3$, 298 K) spectrum of compound **1**.(#THF).

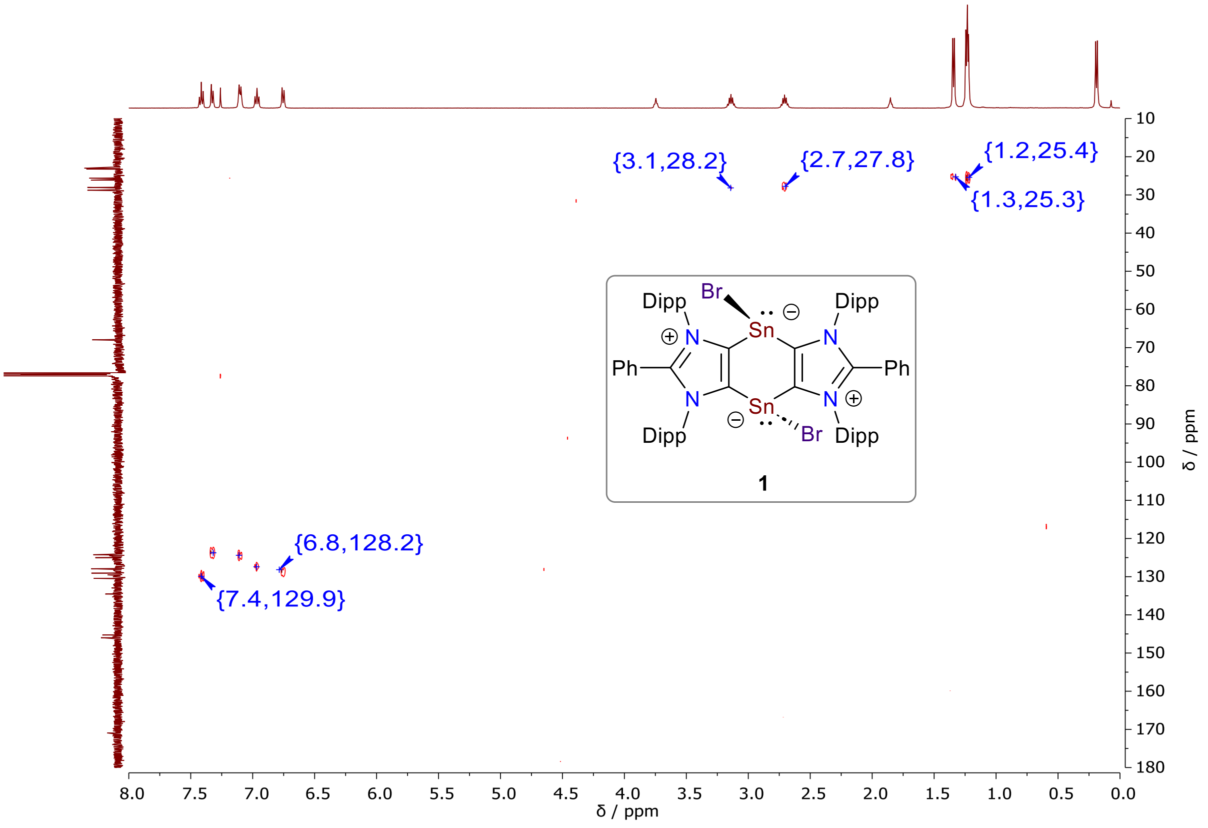

Figure S11. ^{1}H-^{13}C HMQC NMR ($CDCl_3$, 298 K) spectrum of compound **1**.

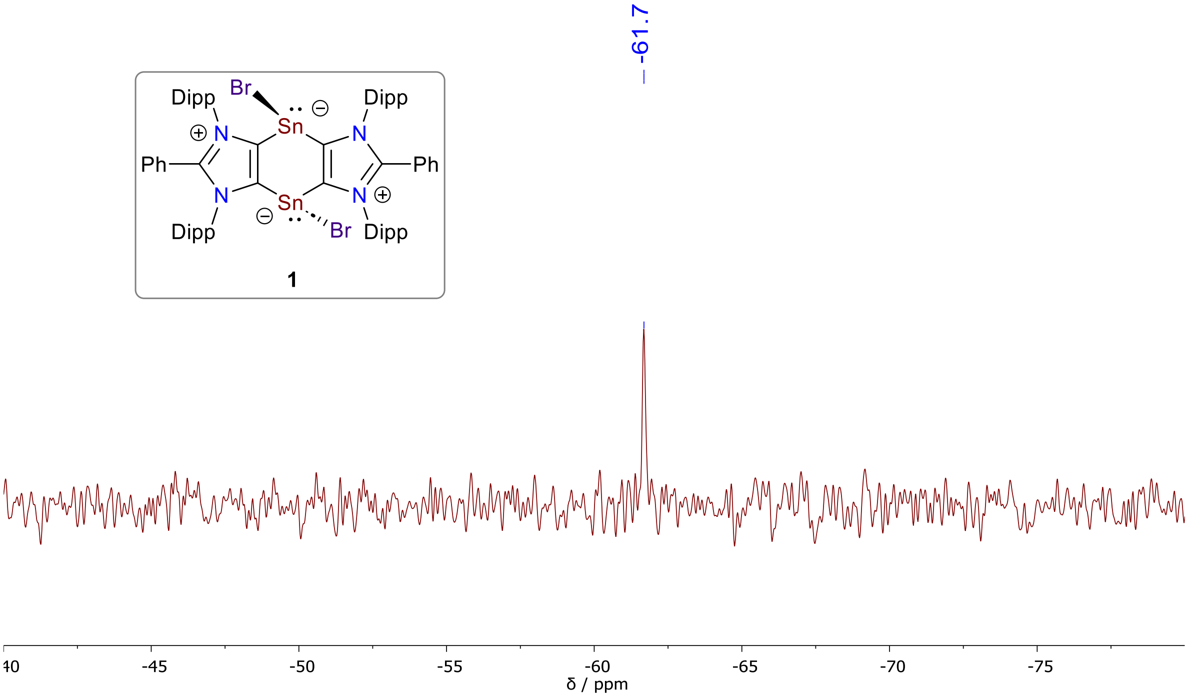

Figure S12. ^{119}Sn NMR (186 MHz, $CDCl_3$, 298 K) spectrum of compound **1**.

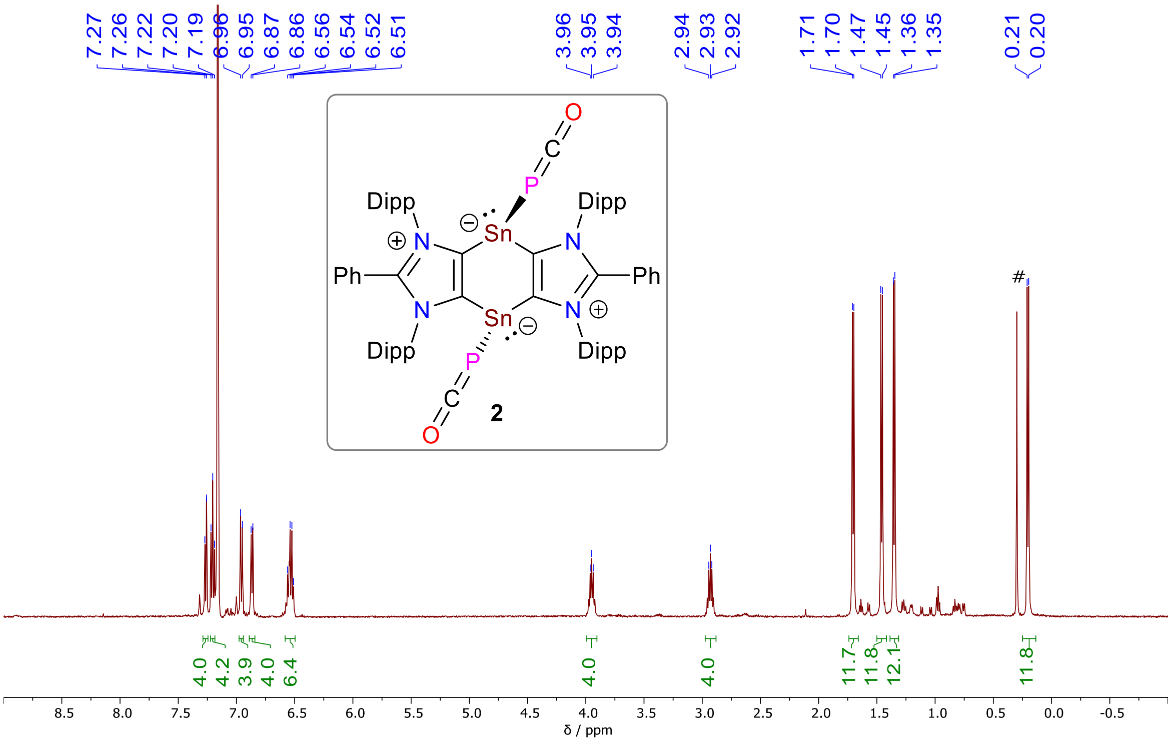

Figure S13. ^{1}H NMR (500 MHz, C_6D_6, 298 K) spectrum of compounds **2**.(#grease).

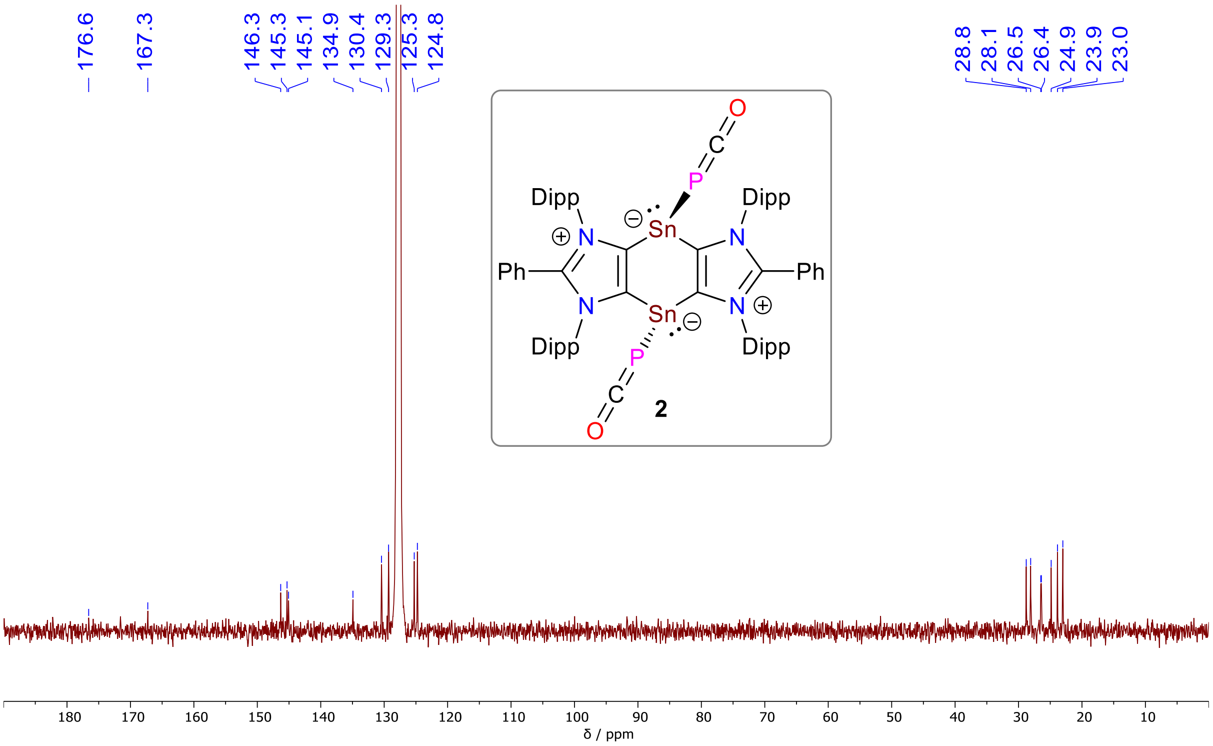

Figure S14. ^{13}C{^{1}H} NMR (126 MHz, C_6D_6, 298 K) spectrum of compound **2**.

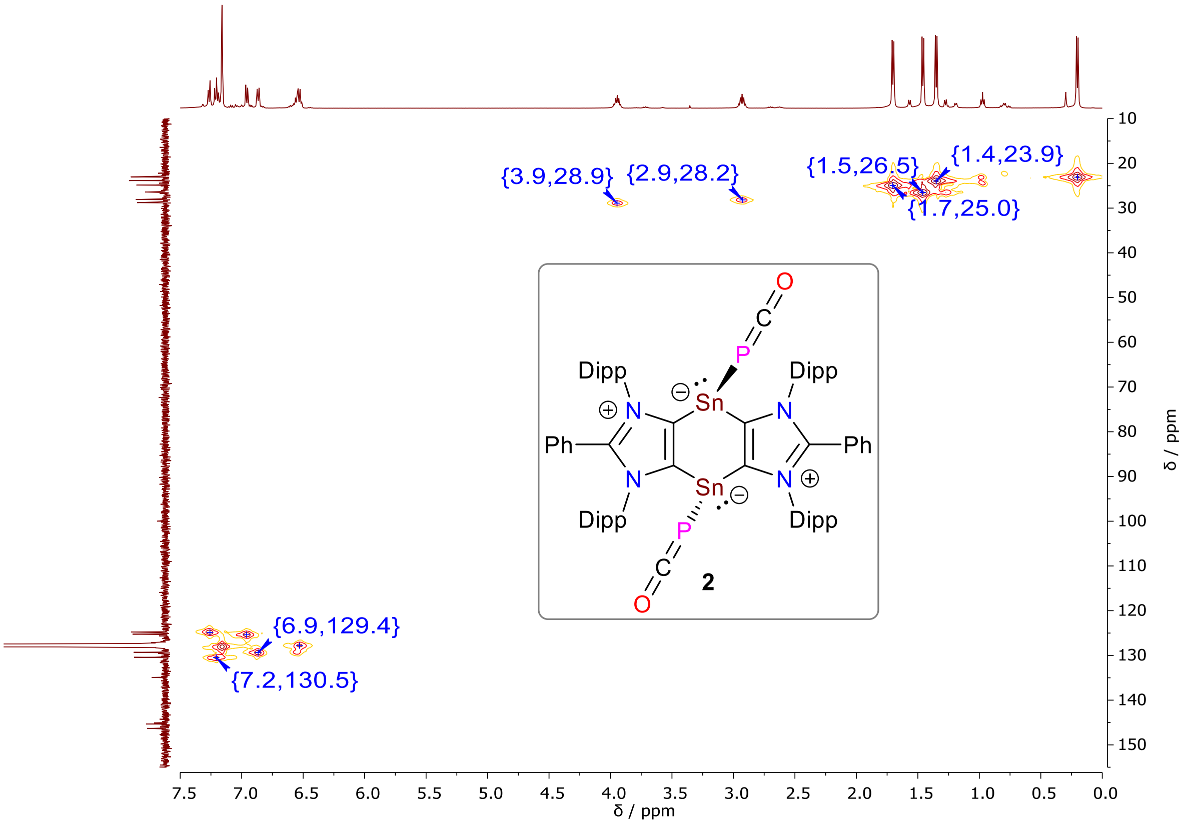

Figure S15. ^{1}H-^{13}C HMQC NMR (C_6D_6, 298 K) spectrum of compound **2**.

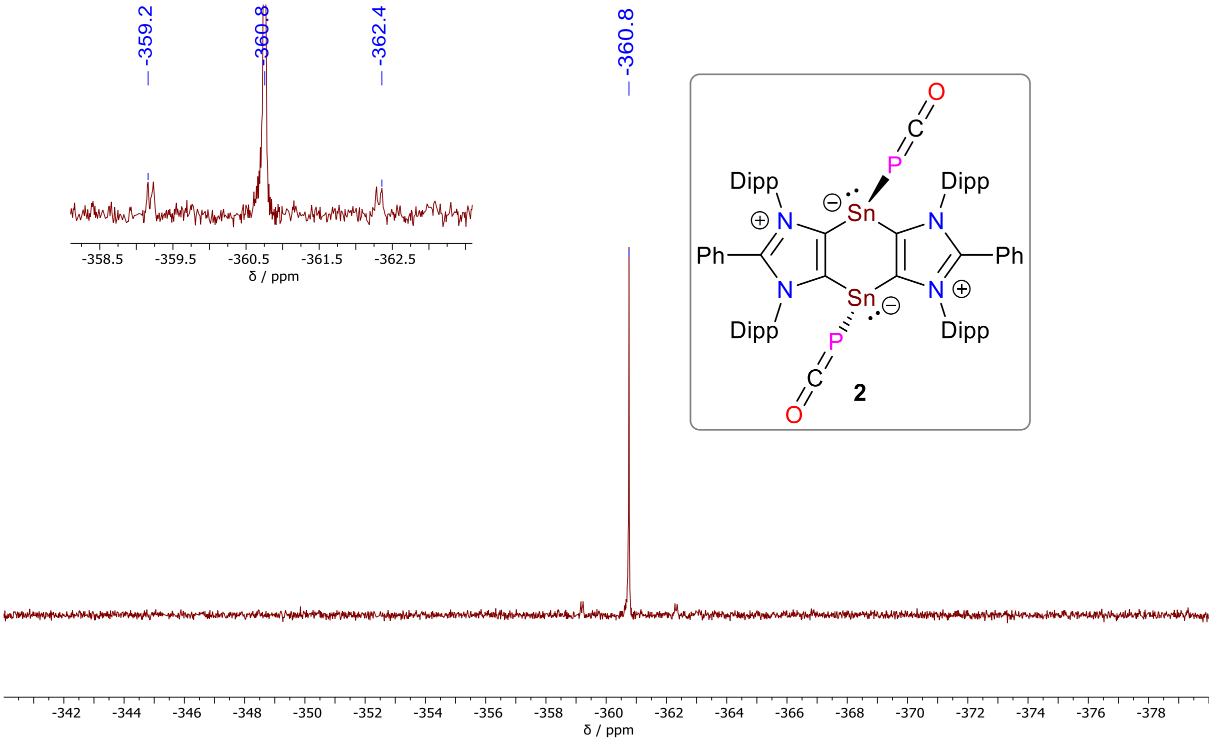

Figure S16. $^{31}P\{^{1}H\}$ NMR (202 MHz, C_6D_6, 298 K) spectrum of compound **2**.

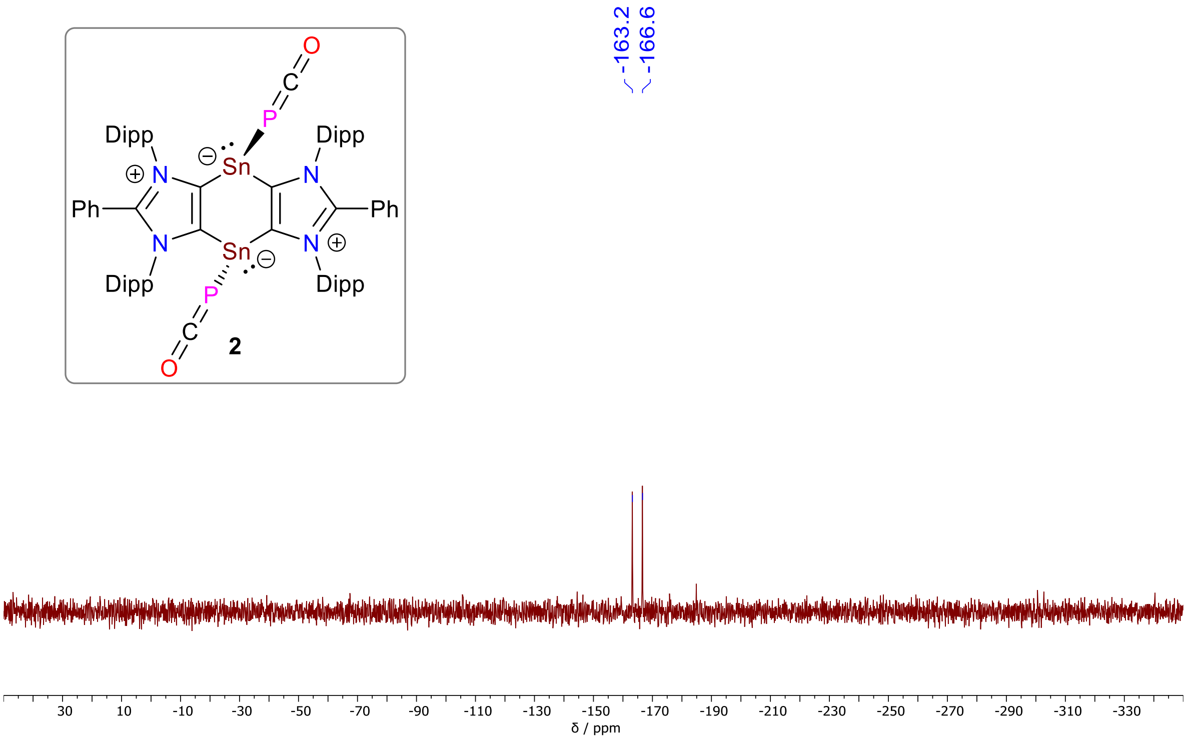

Figure S17. ^{119}Sn{^{1}H} NMR (187 MHz, THF-d_8, 298 K) spectrum of compound **2**.

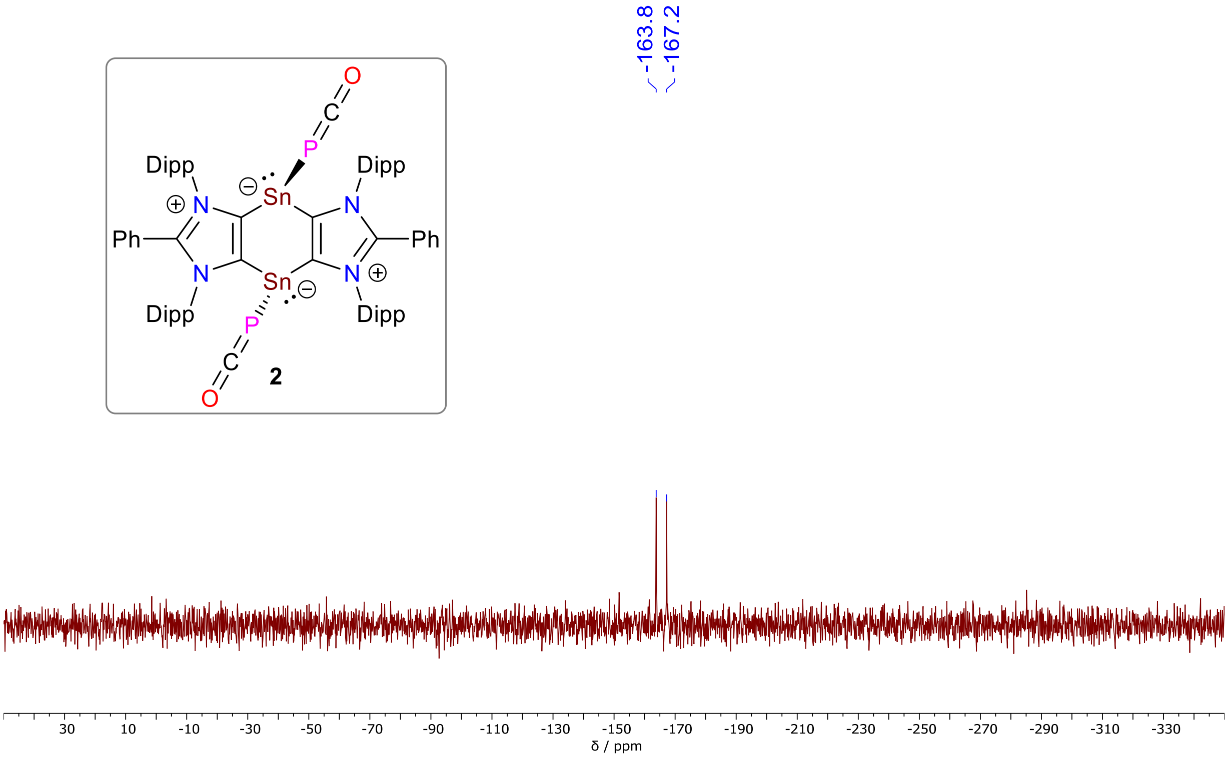

Figure S18. ^{119}Sn{^{1}H} NMR (187 MHz, C_6D_6, 298 K) spectrum of compound **2**.

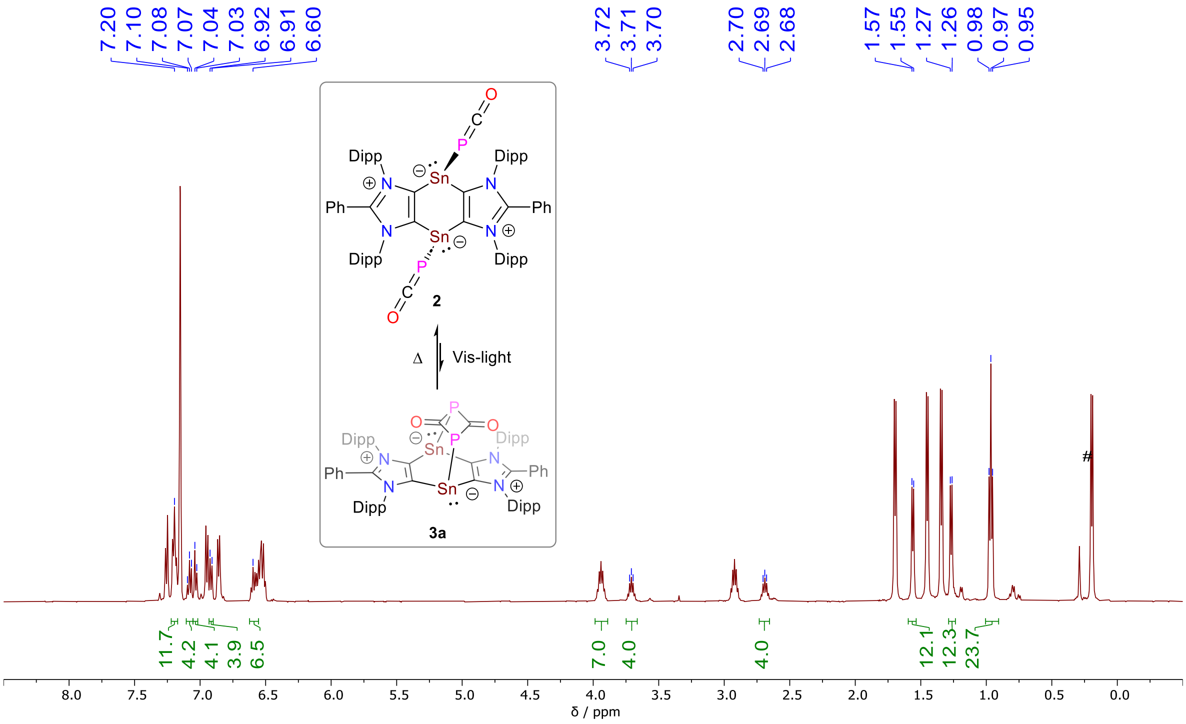

Figure S19. ^{1}H NMR (500 MHz, C_6D_6, 298 K) spectrum of a sample containing a mixture of **2** and **3a**.($^{\#}$grease)

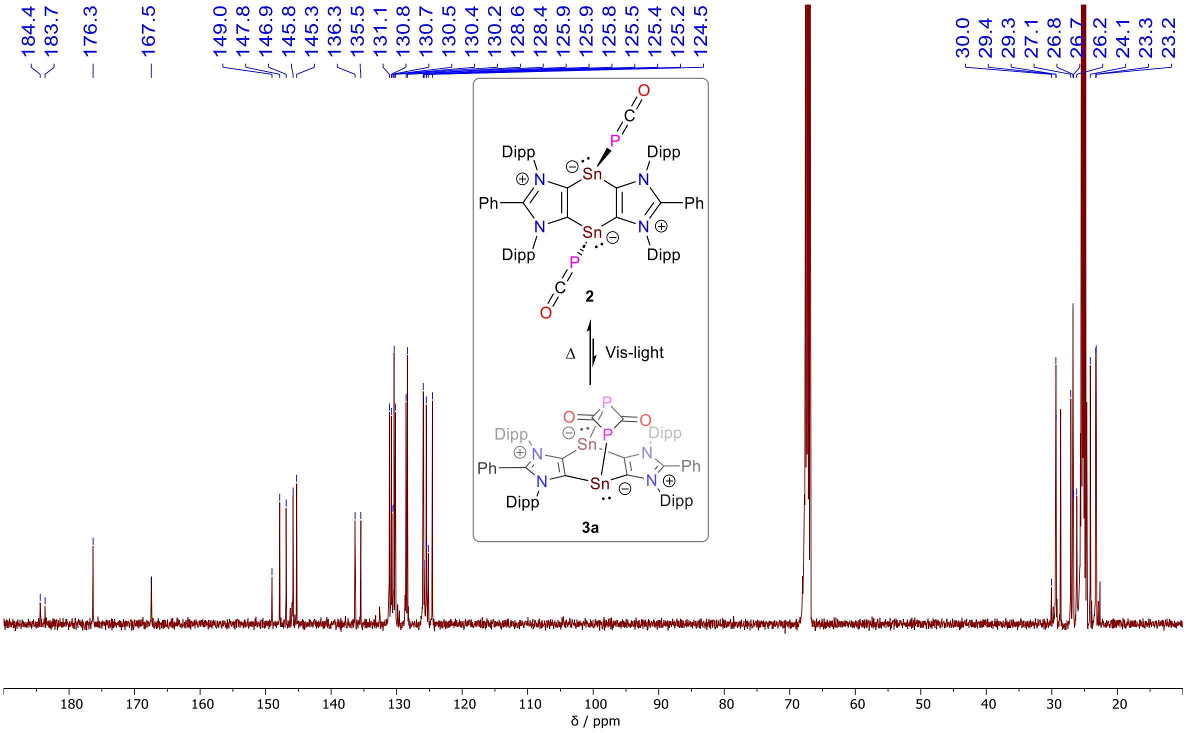

Figure S20. $^{13}C\{^{1}H\}$ NMR (126 MHz, THF-d_8, 298 K) spectrum of a sample containing a mixture of **2** and **3a**.

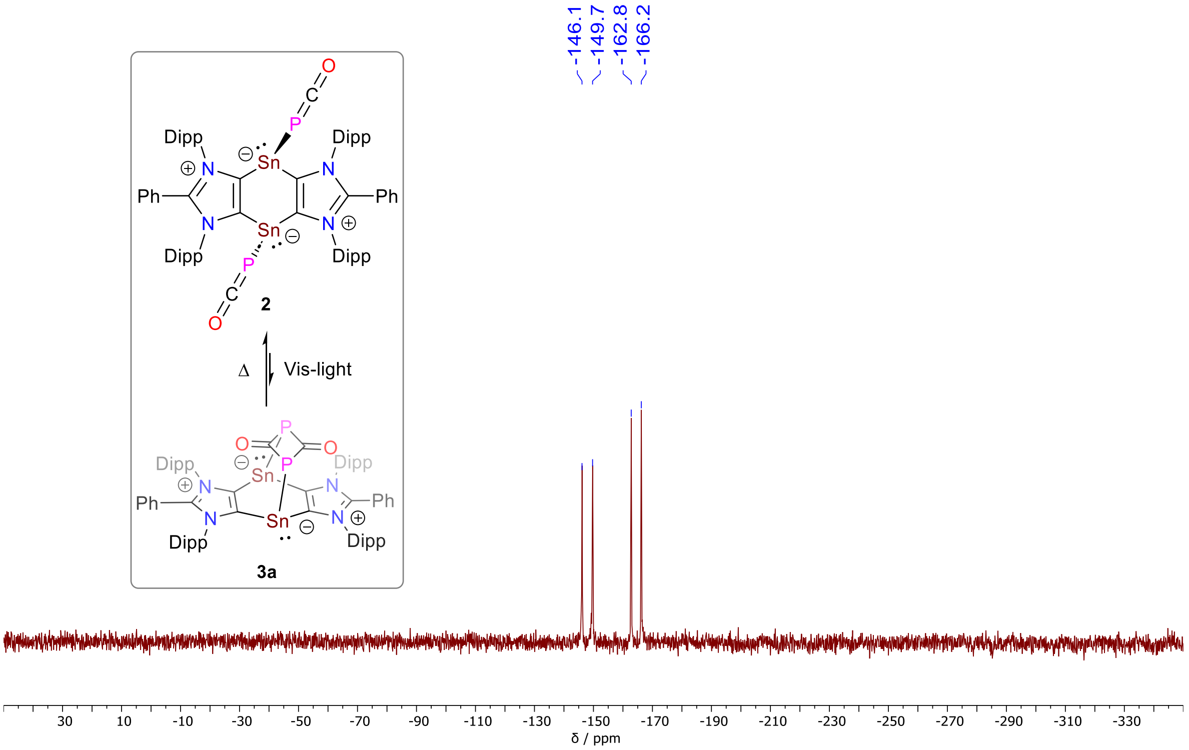

Figure S21. $^{119}Sn\{^{1}H\}$ NMR (187 MHz, THF-d_8, 298 K) spectrum of a sample containing a mixture of **2** and **3a**.

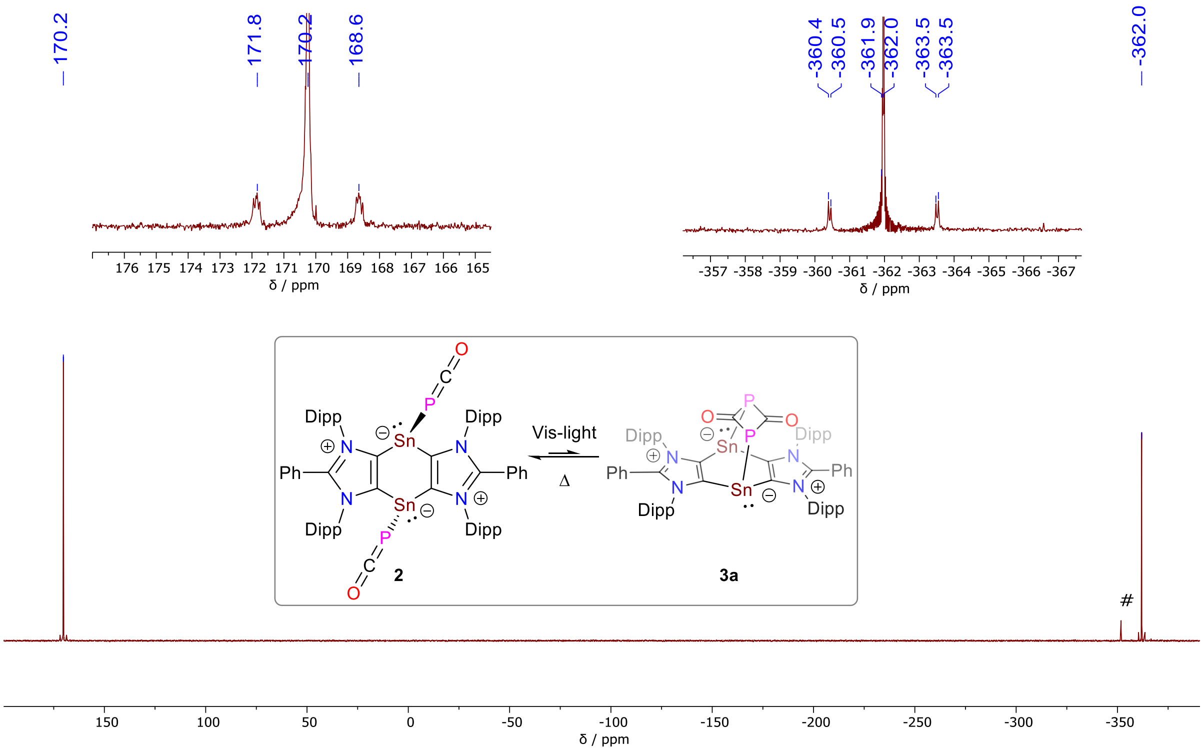

Figure S22. $^{31}P\{^{1}H\}$ NMR (202 MHz, THF-d_8, 298 K) spectrum of a sample containing a mixture of **2** and **3a**. ($^{\#}$(1,4-dioxane)$_{2.5}$NaPCO).

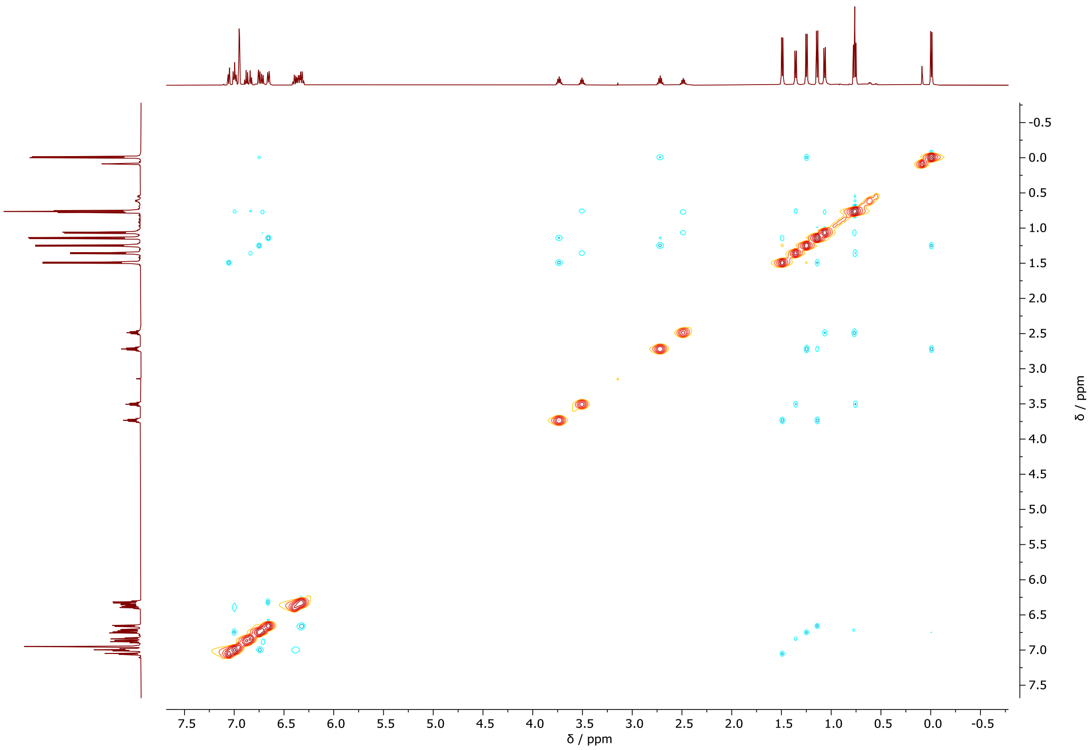

Figure S23. 2D ^{1}H EXSY NMR (500 MHz, C_6D_6, 298 K) spectrum of a sample containing a mixture of **2** and **3a**. Blue negative and red positive amplitude.

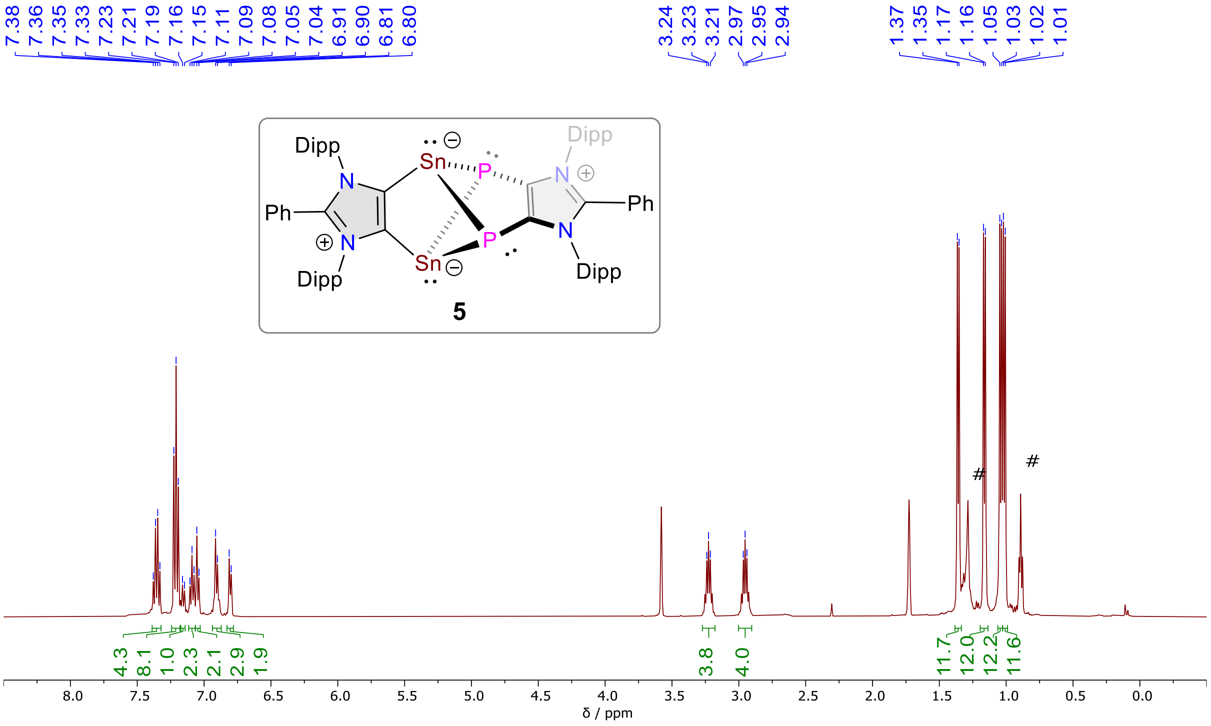

Figure S24. ^{1}H NMR (500 MHz, THF-d_8, 298 K) spectrum of compound **5**.(#*n*-hexane).

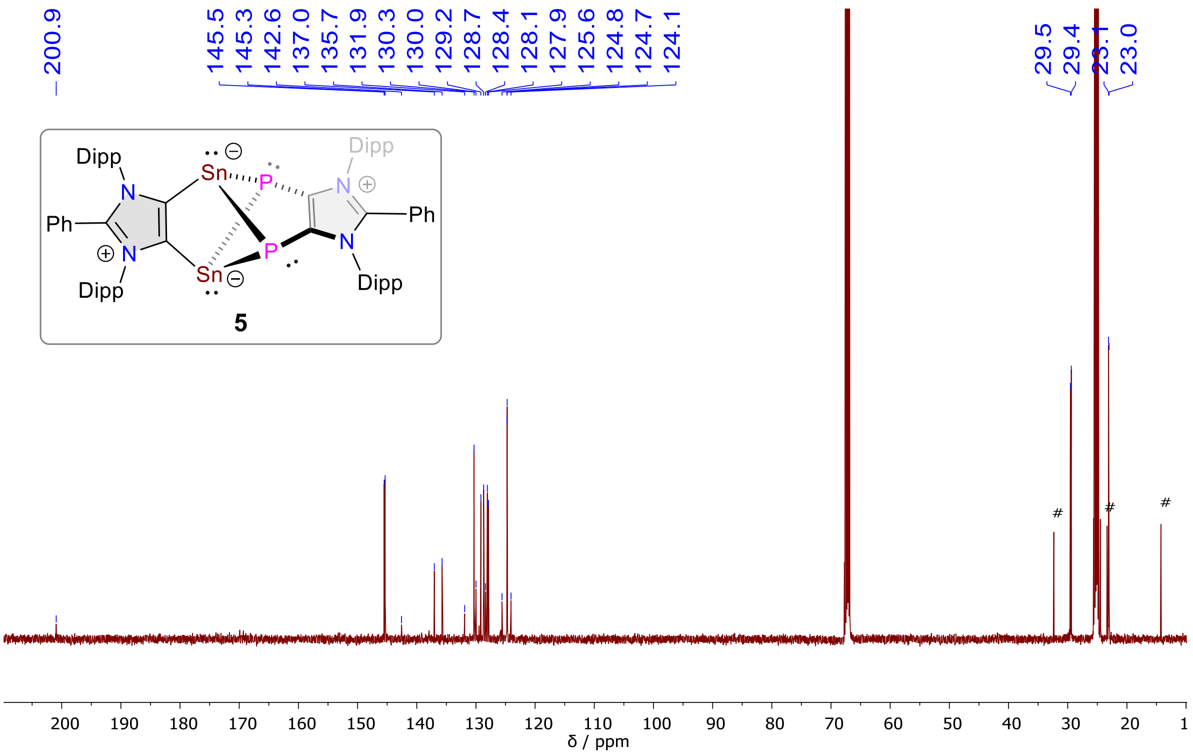

Figure S25. $^{13}C\{^{1}H\}$ NMR (126 MHz, THF-d_8, 298 K) spectrum of compound **5**.($^{\#}$*n*-hexane).

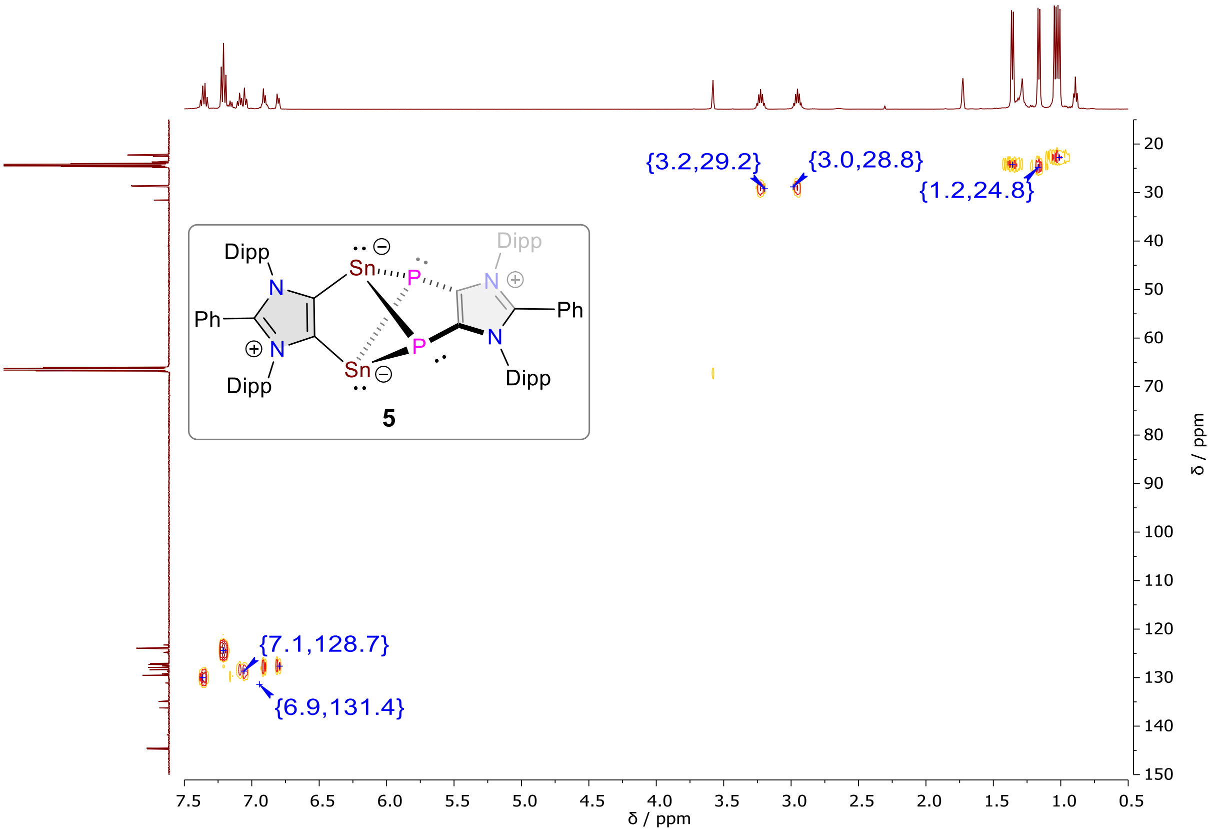

Figure S26. ^{1}H-^{13}C HMQC NMR (THF-d_8, 298 K) spectrum of compound **5**.($^{\#}$*n*-hexane).

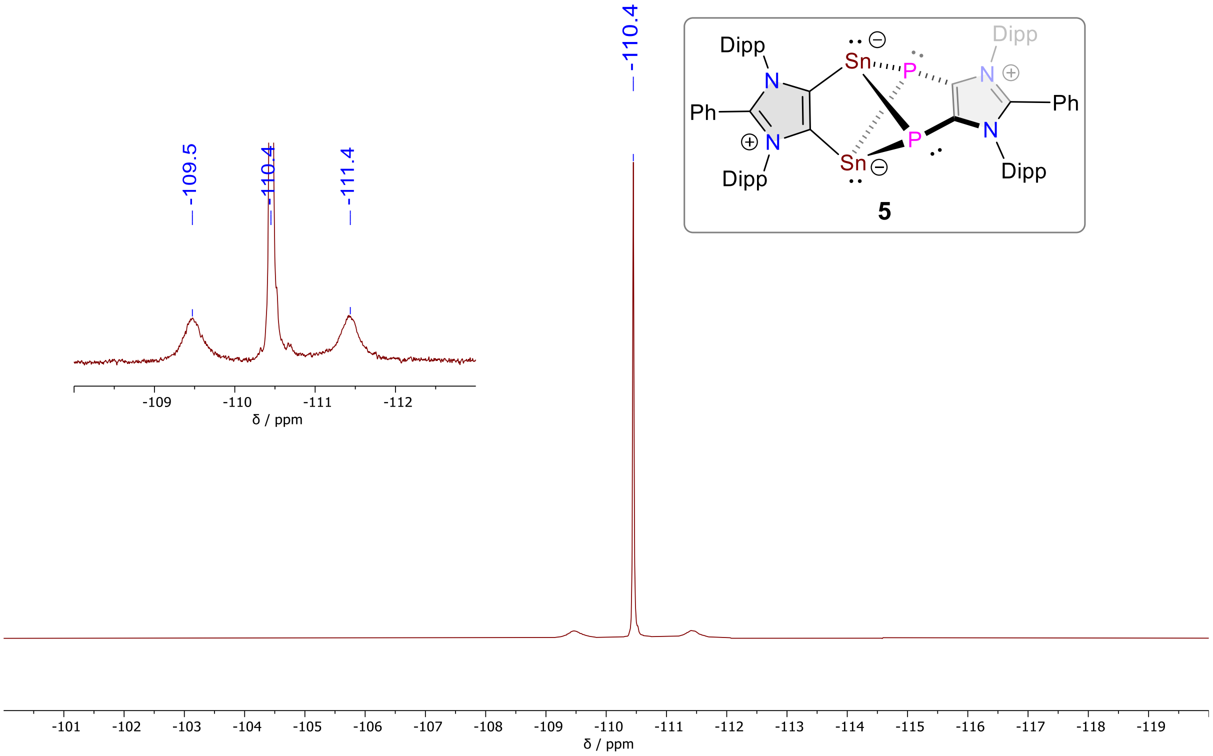

Figure S27. $^{31}P\{^{1}H\}$ NMR (202 MHz, THF-d_8, 298 K) spectrum of compound **5**.

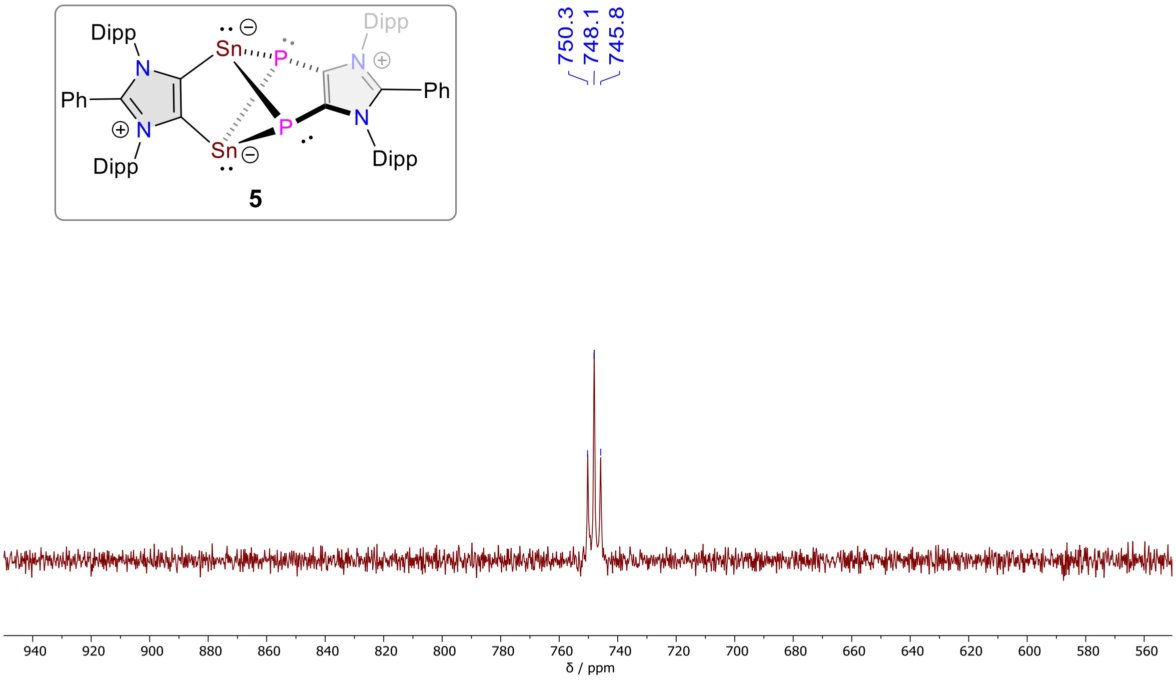

Figure S28. $^{119}Sn\{^{1}H\}$ NMR (187 MHz, THF-d_8, 298 K) spectrum of compound **5**.

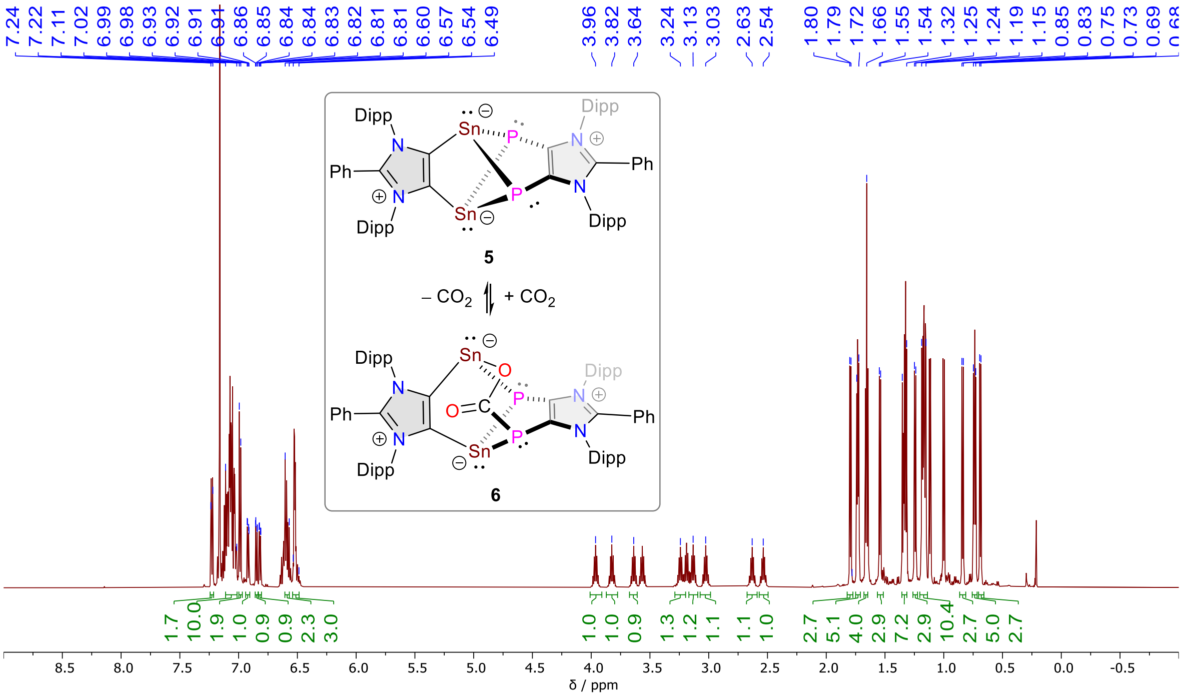

Figure S29. ^{1}H NMR (600 MHz, C_6D_6, 298 K) spectrum of a reaction of **5** with CO_2 (consist of a mixture of **5** and **6**).

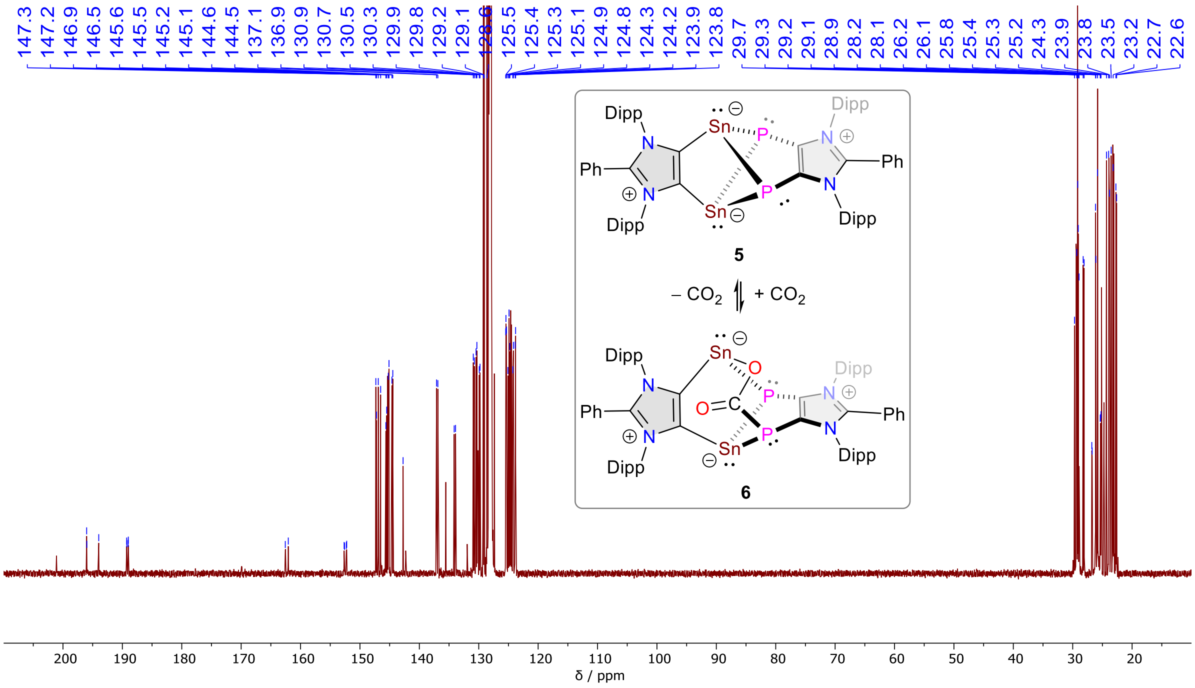

Figure S30. $^{13}C\{^{1}H\}$ NMR (151 MHz, C_6D_6, 298 K) spectrum of a reaction of **5** with CO_2 (consist of a mixture of **5** and **6**).

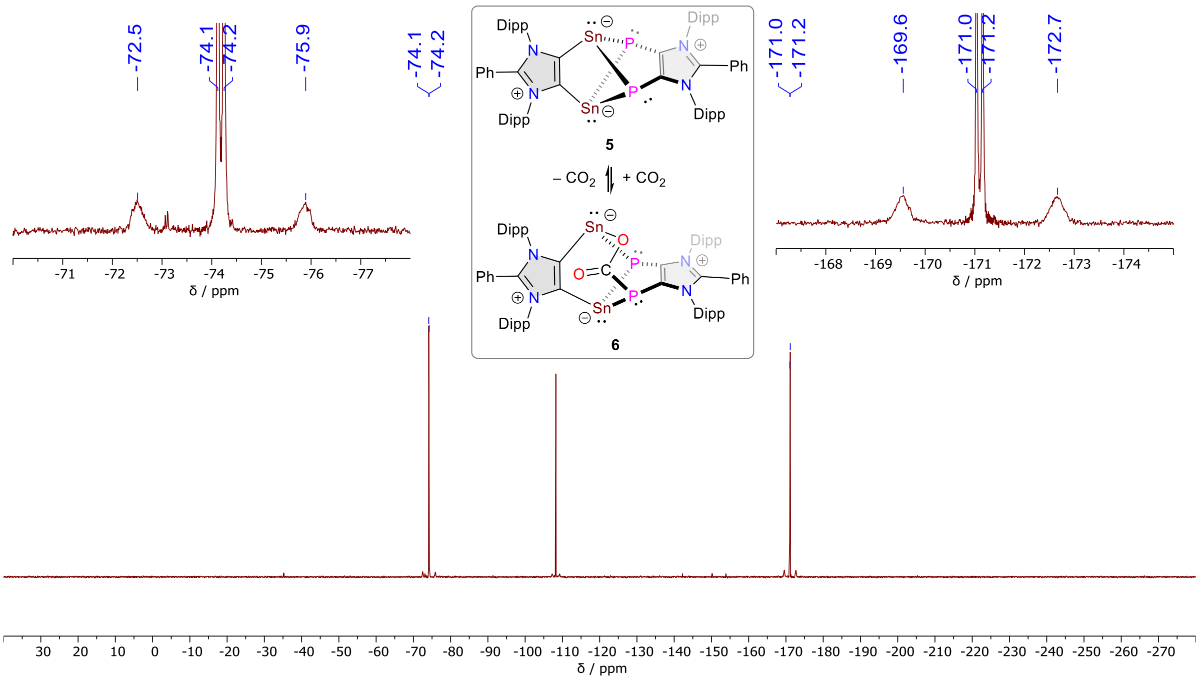

Figure S31. $^{31}P\{^1H\}$ NMR (202 MHz, THF-d_8, 298 K) spectrum of spectrum of a reaction of **5** with CO_2 (consist of a mixture of **5** and **6**).

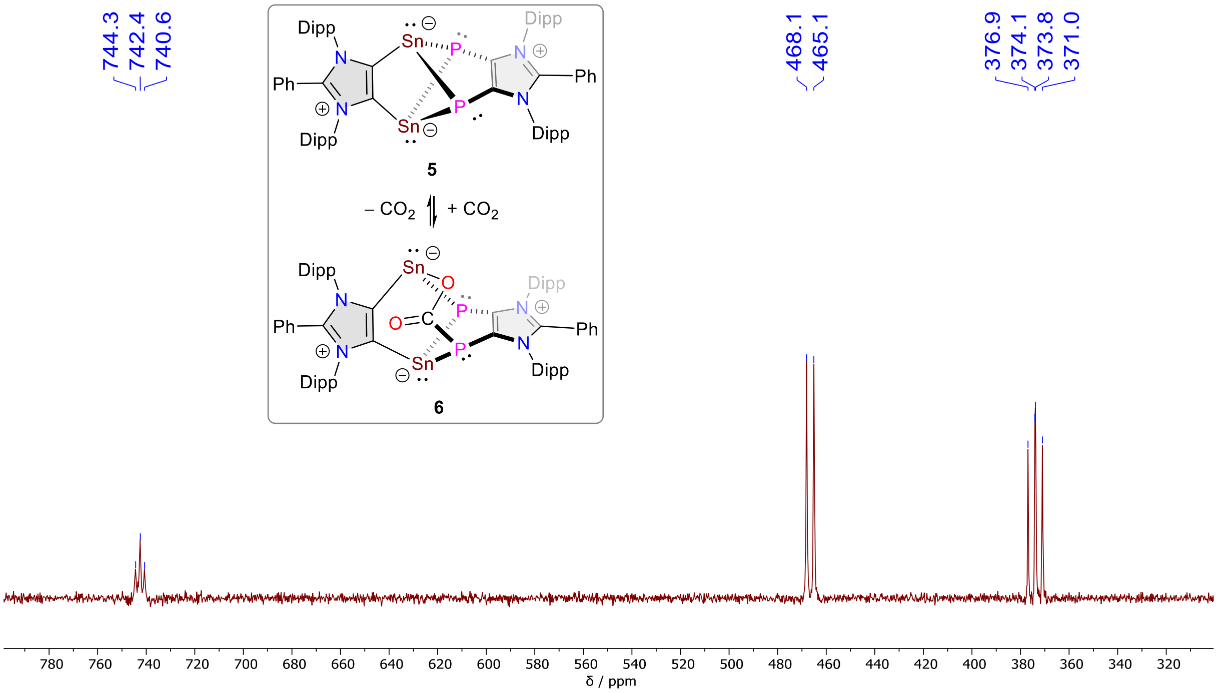

Figure S32. $^{119}Sn\{^1H\}$ NMR (224 MHz, C_6D_6, 298 K) spectrum of a reaction of **5** with CO_2 (consist of a mixture of **5** and **6**).

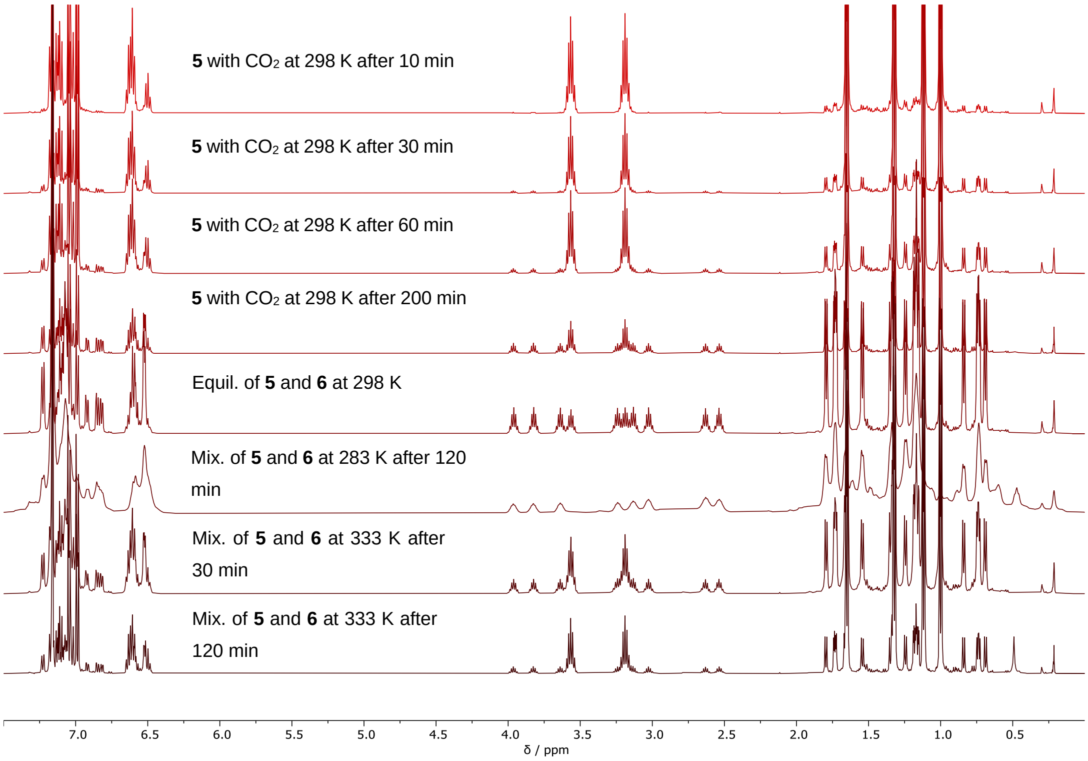

Figure S33. ^{1}H NMR (500 MHz, C_6D_6, 298 K) spectrum of a reaction of **5** with CO_2 (consist of a mixture of **5** and **6**) at room (298 K), lowered (283 K) and at elevated temperature (333 K).

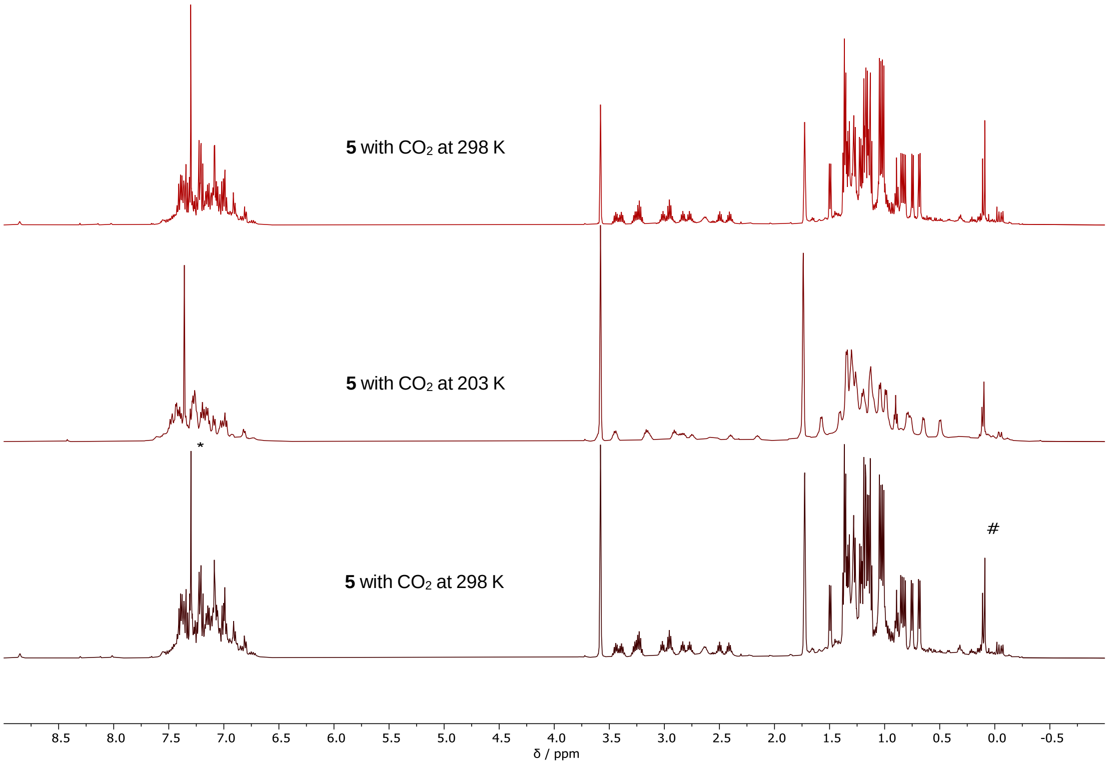

Figure S34. VT ^{1}H NMR (500 MHz, THF-d_8) spectra of compound **5** and **6**.([*]grease, [#]benzene).

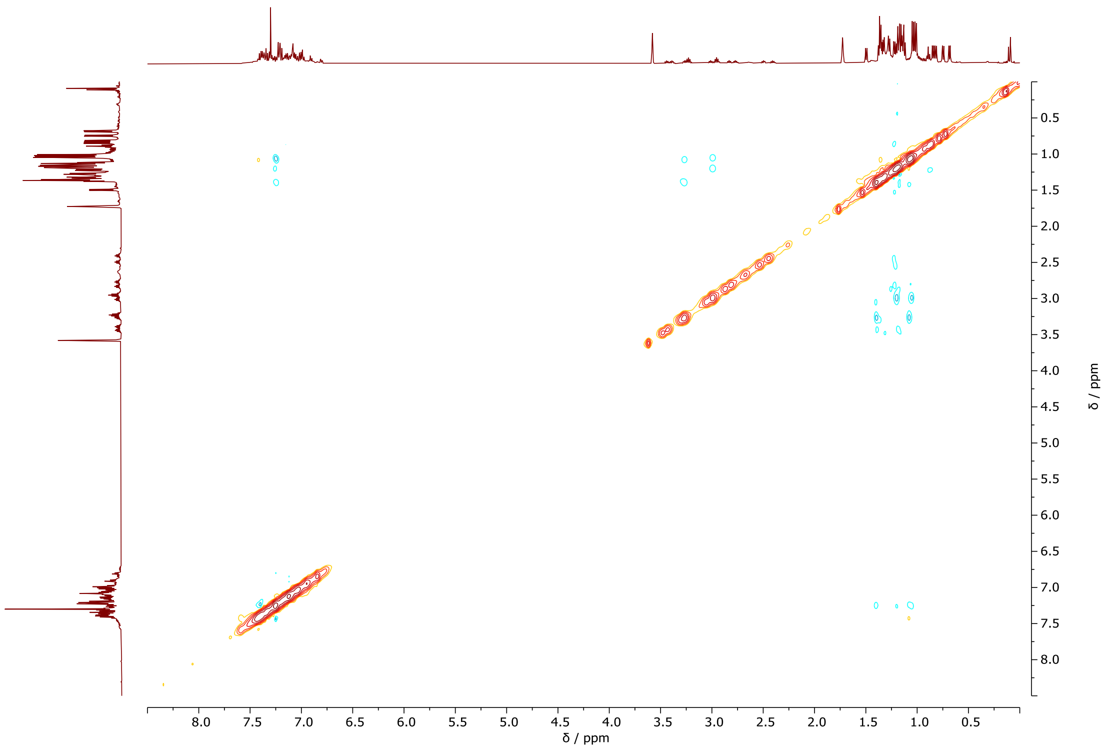

Figure S35. 2D ^{1}H EXSY NMR (500 MHz, THF-d_8, 298 K) spectrum of a sample containing a mixture of **5** and **6**. Blue negative and red positive amplitude.

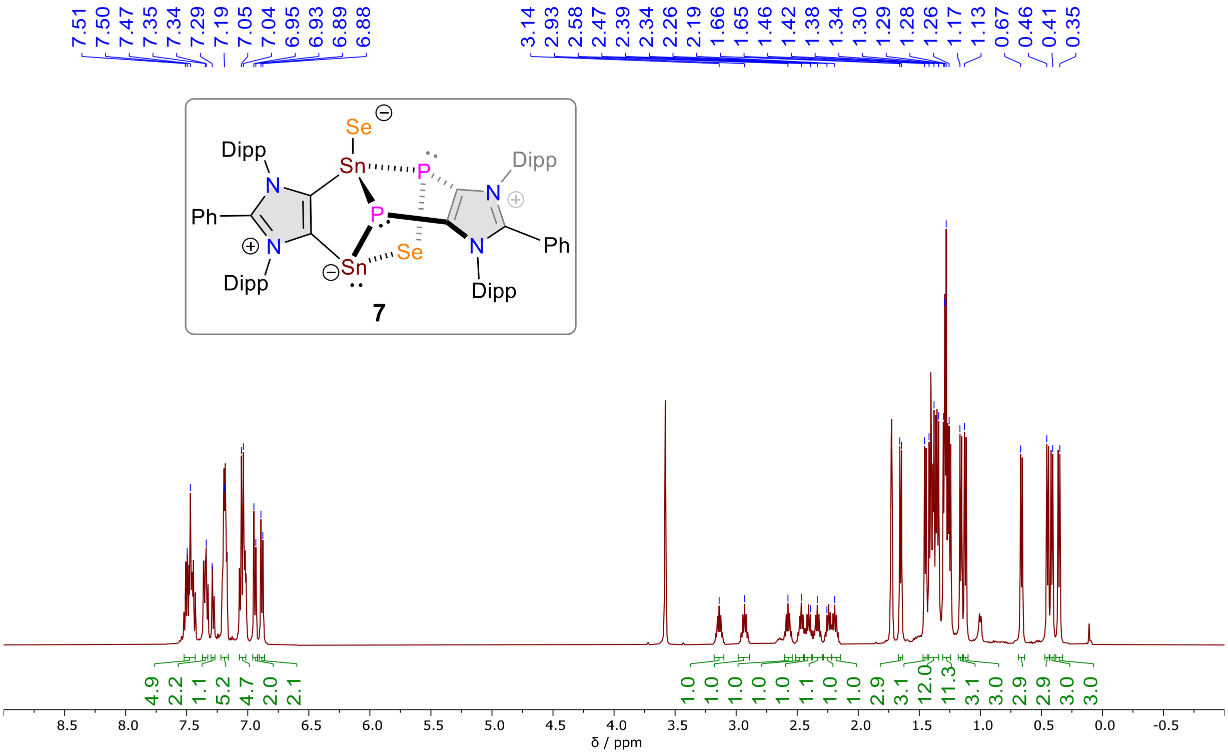

Figure S36. ^{1}H NMR (500 MHz, THF-d_8, 298 K) spectrum of compound **7**.

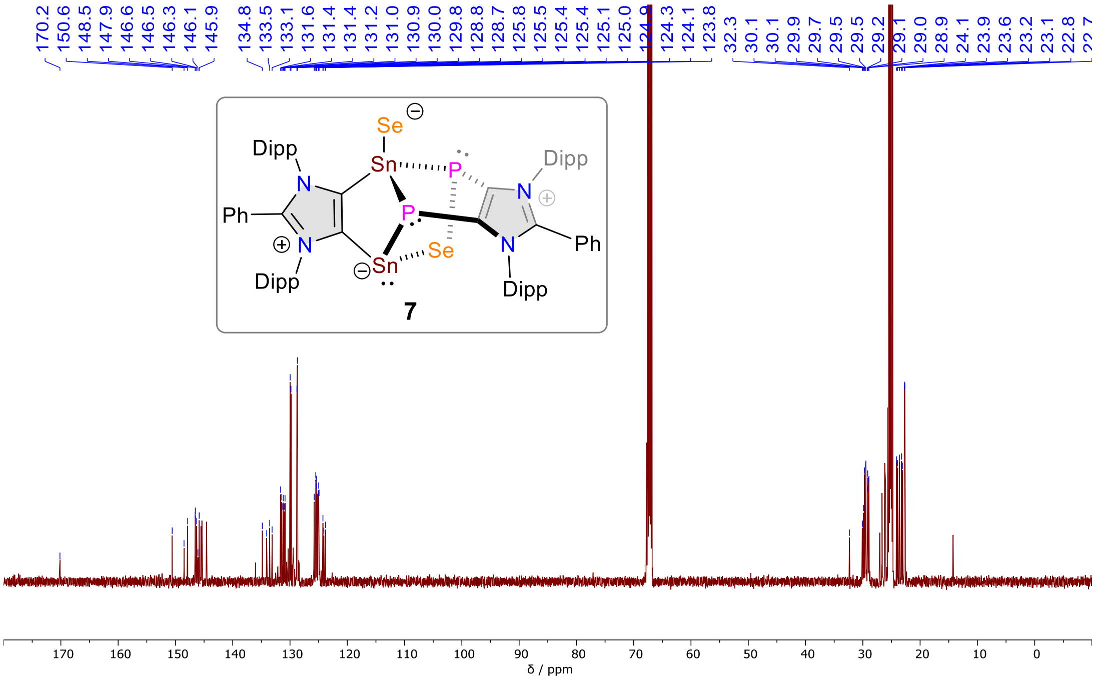

Figure S37. $^{13}C\{^{1}H\}$ NMR (126 MHz, THF-d_8, 298 K) spectrum of compound **7**.

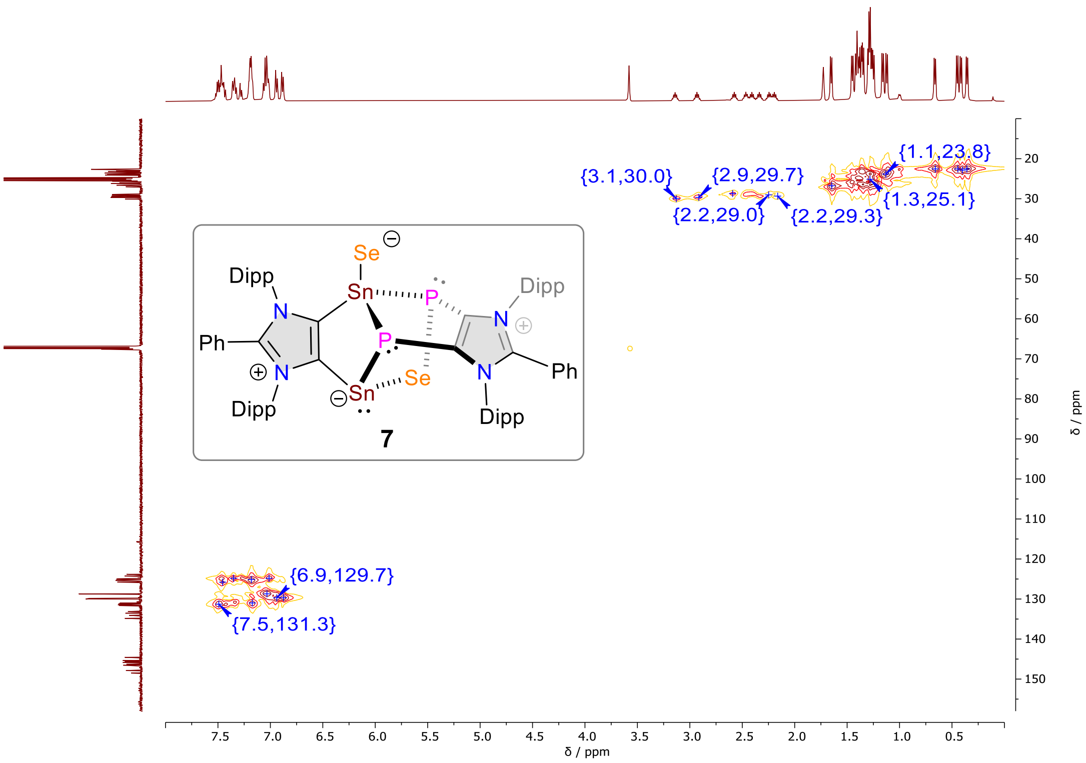

Figure S38. ^{1}H-^{13}C HMQC NMR (THF-d_8, 298 K) spectrum of compound **7**.

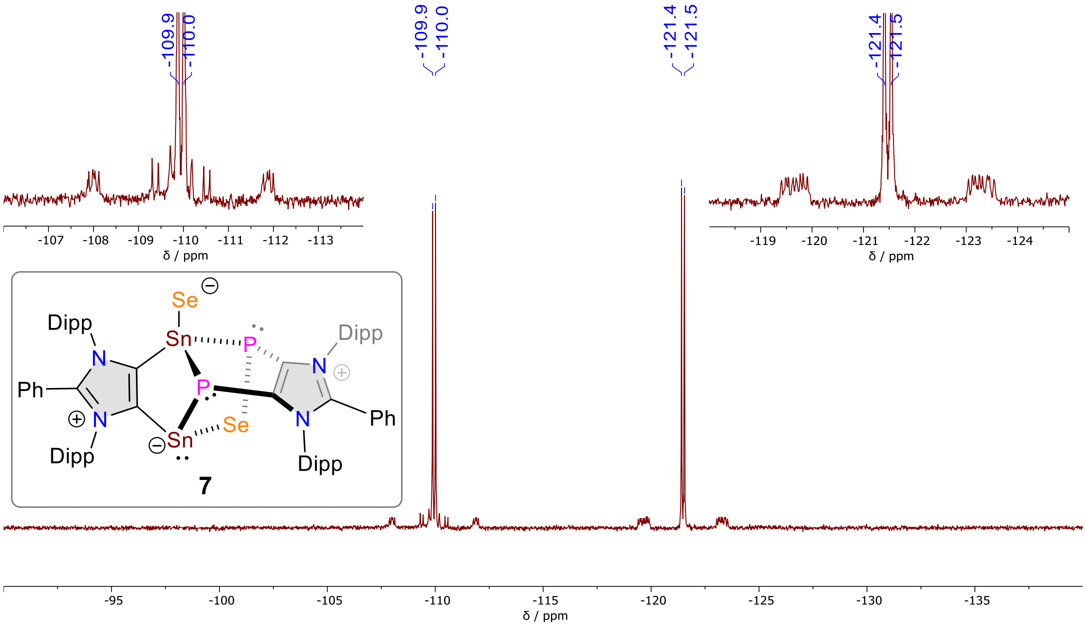

Figure S39. $^{31}P\{^1H\}$ NMR (202 MHz, THF-d_8, 298 K) spectrum of compound **7**.

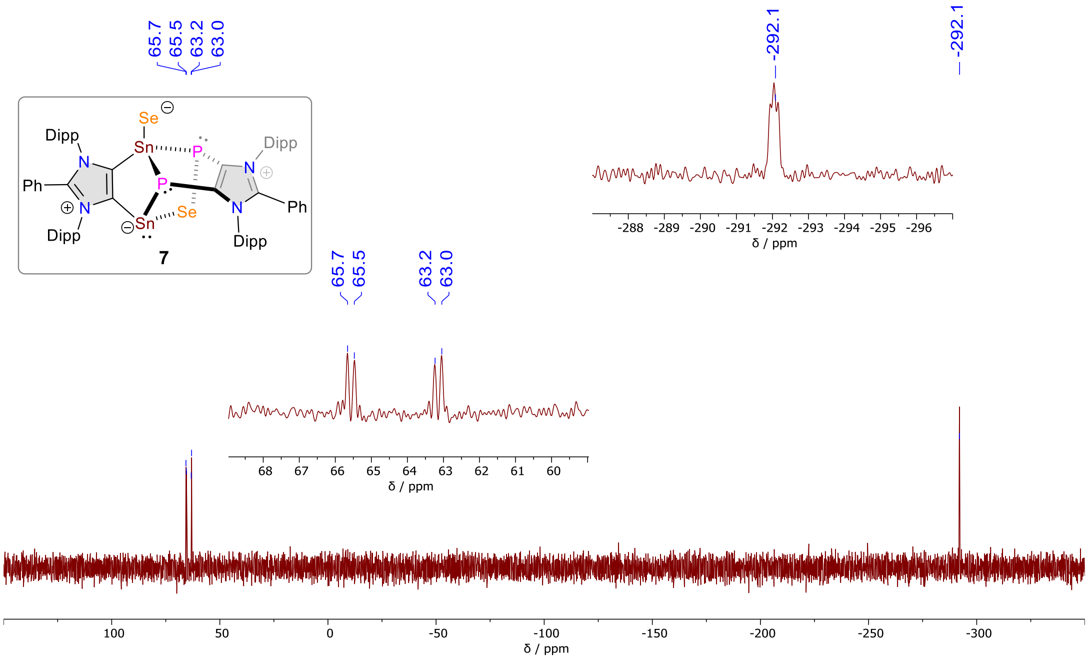

Figure S40. ^{77}Se NMR (95 MHz, THF-d_8, 298 K) spectrum of compound **7**.

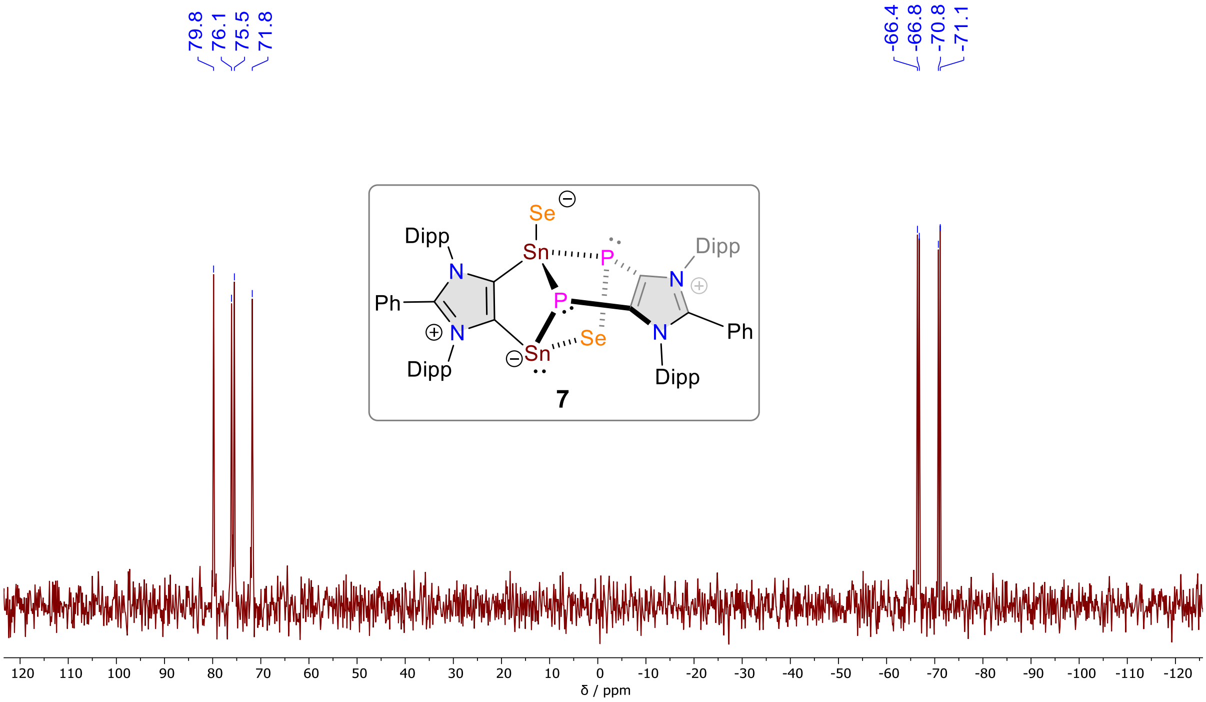

Figure S41. $^{119}Sn\{^{1}H\}$ NMR (187 MHz, THF-d_8, 298 K) spectrum of compound **7**.

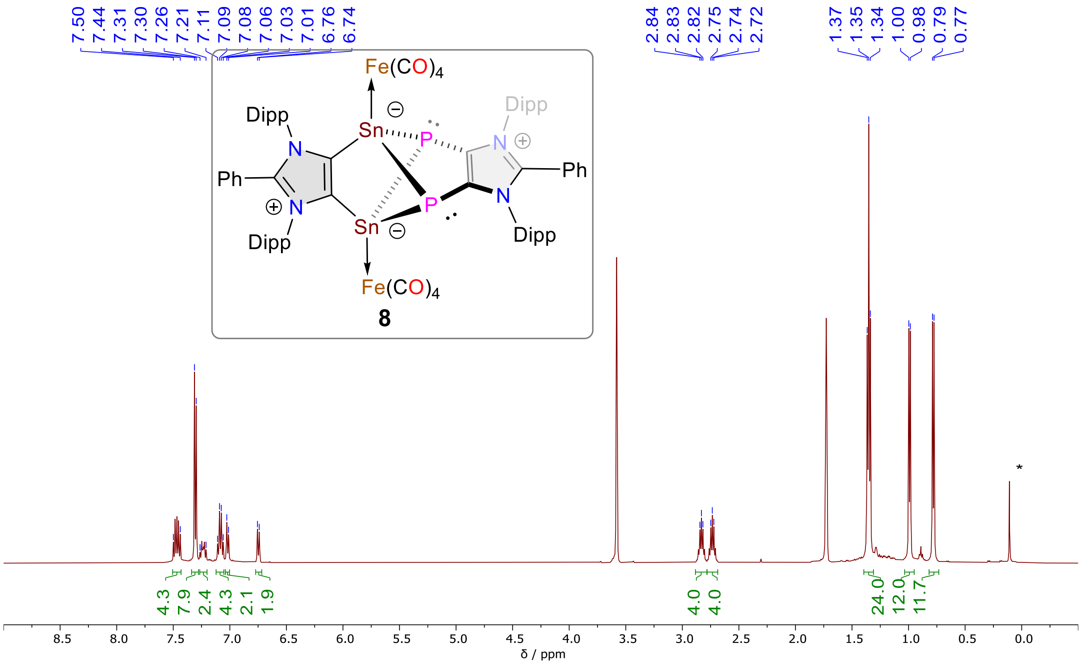

Figure S42. ^{1}H NMR (500 MHz, THF-d_8, 298 K) spectrum of compound **8**.(*grease).

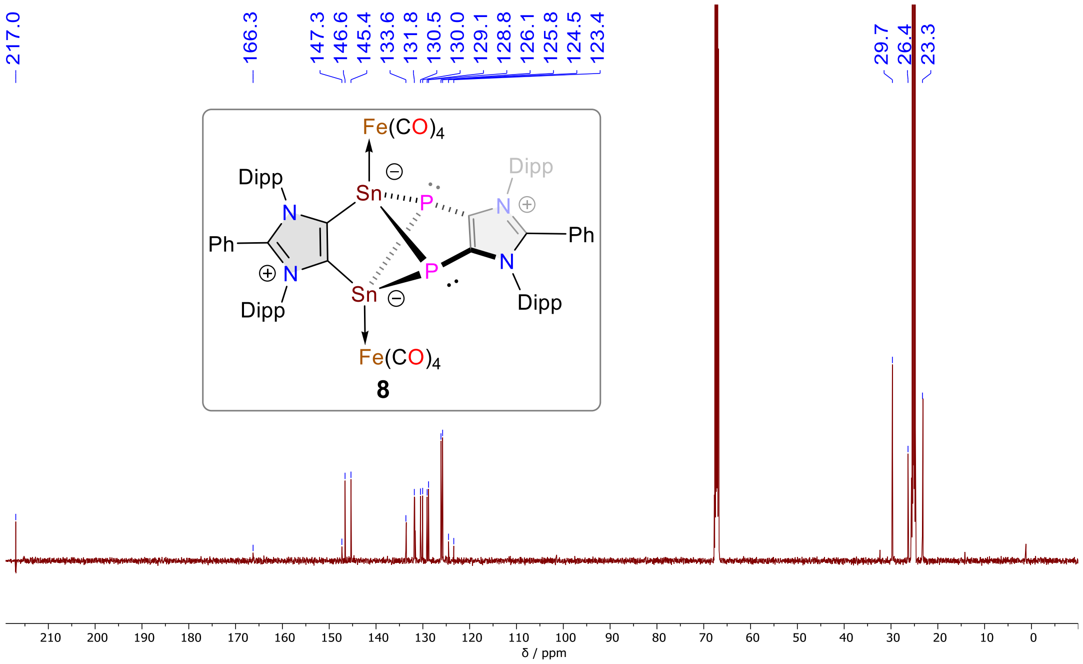

Figure S43. $^{13}C\{^{1}H\}$ NMR (126 MHz, THF-d_8, 298 K) spectrum of compound **8**.

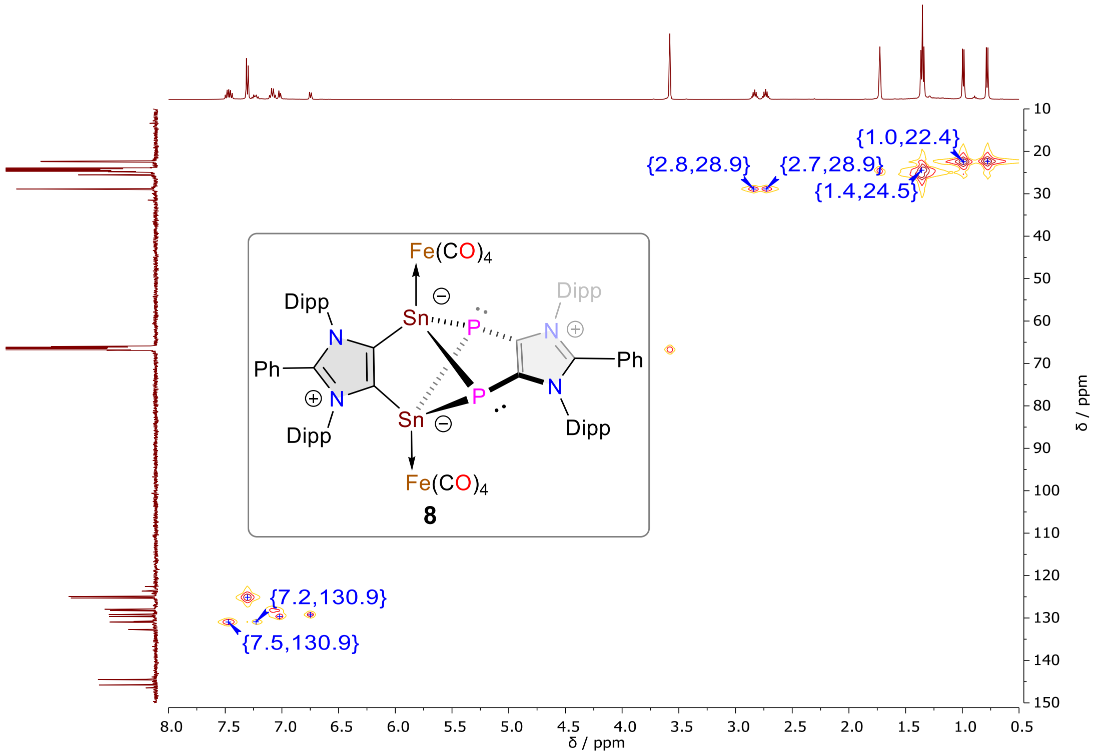

Figure S44. ^{1}H-^{13}C HMQC NMR (THF-d_8, 298 K) spectrum of compound **8**.

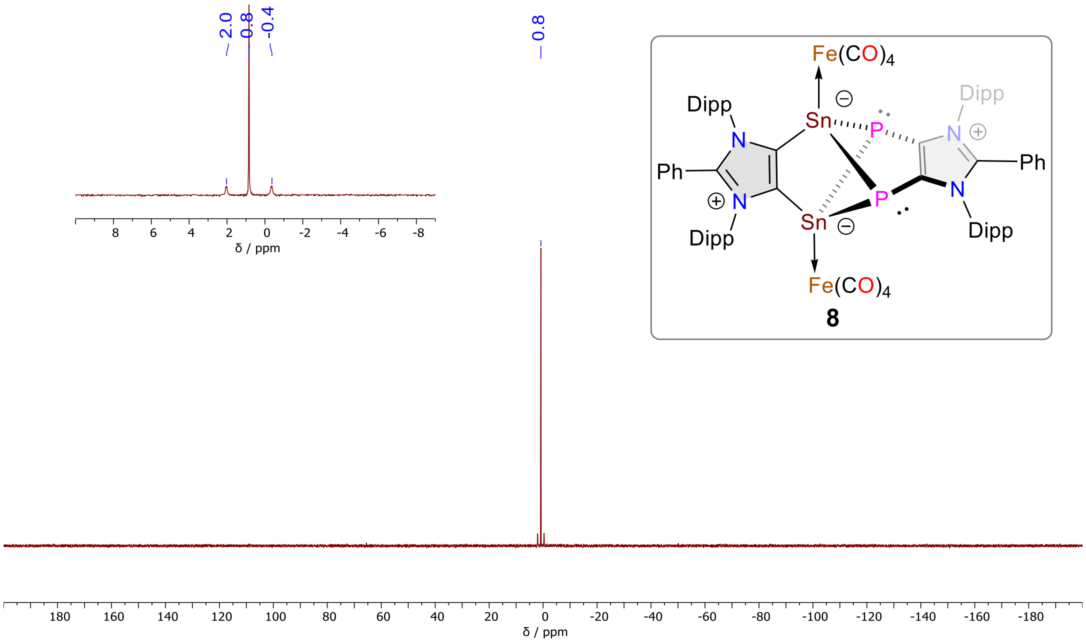

Figure S45. ^{31}P NMR (202 MHz, THF-d_8, 298 K) spectrum of compound **8**.

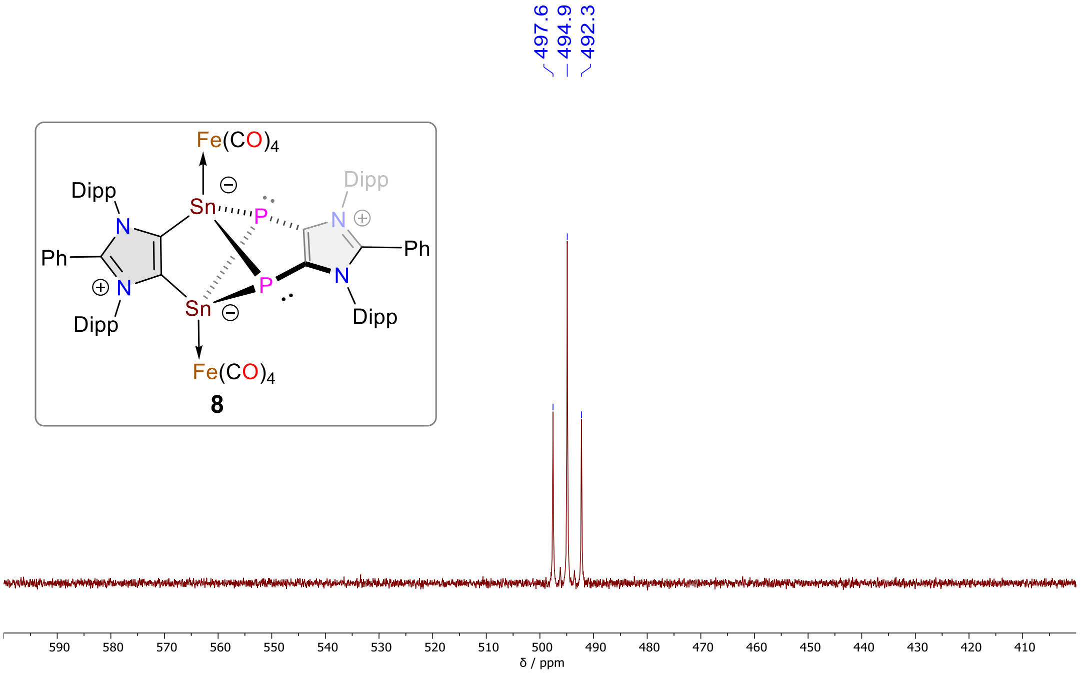

Figure S46. $^{119}Sn\{^1H\}$ NMR (187 MHz, THF-d_8, 298 K) spectrum of compound **8**.

UV-Vis Spectra

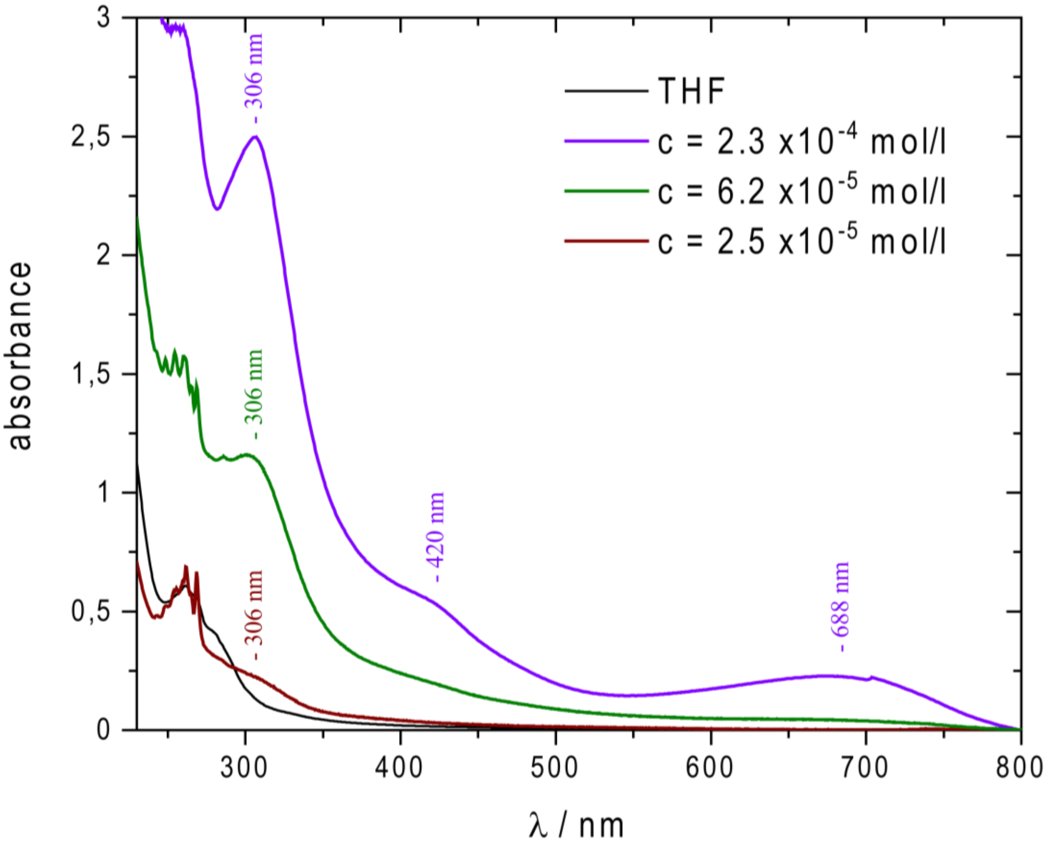

Figure S47. Stacked UV Vis spectra of **5** with different concentrations.

Infrared Spectra

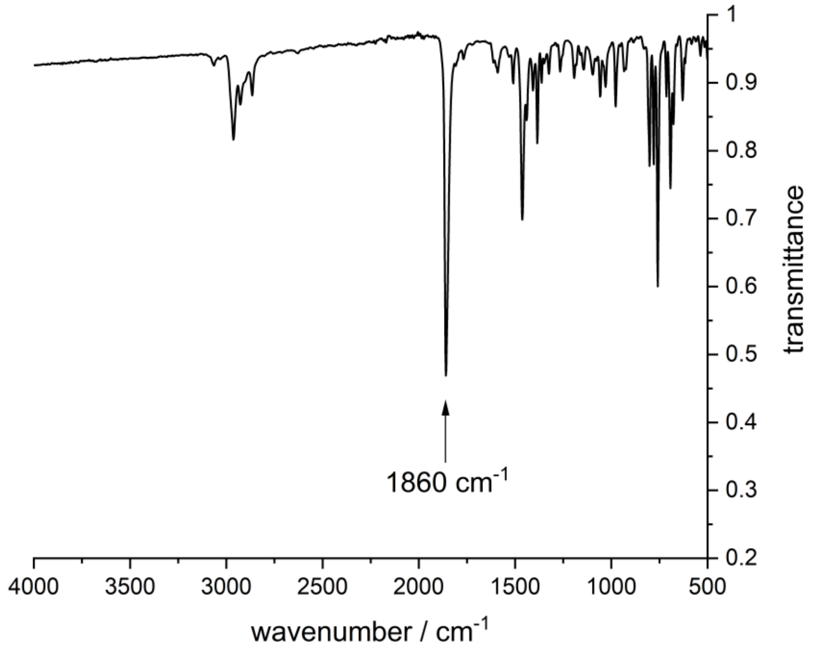

Figure S48. FTIR spectrum (neat) of **2**.

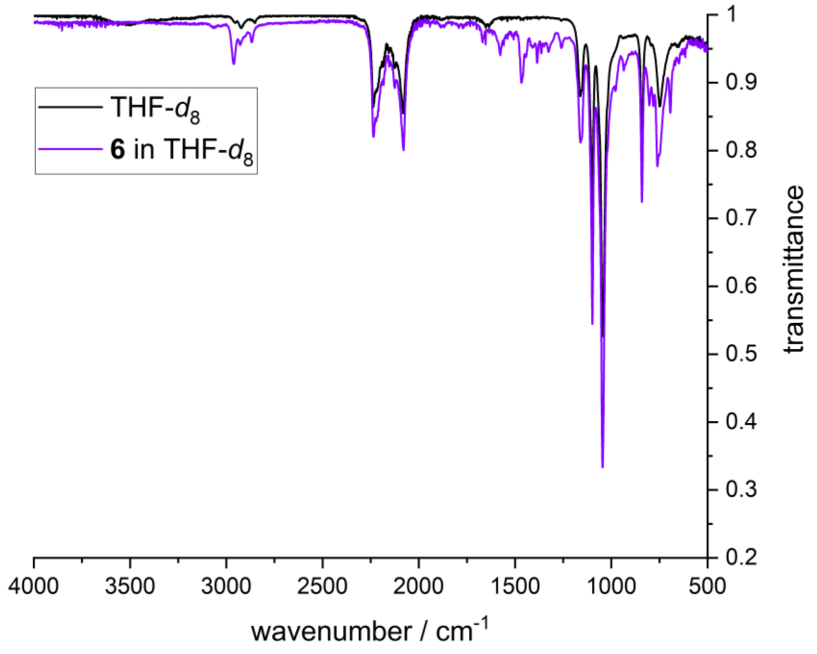

Figure S49. FTIR spectrum (in THF-d_8) of **6**.

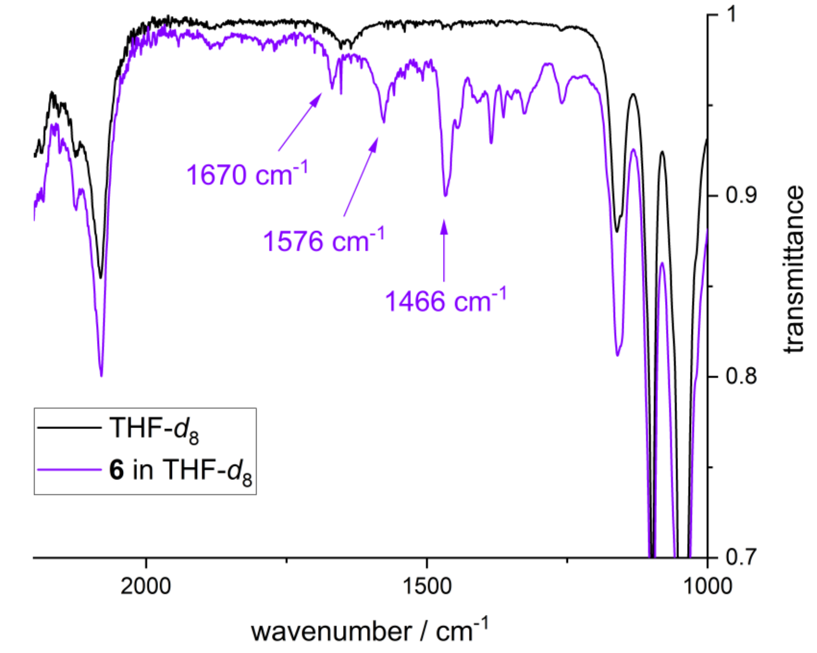

Figure S50. FTIR spectrum (in THF-d_8) of **6**.

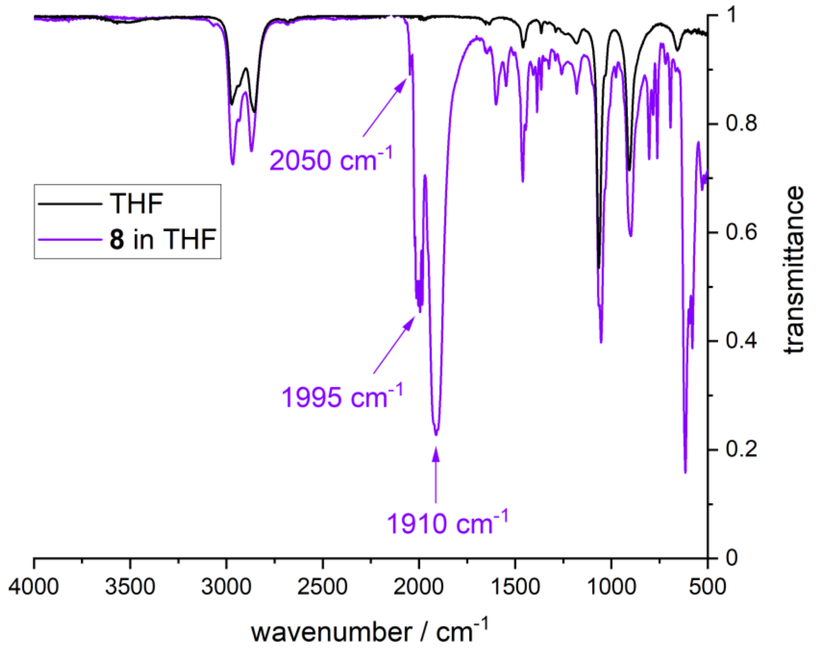

Figure S51. FTIR spectrum (in THF) of **8**.

Crystallographic Details

Single crystals were examined on a Rigaku Supernova diffractometer using Cu Kα (λ = 1.54184 Å) or Mo Kα (λ = 0.71073 Å) radiation. The crystal was kept at 100.00(10) K during data collection. Using Olex2,[7] the structure was solved with the ShelXT[8] structure solution program using Intrinsic Phasing and refined with the ShelXL[9] (**2**, **3a/3b**, **5**, **6**, and **8**) and olex2.refine[10] (**7**). The crystal of **2** contains heavily disordered THF solvent molecules, which could not be modelled satisfactorily. Therefore, a solvent mask was calculated, and 310 electrons were found in a volume of 1275 $Å^3$ in one void. This is consistent with the presence of 4 THF per formula unit which account for 320.0 electrons. For **3a**/3**b** disordered orientation of both $(PCO)_2$-ligands in ratio 70:30 and 17:83 was observed. Disorder of C29 to C33 over two sites in ratio 86:14. The lower occupied ring was refined as a regular hexagon, the displacement parameters were restrained with RIGU and SIMU. Disorder of C44, C45, and C46 over two sites in ratio 54:46. The displacement parameter of C44A and C44B were restrained with RIGU, SIMU and EADP. C48, C49, and C57 were disordered over two sites in ratio 51:49. Disorder of C61 over two sites in ratio 69:31. The displacement parameter of C62 to C67 were restrained with SIMU, due to large values and the impossibility to separate a disordered phenyl ligand. It was not possible to refine one highly disordered benzene reasonably, therefore a solvent mask was calculated and 198 electrons were found in a volume of 986 $Å^3$ in two voids per unit cell. This is nearly consistent with the presence of one benzene molecule per asymmetric unit which account for 168 electrons per unit cell. The crystal of **5** has a disorder of Sn/P position at a ratio of 69:31. Additionally, disorder of C37 to C48 over two sites (74:26), and disorder of C56 over two sites at a ratio 80:20. A solvent mask was calculated, and 19 electrons were found in a volume of 222 $Å^3$ in one void per unit cell. This is consistent with the presence of 0.5 benzene molecules per formula unit which account for 21.0 electrons. The sum formular includes this solvent for further calculations. Mixed crystal 89:11 of CO_2 adduct **6** and free **5** could be obtained. The crystal contains a disordered toluene molecule over two sites (78:22), suitable constraints and restraints were applied for this solvent molecule. For compound **7** the solvent pyridine is disordered at two positions, ratio 58:42. SIMU restraints were used for this disorder. The crystal of **8** contained one THF solvent molecule that is disordered with ratio 52:48, SIMU restraints were applied to all disordered carbon atoms. One additional THF solvent molecule is highly disordered, therefore a solvent mask was calculated and 156 electrons were found in a volume of 788 $Å^3$ in one void. This is consistent with the presence of one THF molecule per formula unit which account for 160.0 electrons.

Table S2. Crystallographic details of **2**, **3a**/**3b**, and **5**.

	$[(ADC)SnPCO]_2$ (**2**)) x 2 thf	$[(ADC)SnPCO]_2$ (**3a**/**3b**) x 2.5 benzene	$[(ADC)SnP]_2$ (**5**) x 2 benzene
Empirical formula	$C_{84}H_{110}N_4O_6P_2Sn_2$	$C_{98}H_{108}N_4O_2P_2Sn_2$	$C_{78}H_{90}N_4P_2Sn_2$
Formula weight	1571.07	1673.20	1382.85
Temperature/K	95.00(10)	100.00(10)	100.00(15)
Crystal system	triclinic	monoclinic	monoclinic
Space group	P-1	I2	$P2_1/c$
a/Å	14.4055(8)	28.9585(6)	17.8067(2)
b/Å	14.7480(7)	10.5653(2)	17.0832(3)
c/Å	18.9403(7)	28.7219(7)	23.8311(4)
α/°	88.781(4)	90	90
β/°	88.691(4)	92.300(2)	93.5780(10)
γ/°	85.613(4)	90	90
Volume/$Å^3$	4010.3(3)	8780.5(3)	7235.18(19)
Z	2	4	4
$\rho_{calc}g/cm^3$	1.301	1.266	1.270
μ/mm^{-1}	0.716	0.655	0.778
F(000)	1640.0	3480.0	2864.0
Crystal size/mm^3	0.35 × 0.261 × 0.149	0.4 × 0.08 × 0.07	0.38 × 0.34 × 0.05
Radiation/Å	Mo Kα (λ = 0.71073)	Mo Kα (λ = 0.71073)	Mo Kα (λ = 0.71073)
2Θ range for data collection/°	3.474 to 60.068	5.72 to 57.906	3.306 to 64.63
Index ranges	$-20 \le h \le 20$, $-20 \le k \le 20$, $-26 \le l \le 26$	$-36 \le h \le 38$, $-13 \le k \le 13$, $-39 \le l \le 38$	$-26 \le h \le 26$, $-25 \le k \le 25$, $-35 \le l \le 34$
Reflections collected	65576	76228	218594
Independent reflections	24743 [R_{int} = 0.0468, R_{sigma} = 0.0813]	19866 [R_{int} = 0.0604, R_{sigma} = 0.0529]	24471 [R_{int} = 0.0525, R_{sigma} = 0.0310]
Reflections with $I > 2\sigma(I)$	17513	17549	20496
Data/restraints/parameters	24743/0/720	19866/134/1047	24471/0/883
Goodness-of-fit on F^2	1.100	1.032	1.226
Final R indexes [$I > 2\sigma(I)$]	R_1 = 0.0839, wR_2 = 0.2026	R_1 = 0.0477, wR_2 = 0.1052	R_1 = 0.0686, wR_2 = 0.1492
Final R indexes [all data]	R_1 = 0.1117, wR_2 = 0.2163	R_1 = 0.0559, wR_2 = 0.1100	R_1 = 0.0815, wR_2 = 0.1537
Largest diff. peak/hole / e $Å^{-3}$	2.16/-1.84	1.27/-1.21	0.88/-1.18
Flack parameter		-0.018(8)	
CCDC number	2284045	2284046	2284047

Table S3. Crystallographic details of **6**, **7**, and **8**.

	[(ADC)$_2${SnOC(O)P}SnP] (**6**) x 2 toluene	[(ADC)$_2${Sn(Se)P}SnSeP]$_2$ (**7**) x pyridine x acn	[(ADC)SnP]$_2$[Fe(CO)$_4$]$_2$ (**8**) x 3 thf
Empirical formula	$C_{80.89}H_{94}N_4O_{1.77}P_2Sn_2$	$C_{73}H_{86}N_6P_2Se_2Sn_2$	$C_{86}H_{102}Fe_2N_4O_{11}P_2Sn_2$
Formula weight	1449.97	1504.823	1778.73
Temperature/K	100.00(10)	100.0(1)	100.0(1)
Crystal system	monoclinic	monoclinic	monoclinic
Space group	$P2_1/n$	$P2_1/c$	$P2_1/c$
a/Å	16.1913(3)	16.6805(5)	26.0390(2)
b/Å	19.0091(3)	21.8588(6)	15.84659(14)
c/Å	24.5120(5)	19.7261(6)	20.44725(16)
β/°	92.276(2)	104.185(3)	91.6449(7)
Volume/Å^3	7538.4(2)	6973.1(4)	8433.64(12)
Z	4	4	4
ρ_{calc}g/cm^3	1.278	1.433	1.401
μ/mm^{-1}	6.024	1.851	8.195
F(000)	3006.0	3053.2	3664.0
Crystal size/mm^3	0.1 × 0.08 × 0.03	0.168 × 0.118 × 0.059	0.156 × 0.13 × 0.088
Radiation/Å	Cu Kα (λ = 1.54184)	Mo Kα (λ = 0.71073)	Cu Kα (λ = 1.54184)
2Θ range for data collection/°	5.886 to 153.108	3.42 to 58.28	6.53 to 152.872
Index ranges	-20 ≤ h ≤ 14, -19 ≤ k ≤ 23, -25 ≤ l ≤ 30	-18 ≤ h ≤ 22, -28 ≤ k ≤ 16, -25 ≤ l ≤ 23	-32 ≤ h ≤ 32, -19 ≤ k ≤ 19, -25 ≤ l ≤ 25
Reflections collected	47980	33429	179649
Independent reflections	15216 [R_{int} = 0.0592, R_{sigma} = 0.0588]	15989 [R_{int} = 0.0312, R_{sigma} = 0.0506]	17630 [R_{int} = 0.0488, R_{sigma} = 0.0229]
Reflections with $I > 2\sigma(I)$	12463	13032	15880
Data/restraints/parameters	15216/244/884	15989/186/846	17630/75/963
Goodness-of-fit on F^2	1.014	1.046	0.979
Final R indexes [$I > 2\sigma(I)$]	R_1 = 0.0587, wR_2 = 0.1474	R_1 = 0.0420, wR_2 = 0.0971	R_1 = 0.0337, wR_2 = 0.0744
Final R indexes [all data]	R_1 = 0.0712, wR_2 = 0.1556	R_1 = 0.0570, wR_2 = 0.1044	R_1 = 0.0384, wR_2 = 0.0769
Largest diff. peak/hole / e Å^{-3}	1.24/-1.56	2.55/-0.90	0.57/-0.76
CCDC number	2284048	2284049	2284050

Molecular Structures

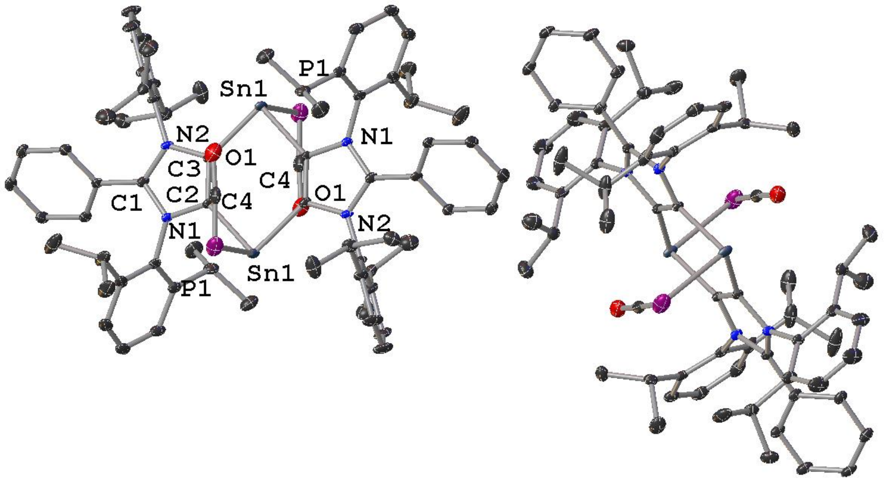

Figure S52. Molecular structure of **2**. H atoms are omitted for clarity. Thermal ellipsoids are shown with 50% probability. Selected bond lengths (Å) and angles (°): C2–C3, 1.377(7); C3–Sn1, 2.198(5); Sn1–P1, 2.677(2); P1–C4, 1.503(8); C4–O1, 1.280(9); C2–Sn1–P1, 95.1(1); C4–P1–Sn1, 92.2(3); C2–C3–Sn1, 133.1(4); O1–C4–P1, 176.1(6).

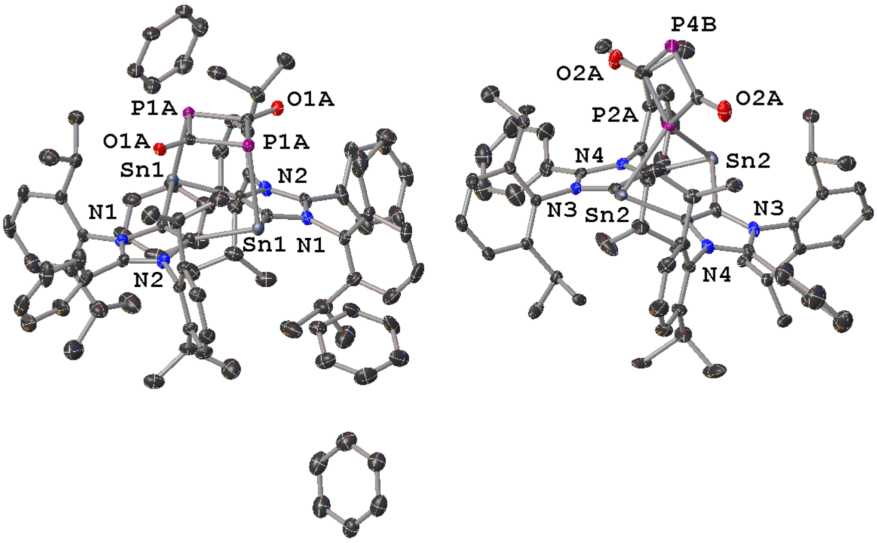

Figure S53. Molecular structure of **3a** and **3b**. H atoms are omitted for clarity only disorder part 1 is shown. Thermal ellipsoids are shown with 50% probability. Selected bond lengths (Å) and angles (°): **3a**: C2–C3, 1.359(8); C2–Sn1, 2.225(6); Sn1–P1A, 2.711(2); P1A–C34A, 1.862(9); C34A–O1A, 1.249(11); C2–Sn1–P1A, 94.5(2); C34A–P1A–Sn1, 100.2(3); C2–C3–Sn1, 129.6(4); O1A–C34A–P1A, 130.4(7); C34A–P1A–C34A^1, 80.2(4); P1A–C34A–P1A1, 99.8(4). **3b**: C36–C37, 1.361(8); C36–Sn2, 2.240(7); Sn2–P2A, 2.697(2); P2A–C68A, 1.885(9); P4B–C68A, 1.807(9); C68A–O2A, 1.212(9); C36–Sn2–P2A, 77.1(2); C68A–P2A–Sn2, 120.3(2); C36–C37–Sn2, 125.8(5); O2A–C68A–P2A, 128.3(7); P4B–C68A–P2A, 98.1(4); C68A–P4B–C68A^2, 84.0(5).

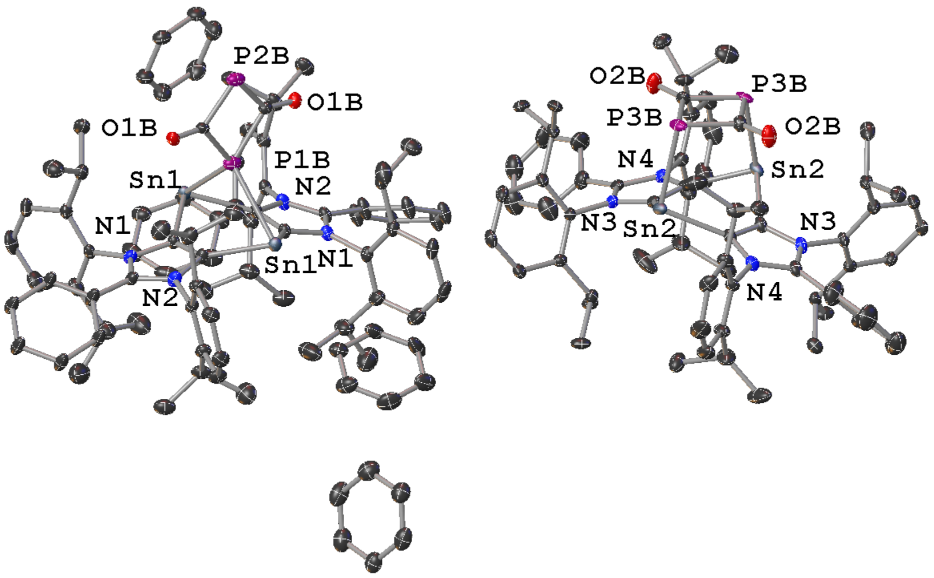

Figure S54. Molecular structure of **3a** and **3b**. H atoms are omitted for clarity only disorder part 2 is shown. Thermal ellipsoids are shown with 50% probability.

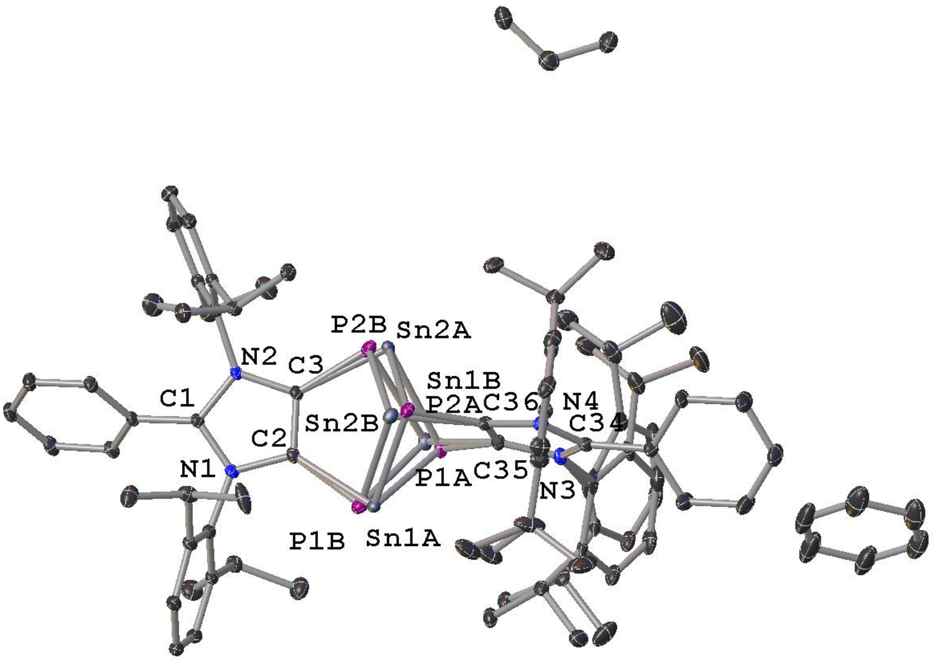

Figure S55. Molecular structure of **5**. H atoms are omitted for clarity. Thermal ellipsoids are shown with 50% probability. Selected bond lengths (Å) and angles (°): C2–C3, 1.372(4); C35–C36, 1.378(4); Sn1A–C2, 2.218(3); Sn2A–C3, 2.245(3); Sn1A–P1A, 2.643(3); Sn1A–P2A, 2.672(4); Sn2A–P1A, 2.695(3); Sn2A–P2A, 2.647(5); P1A–C35, 1.822(4); P2A–C36, 1.853(5); C2–Sn1A–P2A, 90.5(1); C2–Sn1A–P1A 88.6(1); Sn1A–P1A–C35, 86.1(1); Sn1A–P1A–Sn2A, 83.7(1); P1A–Sn1A–P2A, 80.9(1); Sn1A–C2–C3, 119.6(2); P1A–C35–C36, 123.3(3).

Plane angle between the two C_3N_2–motifs 77.3°

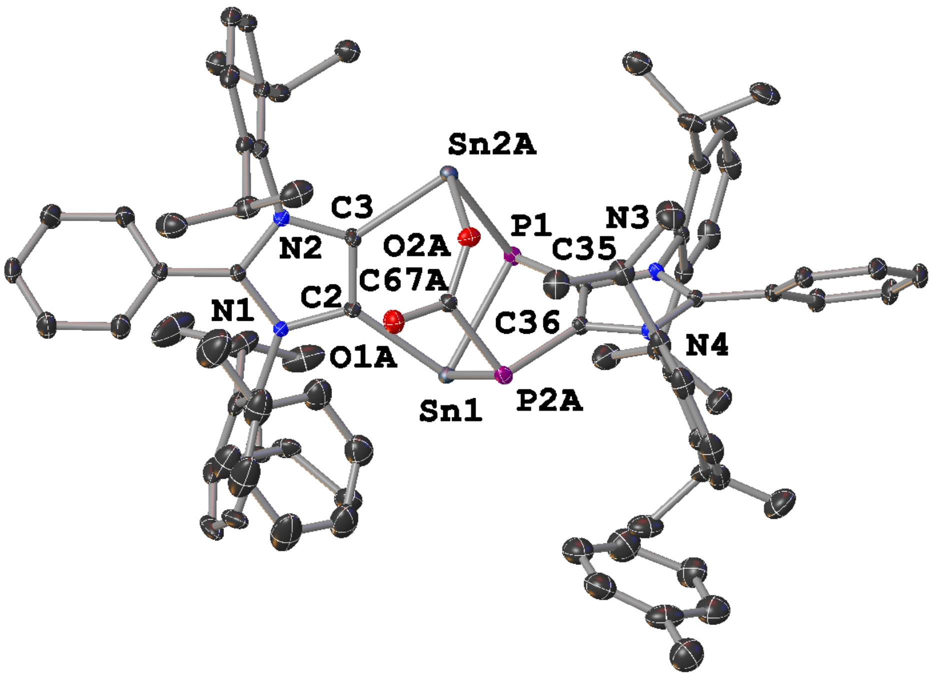

Figure S56. Molecular structure of **6**. H atoms are omitted for clarity. Mixed crystal of **5** and **6** (89/11) where only **6** is shown. Thermal ellipsoids are shown with 50% probability. Selected bond lengths (Å) and angles (°): C2–C3, 1.391(6); C2–Sn1, 2.214(4); C3–Sn2A, 2.231(5); O2A–C67, 1.297(7); P2A–C67A, 1.853(6); P2A–C36, 1.847(5); P1–C35, 1.814(5); Sn1–P1, 2.595(1); Sn1–P2A, 2.672(1); Sn2A–P1, 2.611(1); C3–C2–Sn1, 126.1(3); C2–C3–Sn2A, 125.5(3); C35–P1–Sn1, 89.3(2); C35–P1–Sn2A, 97.7(1); P1–Sn1–P2A, 87.9(1); C36–P2A–C67A, 101.9(2); O1A–C67A–P2A, 115.5(4); O1A–C67A–O2A, 125.2(5); C67A–O2A–Sn2A, 123.3(3);

Plane angle between the two C_3N_2–motifs 74.2°

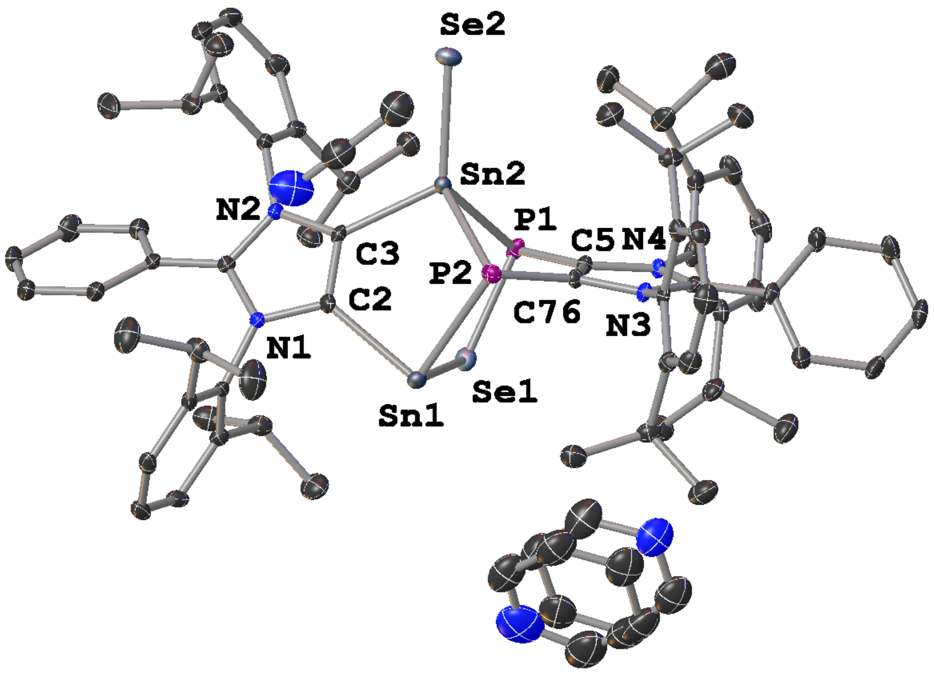

Figure S57. Molecular structure of **7**. H atoms are omitted for clarity. Thermal ellipsoids are shown with 50% probability**.** Selected bond lengths (Å) and angles (°): C2–C3, 1.370(5); C2–Sn1, 2.243(3); C3–Sn2, 2.158(3); Sn1–Se1, 2.681(1); Sn1–P2, 2.657(1); Sn2–Se2, 2.415(1); Sn2–P1, 2.618(1); Sn2–P2, 2.551(1); Se1–P1, 2.252(1); P1–C5, 1.839(4); P2–C76, 1.844(3); C2–Sn1–P2, 90.0(1); C2–Sn1–Se1 88.1(1), ;Sn1–P2–C76, 101.3(1); Sn1–Se1–P1, 106.4(1); Sn1–P2–Sn2, 91.3(1); C3–Sn2–P1, 100.9(1); C3–Sn2–P2, 97.0(1); P1–C5–C76, 128.5(3); C3–Sn2–Se2 118.5(1); P2–C76–C5, 127.4(2).

Plane angle between the two C_3N_2–motifs 60.9°

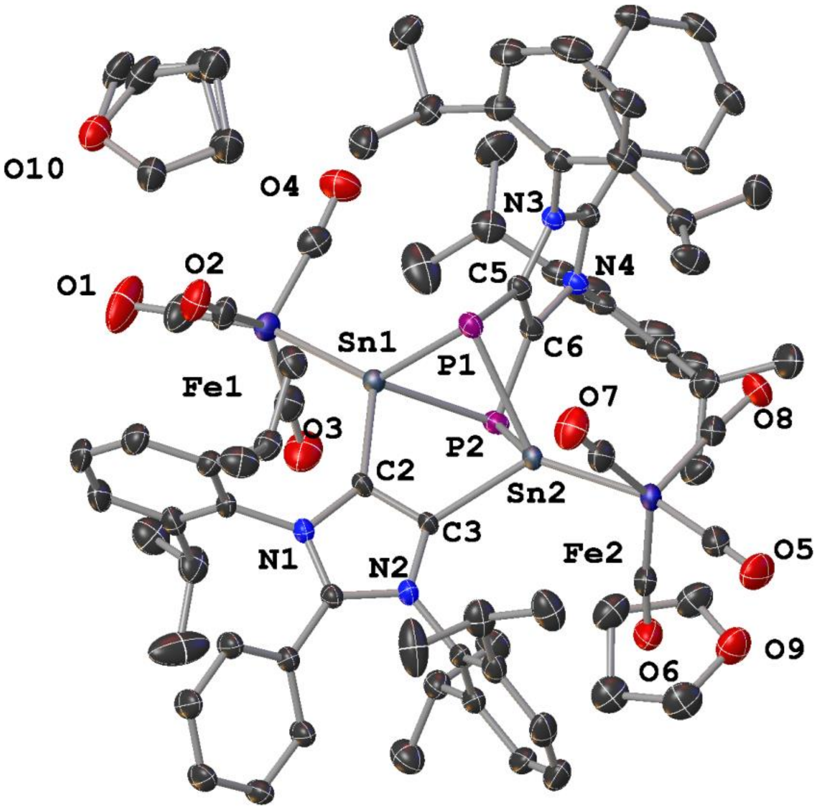

Figure S58. Molecular structure of **8**. H atoms are omitted for clarity. Thermal ellipsoids are shown with 50% probability. Selected bond lengths (Å) and angles (°): C2–C3, 1.367(4); C5–C6, 1.378(4); Sn1–C2, 2.197(3); Sn2–C3, 2.206(2); Sn1–P1, 2.586(1); Sn1–P2, 2.630(1); Sn2–P1, 2.648(1); Sn2–P2, 2.584(1); P1–C5, 1.823(3); P2–C6, 1.825(3); Sn1–Fe1, 2.505(1); Fe1–C68, 1.784(3); C68–O2, 1.153(4); C2–Sn1–P2, 96.3(1); Sn1–P1–C5, 86.5(1); Sn1–P1–Sn2, 78.5(1); Sn1–C2–C3, 115.7(2); P1–C5–C6, 124.2(2); C2–Sn1–Fe1, 122.6(1); P2–Sn1–Fe1, 117.8(1).

Plane angle between the two C_3N_2–motifs 74.1°

Computational Details

The molecular structures of **2** (Figure S59) and **5** (Figure S60) were optimized at the DFT PBE0-D3BJ/def2-TZVPP level of theory.[11] The calculations were performed with the Orca 5 program package unless otherwise stated.[12] For **5**, several electronic structures were considered in calculations, namely closed-shell singlet, UKS open-shell singlet-diradical, triplet, and quintet. The lowest energy was the closed-shell singlet solution. The open-shell singlet diradical was converged to the closed shell singlet electronic structure. Adiabatic relative energies of the triplet and quintet solutions were 26.9 and 66.6 kcal/mol, respectively. The optimized closed-shell singlet structure of **5** (see Figure S60 with the internal numeration of selected atoms) is C_2-symmetric with the atoms C9 and C81 lying on the symmetry axis. The optimized Cartesian coordinates are provided in Table S6.

We also analyzed the structure of **5** by fractional occupation weighted density (FOD) method. The FOD analysis was performed at the PBE0/def2-TZVPP level of theory with T_{el} = 10000 K. The resulting N_{FOD} number was 3.07 *e* indicating relatively large electron correlation.[13] The FOD electron density is shown in Figure S61.

The diradical character (*y*)[14] for **5** (*y* = 2%) was calculated from the occupation numbers of UHF natural orbitals obtained at the UHF/def2-TZVP level for a singlet-diradical solution. Note, initial spin flipping at one of the phosphor atoms or at one of the tin atoms converged to the same solution with <S2> = 2.865. However, the resulting Mulliken spin populations on P and Sn atoms were negligible (absolute values of the order 0.02 – 0.04 a.e.). Figure S60 (bottom) shows the respective spin density.

TD-DFT calculations were performed at the PBE0/def2-TZVPP level using TDA approximation and CPCM solution model for THF. The most intense transitions with oscillator strengths equal or larger then 0.02 are listed in Table S7. The frontier molecular orbitals of **5** in the PBE0/def2-TZVPP calculation are shown in Figure S63. NBO analysis[15] and Wiberg bond orders (see Table S8) were calculated at the PBE0/def2-TZVPP level using the Gaussian program package.[16]

Furthermore, to investigate the static electron correlation in **5**, we performed a series of state-averaged SA-CASSCF calculations (10 triplet and 10 singlet roots). The most balanced solution was obtained for a CASSCF(8,8)/def2-TZVPP variant. The optimized CAS orbitals from this calculation are shown in Figure S64. The lowest energy root was singlet with the following occupation pattern: 79% “22220000” and 10% “22211000”. The populations of natural orbitals in the CASSCF(8,8) calculation were 1.84, 1.83, 1.55, 1.34, 0.67, 0.66, 0.06, 0.05. Also, the calculated UV/Vis transitions are listed in Table S9.

To assess the diradical character (β),[17] state-specific SS-CASSCF calculations were performed. Different starting orbitals were tested: canonical HF and DFT, natural MP2 and those from FOD calculations, and from SA-CASSCF calculations. Also different active spaces up to (12,12) were investigated in order to obtain a well-converged and balanced solution. Most of the calculations were

unstable. All attempts to include orbitals located at P and Sn atoms into active space failed. Finally, we obtained a converged singlet ground state solution at the CASSCF/def2-TZVPP level for a (8,8) active space (Figure S65) with an occupation pattern of "22220000" (91%) and four other double excited configurations with contributions around 1.1 – 1.5 % each. This type of solution does not allow a reliable calculation of diradical character. Formally, taking the largest minor configuration we obtain $\beta = 3\%$ for **5**, which is essentially negligible.

For comparison, the molecular structure of the putative phosphinidene species **4a** (see Figure S62) was also modeled. At the PBE0-D3BJ/def2-TZVPP level. The lowest energy had the triplet structure whereas the closed shell singlet was only 0.05 kcal/mol higher (adiabatic energy difference). No stable open-shell singlet diradical solution was found for **4a**. In comparison to **5** the energy of **4a** was 104 kcal/mol higher.

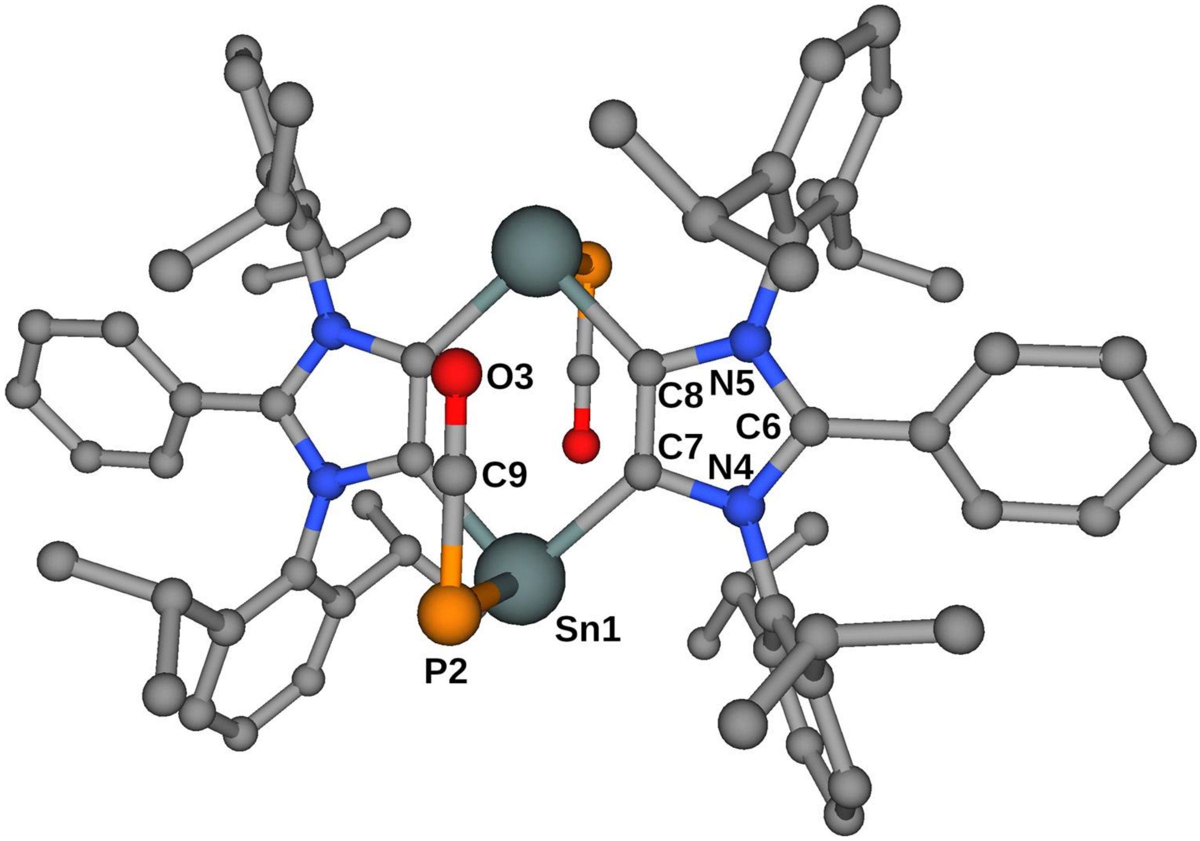

Figure S59. Equilibrium molecular structure of **2** optimized at the PBE0-D3BJ/def2-TZVPP level of theory. Selected geometrical parameters [Å, degrees] are r(Sn1–P2) = 2.680, r(P2–C9) = 1.633, r(C9–O3) = 1.172, r(Sn1–C7) = 2.236, r(C7–C8) = 1.374, r(C7–N4) = 1.396, r(C8–N5) = 1.396, r(N4–C6) = 1.346, r(N5–C6) = 1.346, α(Sn1–P2–C9) = 90.6.

Table S4. NBO charges *q* and Wiberg bond indices (larger than 0.1) for selected atoms and atom pairs in **2** as calculated at the PBE0/def2-TZVPP level of theory. For numeration of atoms see Figure S59.

Atom	*q*	Atom								
		Sn1	P2	O3	N4	N5	C6	C7	C8	C9
Sn1	0.82		0.56					0.59		0.12
P2	-0.19	0.56		0.35						1.90
O3	-0.55		0.35							1.77
N4	-0.36						1.25	1.09		
N5	-0.36						1.25		1.10	
C6	0.39				1.25	1.25		0.11	0.11	
C7	-0.27	0.59			1.09		0.11		1.57	
C8	-0.27					1.10	0.11	1.57		
C9	0.22	0.12	1.90	1.77						

Table S5. Optimized Cartesian coordinates of atoms in **2** at the PBE0-D3BJ/def2-TZVPP level of theory.

Sn	0.714620418	2.114273516	0.031525154	C	-2.269876031	3.181543010	-5.073422169
P	-1.748936174	3.151428547	0.231942968	H	-2.045626294	4.040852882	-5.710167683
O	-2.902539664	0.595519121	0.243888752	H	-3.354974456	3.120865318	-4.965960660
N	0.157614644	1.130758065	-2.934477284	H	-1.928684704	2.287224441	-5.590499717
N	-0.644920521	-0.861990954	-2.961511227	C	-2.179051342	4.607894198	-3.034251557
C	-0.310266364	0.172377026	-3.754968416	H	-3.243014781	4.475087796	-2.831294600
C	0.114915894	0.711114665	-1.603304207	H	-2.071843514	5.481655892	-3.681160555
C	-0.378102867	-0.570791353	-1.622167248	H	-1.684884378	4.812136174	-2.086179354
C	-2.397571330	1.652671062	0.239440090	C	3.027530906	1.343920052	-2.939349984
C	-0.440720879	0.238297102	-5.211351498	H	2.446101693	0.686243456	-2.294606566
C	-1.505497774	-0.393241297	-5.862538445	C	3.551920579	0.500902292	-4.100420602
H	-2.246866230	-0.934140802	-5.297343359	H	4.124082395	1.112567745	-4.802981552
C	-1.633156720	-0.329761349	-7.238219435	H	2.748533441	0.014794866	-4.655051825
H	-2.469663768	-0.825885149	-7.713985117	H	4.210246517	-0.282339200	-3.719382260
C	-0.704897789	0.362898403	-7.999879836	C	4.185074699	1.866583384	-2.097726515
H	-0.806465957	0.410615479	-9.076894523	H	4.905732931	2.429020772	-2.695853841
C	0.354135033	0.994237702	-7.366963823	H	4.711313713	1.026718453	-1.643078987
H	1.090485432	1.538353148	-7.944861692	H	3.823231861	2.511558599	-1.296310843
C	0.487009583	0.935420584	-5.991282563	C	-1.212196845	-2.093858295	-3.425606354
H	1.322376783	1.436245600	-5.530065453	C	-0.411162671	-3.001108988	-4.124100032
C	0.725288733	2.371179949	-3.376926186	C	-1.027265539	-4.149265673	-4.615287318
C	-0.111520021	3.409996808	-3.794404484	H	-0.434983214	-4.869575812	-5.165538083
C	0.497784602	4.546928112	-4.317575683	C	-2.371202812	-4.384851329	-4.410025329
H	-0.121743095	5.366555125	-4.658541731	H	-2.828984146	-5.285263996	-4.801700195
C	1.872537087	4.650606398	-4.402779751	C	-3.134457449	-3.480135760	-3.690136684
H	2.322715209	5.544418718	-4.817705665	H	-4.181953432	-3.686533226	-3.518121433
C	2.676415379	3.623651185	-3.939249395	C	-2.577909928	-2.314565925	-3.181668448
H	3.753141367	3.723448197	-3.986876667	C	1.065712066	-2.797810248	-4.379175011
C	2.124876999	2.462524530	-3.413645434	H	1.384420478	-1.900163063	-3.845017485
C	-1.619596477	3.354298370	-3.701272954	C	1.358736818	-2.613753025	-5.868028004
H	-1.885996090	2.502533288	-3.071060529	H	1.114679843	-3.524781242	-6.419417470

H	2.422141304	-2.413770415	-6.015651072
H	0.792923808	-1.795269417	-6.308765995
C	1.887036717	-3.965965899	-3.841594063
H	1.706616067	-4.124629962	-2.779716082
H	2.951660756	-3.761055893	-3.971967417
H	1.659190526	-4.888425732	-4.380408783
C	-3.427858418	-1.320433398	-2.418364901
H	-2.805825074	-0.882550180	-1.637913448
C	-4.613565332	-1.967775563	-1.719427105
H	-5.383155399	-2.288375627	-2.426129156
H	-5.070133407	-1.243838202	-1.043669637
H	-4.303762797	-2.832001996	-1.130160968
C	-3.905515129	-0.163059557	-3.294255934
H	-3.079017966	0.412503943	-3.710353643
H	-4.509232122	0.521648133	-2.695862224
H	-4.520366493	-0.523314575	-4.123449724
Sn	-0.714620418	-2.114273516	-0.031525154
P	1.748936174	-3.151428547	-0.231942968
O	2.902539664	-0.595519121	-0.243888752
N	-0.157614644	-1.130758065	2.934477284
N	0.644920521	0.861990954	2.961511227
C	0.310266364	-0.172377026	3.754968416
C	-0.114915894	-0.711114665	1.603304207
C	0.378102867	0.570791353	1.622167248
C	2.397571330	-1.652671062	-0.239440090
C	0.440720879	-0.238297102	5.211351498
C	1.505497774	0.393241297	5.862538445
H	2.246866230	0.934140802	5.297343359
C	1.633156720	0.329761349	7.238219435
H	2.469663768	0.825885149	7.713985117
C	0.704897789	-0.362898403	7.999879836
H	0.806465957	-0.410615479	9.076894523
C	-0.354135033	-0.994237702	7.366963823

H	-1.090485432	-1.538353148	7.944861692
C	-0.487009583	-0.935420584	5.991282563
H	-1.322376783	-1.436245600	5.530065453
C	-0.725288733	-2.371179949	3.376926186
C	0.111520021	-3.409996808	3.794404484
C	-0.497784602	-4.546928112	4.317575683
H	0.121743095	-5.366555125	4.658541731
C	-1.872537087	-4.650606398	4.402779751
H	-2.322715209	-5.544418718	4.817705665
C	-2.676415379	-3.623651185	3.939249395
H	-3.753141367	-3.723448197	3.986876667
C	-2.124876999	-2.462524530	3.413645434
C	1.619596477	-3.354298370	3.701272954
H	1.885996090	-2.502533288	3.071060529
C	2.269876031	-3.181543010	5.073422169
H	2.045626294	-4.040852882	5.710167683
H	3.354974456	-3.120865318	4.965960660
H	1.928684704	-2.287224441	5.590499717
C	2.179051342	-4.607894198	3.034251557
H	3.243014781	-4.475087796	2.831294600
H	2.071843514	-5.481655892	3.681160555
H	1.684884378	-4.812136174	2.086179354
C	-3.027530906	-1.343920052	2.939349984
H	-2.446101693	-0.686243456	2.294606566
C	-3.551920579	-0.500902292	4.100420602
H	-4.124082395	-1.112567745	4.802981552
H	-2.748533441	-0.014794866	4.655051825
H	-4.210246517	0.282339200	3.719382260
C	-4.185074699	-1.866583384	2.097726515
H	-4.905732931	-2.429020772	2.695853841
H	-4.711313713	-1.026718453	1.643078987
H	-3.823231861	-2.511558599	1.296310843
C	1.212196845	2.093858295	3.425606354

C	0.411162671	3.001108988	4.124100032
C	1.027265539	4.149265673	4.615287318
H	0.434983214	4.869575812	5.165538083
C	2.371202812	4.384851329	4.410025329
H	2.828984146	5.285263996	4.801700195
C	3.134457449	3.480135760	3.690136684
H	4.181953432	3.686533226	3.518121433
C	2.577909928	2.314565925	3.181668448
C	-1.065712066	2.797810248	4.379175011
H	-1.384420478	1.900163063	3.845017485
C	-1.358736818	2.613753025	5.868028004
H	-1.114679843	3.524781242	6.419417470
H	-2.422141304	2.413770415	6.015651072
H	-0.792923808	1.795269417	6.308765995
C	-1.887036717	3.965965899	3.841594063
H	-1.706616067	4.124629962	2.779716082
H	-2.951660756	3.761055893	3.971967417
H	-1.659190526	4.888425732	4.380408783
C	3.427858418	1.320433398	2.418364901
H	2.805825074	0.882550180	1.637913448
C	4.613565332	1.967775563	1.719427105
H	5.383155399	2.288375627	2.426129156
H	5.070133407	1.243838202	1.043669637
H	4.303762797	2.832001996	1.130160968
C	3.905515129	0.163059557	3.294255934
H	3.079017966	-0.412503943	3.710353643
H	4.509232122	-0.521648133	2.695862224
H	4.520366493	0.523314575	4.123449724

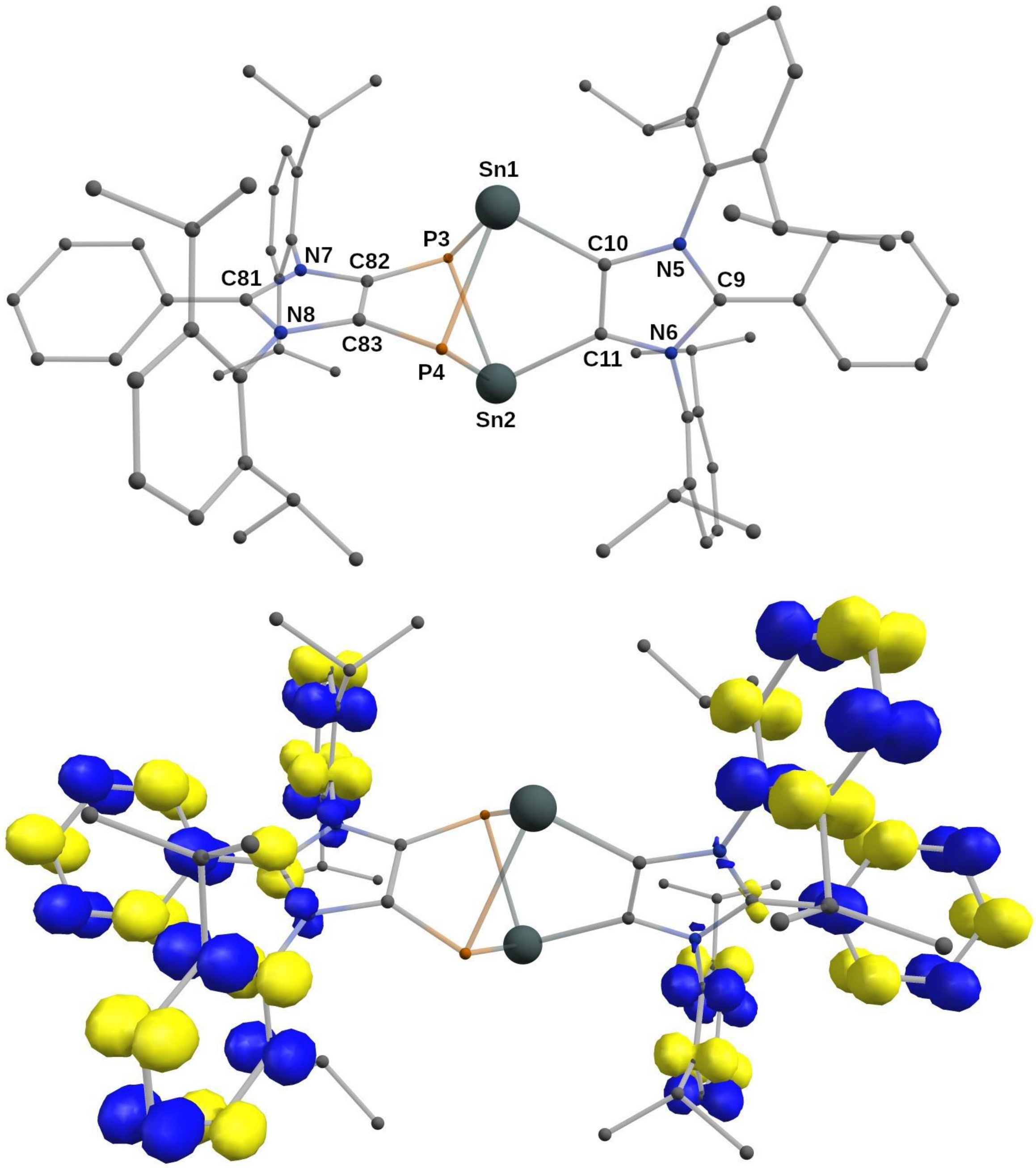

Figure S60. Optimized free molecular structure of **5** (top) at the RKS-PBE0-D3BJ/def2-TZVPP level. Hydrogen atoms are omitted for clarity. Selected equilibrium parameters (Å, degrees) are: r(Sn1-P3) = 2.670, r(Sn1-P4) = 2.686, r(Sn1-C10) = *2.250*, r(P3-C82) = 1.814, r(C10-C11) = 1.376, r(C82-C83) = 1.389. Isosurface (0.02 a.e.) of spin density for **5** (bottom) corresponding to the singlet-diradical solution at UHF/def2-TZVP. Hydrogen atoms are omitted for clarity.

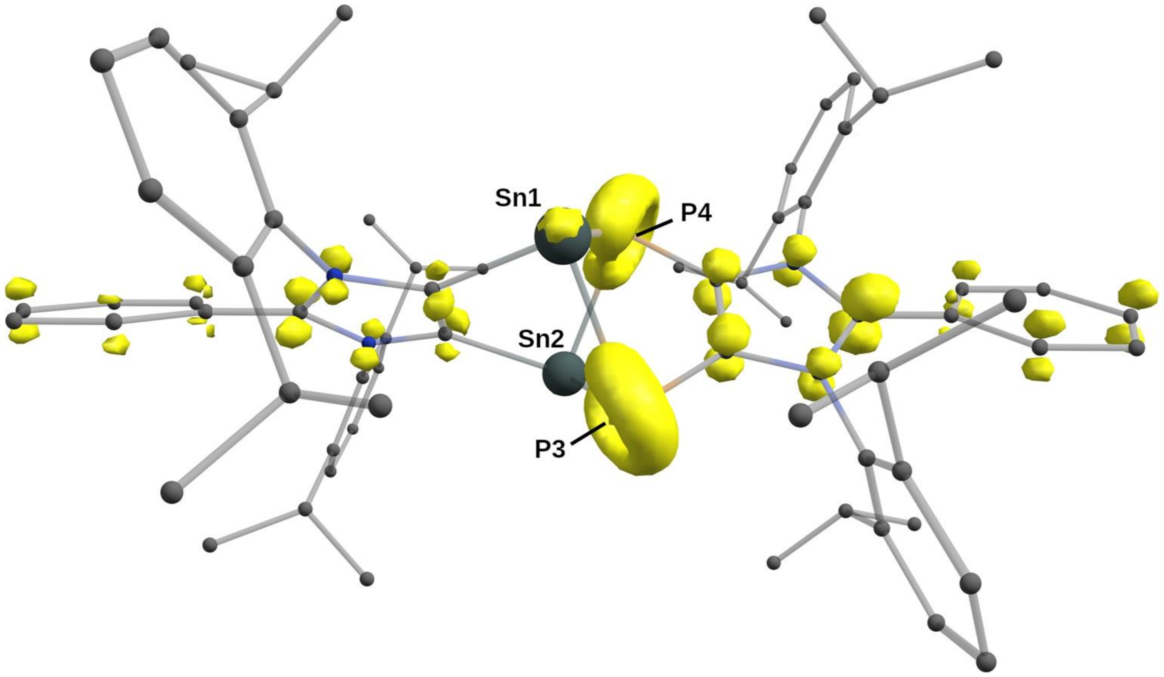

Figure S61. Isosurface (0.005 a.e., plotted in yellow) of FOD for **5**. Hydrogen atoms are omitted for clarity.

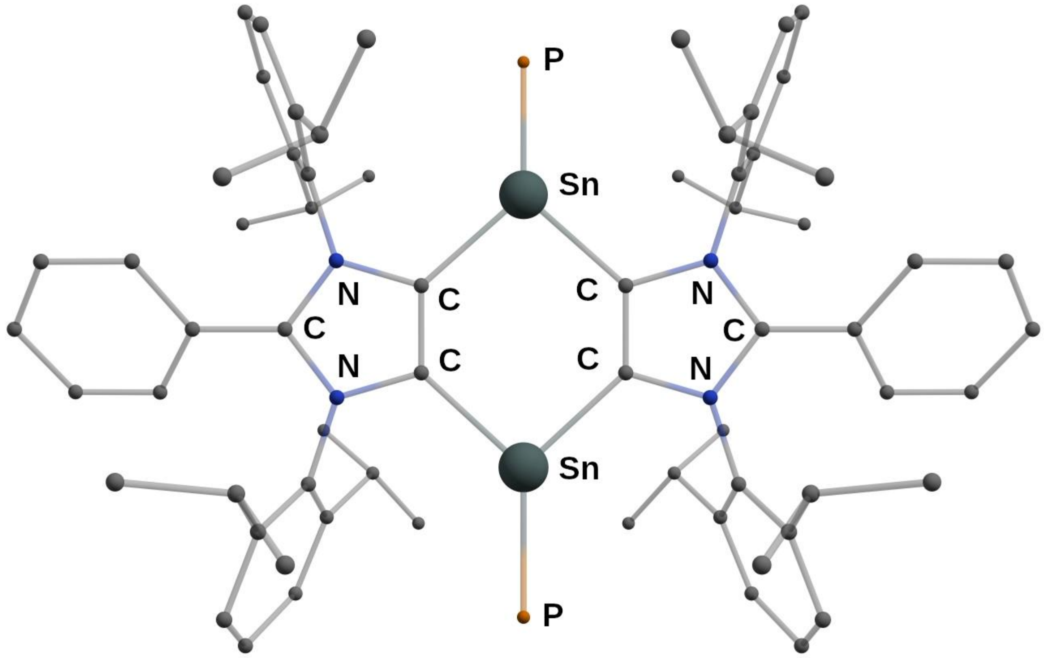

Figure S62. Optimized free molecular structure of **4a** at the RKS-PBE0-D3BJ/def2-TZVPP level. Hydrogen atoms are omitted for clarity.

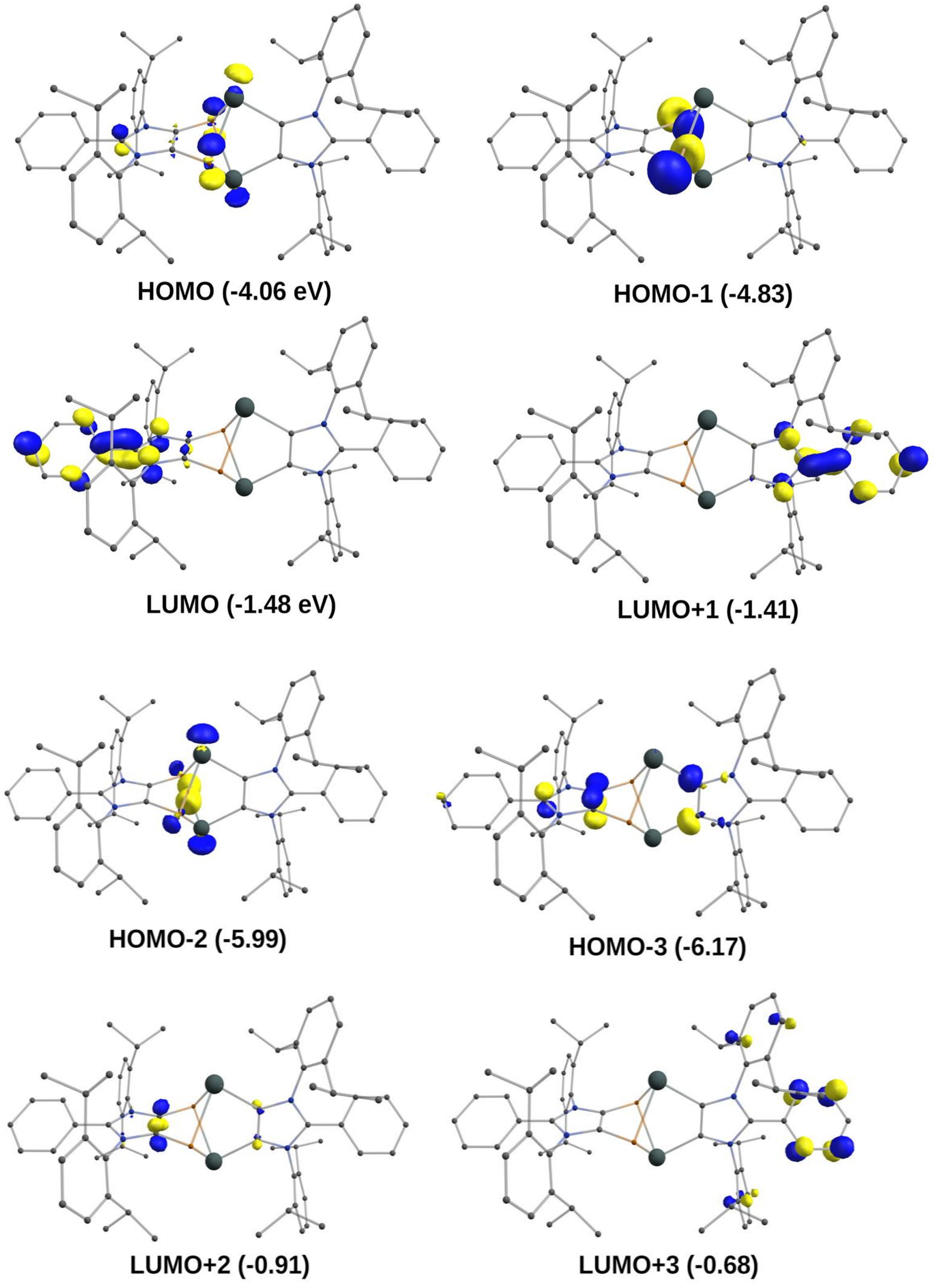

Figure S63. Frontier molecular orbitals (0.07 isosurfaces plotted in blue and yellow) of **5** in PBE0/def2-TZVPP calculation. Orbital energies in parentheses are in eV.

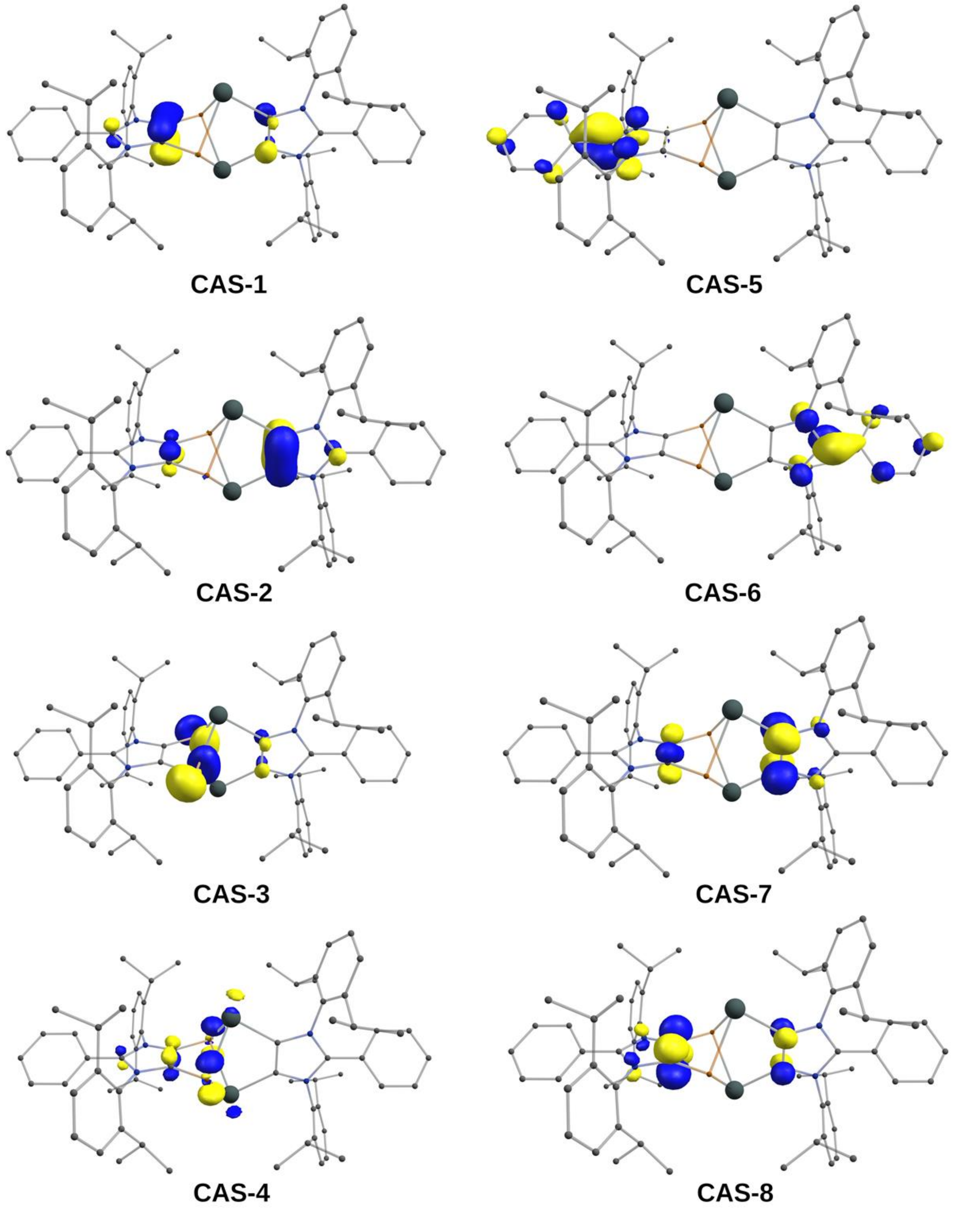

Figure S64. Optimized CAS orbitals in SA-CASSCF(8,8)/def2-TZVPP calculation (0.07 isosurfaces plotted in blue and yellow) for **5**. Hydrogen atoms are omitted for clarity.

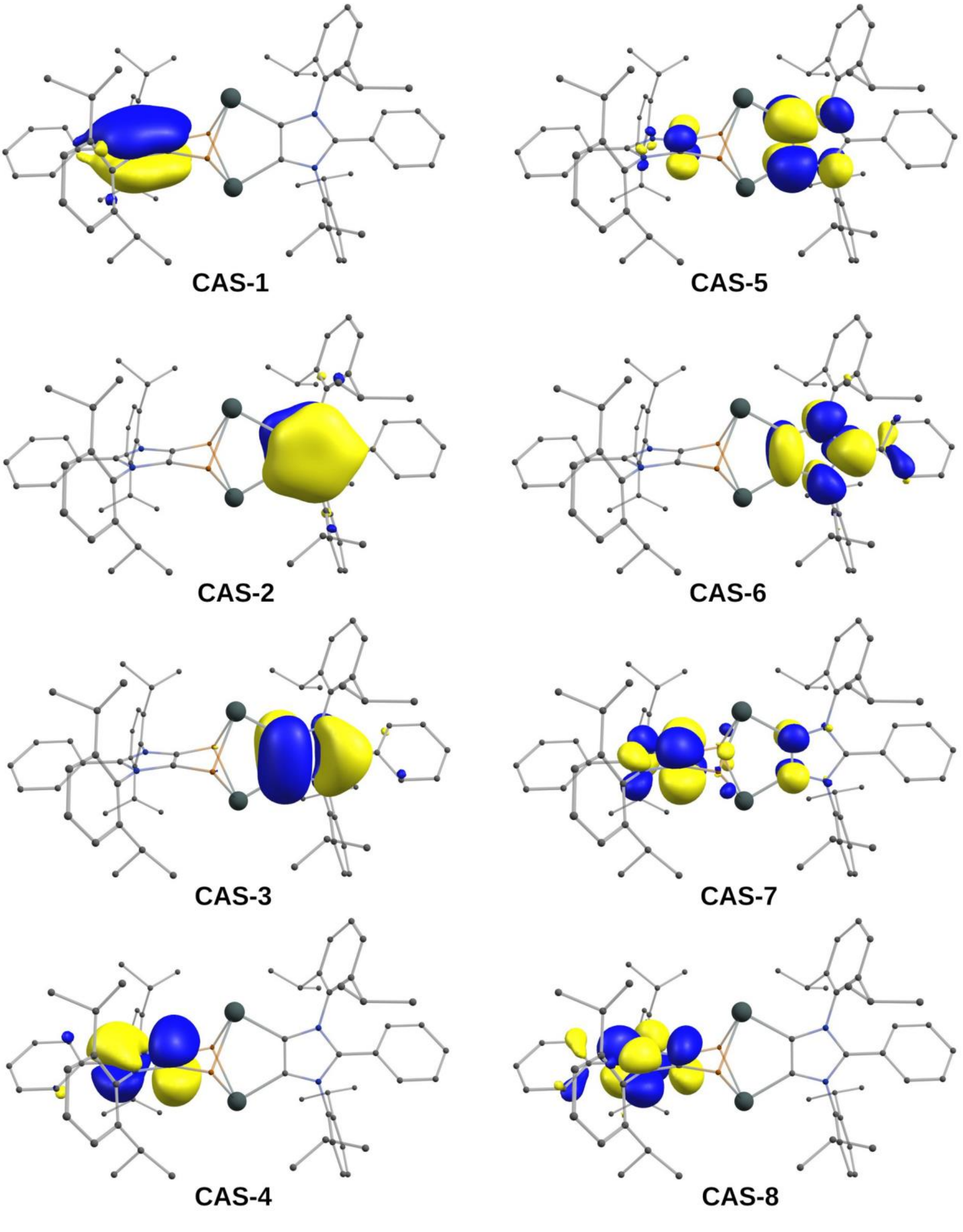

Figure S65. Optimized CAS orbitals in SS-CASSCF(8,8)/def2-TZVPP calculation (0.03 isosurfaces plotted in blue and yellow) for **5**. Hydrogen atoms are omitted for clarity.

Table S6. Cartesian coordinates (Å) of atoms in **5** optimized at the RKS-PBE0-D3BJ/def2-TZVPP level of theory.

Sn	-1.606746794	-0.869096744	0.234925919	C	-3.328099802	1.287256408	3.228018425
Sn	1.606746794	0.869096744	0.234925919	H	-2.306039697	1.478558448	2.898688324
P	-0.826022152	1.501899261	-0.711465703	C	-3.683248619	2.335416070	4.279887533
P	0.826022152	-1.501899261	-0.711465703	H	-4.696470648	2.186674675	4.660159298
N	-0.996344676	-0.401238738	3.500943601	H	-3.634215753	3.334919224	3.843078110
N	0.996344676	0.401238738	3.500943601	H	-2.998945171	2.310288679	5.130023433
N	-0.569380828	0.917568553	-3.530985842	C	-4.226791654	1.427074876	2.002752746
N	0.569380828	-0.917568553	-3.530985842	H	-3.991429926	0.670926834	1.251235790
C	0.000000000	0.000000000	4.317820439	H	-4.091470145	2.409317066	1.546752924
C	-0.633208334	-0.268937756	2.172979928	H	-5.281059865	1.319759564	2.268893958
C	0.633208334	0.268937756	2.172979928	C	2.255761951	0.926939069	3.918513977
C	-2.255761951	-0.926939069	3.918513977	C	3.387765947	0.115982450	3.791985229
C	-2.305167987	-2.230686833	4.412609823	C	4.600398798	0.646221291	4.216358129
C	-3.542512097	-2.712777826	4.823686126	H	5.498408972	0.045651419	4.136313546
H	-3.617499873	-3.721578096	5.211090253	C	4.678036831	1.927665398	4.733911386
C	-4.678036831	-1.927665398	4.733911386	H	5.633506489	2.321288352	5.059011607
H	-5.633506489	-2.321288352	5.059011607	C	3.542512097	2.712777826	4.823686126
C	-4.600398798	-0.646221291	4.216358129	H	3.617499873	3.721578096	5.211090253
H	-5.498408972	-0.045651419	4.136313546	C	2.305167987	2.230686833	4.412609823
C	-3.387765947	-0.115982450	3.791985229	C	3.328099802	-1.287256408	3.228018425
C	-1.084204856	-3.118613901	4.465462686	H	2.306039697	-1.478558448	2.898688324
H	-0.217162727	-2.522828537	4.176939383	C	3.683248619	-2.335416070	4.279887533
C	-0.825788554	-3.649367900	5.871501057	H	4.696470648	-2.186674675	4.660159298
H	-0.724639766	-2.834382432	6.590084416	H	3.634215753	-3.334919224	3.843078110
H	0.094797844	-4.236436480	5.888821822	H	2.998945171	-2.310288679	5.130023433
H	-1.637061443	-4.298419183	6.208281056	C	4.226791654	-1.427074876	2.002752746
C	-1.207824246	-4.250555382	3.448366663	H	3.991429926	-0.670926834	1.251235790
H	-2.055519418	-4.898538292	3.682708501	H	4.091470145	-2.409317066	1.546752924
H	-0.304037166	-4.863369500	3.449232041	H	5.281059865	-1.319759564	2.268893958
H	-1.353757708	-3.858340034	2.440350964	C	1.084204856	3.118613901	4.465462686

H	0.217162727	2.522828537	4.176939383
C	0.825788554	3.649367900	5.871501057
H	0.724639766	2.834382432	6.590084416
H	-0.094797844	4.236436480	5.888821822
H	1.637061443	4.298419183	6.208281056
C	1.207824246	4.250555382	3.448366663
H	2.055519418	4.898538292	3.682708501
H	0.304037166	4.863369500	3.449232041
H	1.353757708	3.858340034	2.440350964
C	0.000000000	0.000000000	5.774625387
C	1.166884167	-0.282412707	6.490845672
H	2.082549466	-0.505840832	5.965027972
C	1.164099421	-0.279876179	7.874209792
H	2.080266447	-0.503643738	8.406368133
C	0.000000000	0.000000000	8.574269724
H	0.000000000	0.000000000	9.657100551
C	-1.164099421	0.279876179	7.874209792
H	-2.080266447	0.503643738	8.406368133
C	-1.166884167	0.282412707	6.490845672
H	-2.082549466	0.505840832	5.965027972
C	0.000000000	0.000000000	-4.355845465
C	-0.352414411	0.598179451	-2.211362750
C	0.352414411	-0.598179451	-2.211362750
C	-1.228912513	2.114703903	-3.940049822
C	-0.449396075	3.170754627	-4.410171774
C	-1.115497622	4.312261140	-4.841433805
H	-0.542921621	5.152871943	-5.214042090
C	-2.495707720	4.391546401	-4.791572669
H	-2.997297099	5.288053695	-5.135771009
C	-3.238200864	3.337305410	-4.286872570
H	-4.316979493	3.418854752	-4.233556142
C	-2.621122942	2.175179532	-3.840470105
C	1.060179860	3.118340756	-4.408646835
H	1.366264902	2.107213647	-4.136224210
C	1.614853568	4.056924984	-3.339421420
H	1.355264229	5.095618844	-3.557695792
H	2.703347410	3.981624801	-3.294835128
H	1.213265619	3.806775873	-2.355837822
C	1.647342928	3.413338968	-5.784240286
H	1.258511816	2.724375649	-6.536005463
H	2.734359009	3.312625927	-5.757669959
H	1.419194958	4.431460731	-6.107293716
C	-3.447033621	1.034514760	-3.288275108
H	-2.765002591	0.293979560	-2.867919543
C	-4.345171870	1.503068917	-2.148225723
H	-3.753545680	1.999990983	-1.378527057
H	-4.846883783	0.646314897	-1.693925536
H	-5.114777354	2.194073451	-2.500388172
C	-4.259682411	0.346069518	-4.382365534
H	-4.948759887	1.045992514	-4.861231048
H	-4.850233886	-0.466840967	-3.954712739
H	-3.619628458	-0.078992381	-5.157688658
C	1.228912513	-2.114703903	-3.940049822
C	0.449396075	-3.170754627	-4.410171774
C	1.115497622	-4.312261140	-4.841433805
H	0.542921621	-5.152871943	-5.214042090
C	2.495707720	-4.391546401	-4.791572669
H	2.997297099	-5.288053695	-5.135771009
C	3.238200864	-3.337305410	-4.286872570
H	4.316979493	-3.418854752	-4.233556142
C	2.621122942	-2.175179532	-3.840470105
C	-1.060179860	-3.118340756	-4.408646835
H	-1.366264902	-2.107213647	-4.136224210
C	-1.614853568	-4.056924984	-3.339421420
H	-1.355264229	-5.095618844	-3.557695792
H	-2.703347410	-3.981624801	-3.294835128

H	-1.213265619	-3.806775873	-2.355837822
C	-1.647342928	-3.413338968	-5.784240286
H	-1.258511816	-2.724375649	-6.536005463
H	-2.734359009	-3.312625927	-5.757669959
H	-1.419194958	-4.431460731	-6.107293716
C	3.447033621	-1.034514760	-3.288275108
H	2.765002591	-0.293979560	-2.867919543
C	4.345171870	-1.503068917	-2.148225723
H	3.753545680	-1.999990983	-1.378527057
H	4.846883783	-0.646314897	-1.693925536
H	5.114777354	-2.194073451	-2.500388172
C	4.259682411	-0.346069518	-4.382365534
H	4.948759887	-1.045992514	-4.861231048

H	4.850233886	0.466840967	-3.954712739
H	3.619628458	0.078992381	-5.157688658
C	0.000000000	0.000000000	-5.804868272
C	1.054919637	-0.572553691	-6.530799322
H	1.888893360	-1.019974416	-6.013467392
C	1.048861780	-0.573253361	-7.913127104
H	1.880125446	-1.022422442	-8.442633727
C	0.000000000	0.000000000	-8.617383550
H	0.000000000	0.000000000	-9.700079737
C	-1.048861780	0.573253361	-7.913127104
H	-1.880125446	1.022422442	-8.442633727
C	-1.054919637	0.572553691	-6.530799322
H	-1.888893360	1.019974416	-6.013467392

Table S7. Most important transitions in the TD-DFT absorption spectrum (vertical energy differences) of **5**. Wavelengths in nm, oscillator strengths via transition electric dipole moments and assignments are listed.

584 0.40 82% HOMO → LUMO, 16% HOMO → LUMO+1

432 0.20 92% HOMO-1 → LUMO+1

306 0.13 51% HOMO-2 → LUMO+2, 18% HOMO-3 → LUMO, 11% HOMO-3 → LUMO+1

304 0.23 36% HOMO-2 → LUMO+2, 32% HOMO-3 → LUMO, 22% HOMO-3 → LUMO+1

302 0.47 46% HOMO-3 → LUMO+1, 40% HOMO-3 → LUMO

Table S8. NBO charges (*e*) and Wiberg bond orders (larger than 0.05) for selected atoms and atom pairs in **5**. For atom numbering see Figure S.

Atom	*q*	Sn1	Sn2	P3	P4	N5	N6	N7	N8	C9	C10	C11	C81	C82	C83
Sn1	0.53			0.70	0.70						0.61				
Sn2	0.53			0.70	0.70							0.61			
P3	-0.30	0.70	0.70											1.02	
P4	-0.30	0.70	0.70												1.02
N5	-0.36						0.08			1.23	1.11				
N6	-0.36					0.08				1.23		1.11			
N7	-0.35												1.19	1.10	
N8	-0.35												1.19		1.10
C9	0.35					1.23	1.23								
C10	-0.24	0.61				1.11						1.56			
C11	-0.24		0.61				1.11				1.56				
C81	0.34							1.19	1.19						
C82	-0.15			1.02				1.10							1.38
C83	-0.15				1.02				1.10					1.38	

Table S9. Most important transitions in the CASSCF(8,8) calculation (vertical energy differences) of **5**. Wavelengths in nm, oscillator strengths and assignments are listed.

492 0.76 $S_0 \rightarrow S_2$

387 0.30 $S_0 \rightarrow S_4$

218 0.50 $S_0 \rightarrow S_7$

211 0.60 $S_0 \rightarrow S_8$

We also calculated the energy of the reaction of **5** with CO_2 to give **6** (Figure S66a) as well as the further reaction of **6** with an additional CO_2 to yield a hypothetical product **6**-CO_2 (Figure S66b) at the PBE0-D3BJ/def2-TZVPP level of theory.

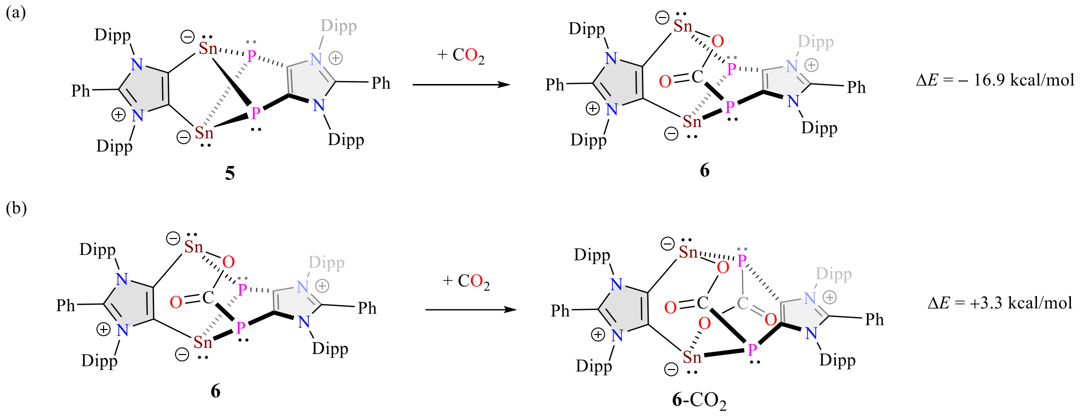

Figure S66. Calculated reactions (and energies) of **5** and **6** with CO_2 to give **6** and **6**-CO_2, respectively.

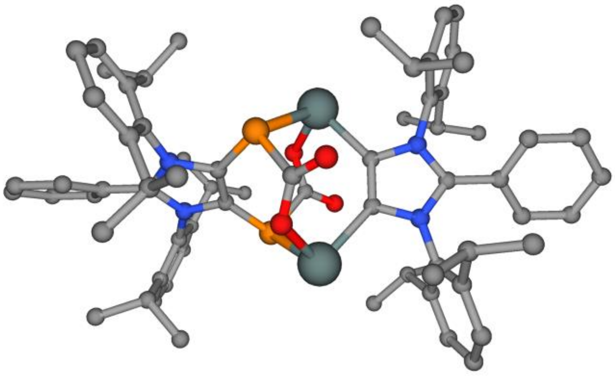

Figure S67. The optimized structure of **6**-CO_2 at the PBE0-D3BJ/def2-TZVPP level of theory. H atoms are omitted for clarity.

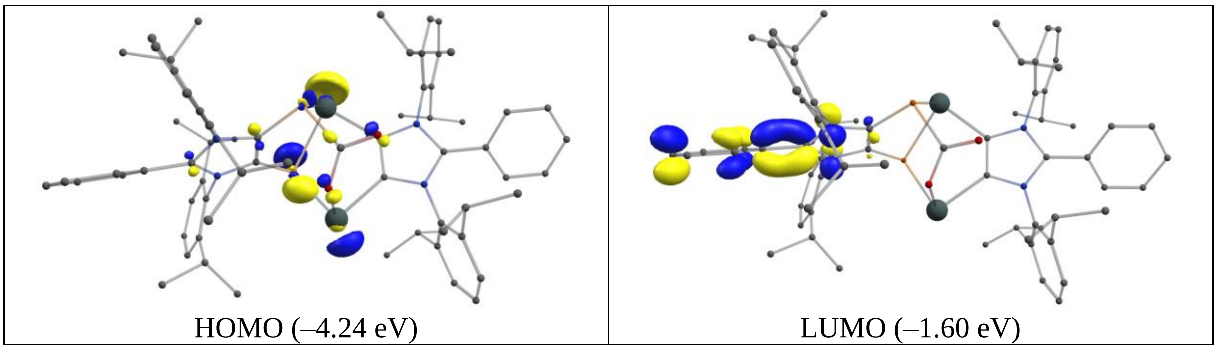

Figure S68. Frontier molecular orbitals (0.07 isosurfaces plotted in blue and yellow) of **6** at the PBE0/def2-TZVPP level of theory.

Table S10. Cartesian coordinates (Å) of **6**-CO_2.

Sn	2.141071913	0.949450352	-0.572874515	C	-1.148819404	0.345326264	-6.440172287
P	-1.669778784	1.124388960	1.029034883	C	1.148819404	-0.345326264	-6.440172287
P	1.669778784	-1.124388960	1.029034883	C	4.620434452	0.256755508	-4.230934324
C	0.656129590	0.211556701	-2.110722750	C	3.207965538	-1.570171765	-3.215359384
Sn	-2.141071913	-0.949450352	-0.572874515	C	3.728118965	2.417782518	-4.781934050
C	-0.547598755	0.419524826	2.277372884	C	1.320227517	3.025494360	-4.358313446
C	0.547598755	-0.419524826	2.277372884	C	-3.378831688	0.176271823	-3.780716617
C	0.551436218	-2.166640313	-0.015718891	C	-2.463314533	-2.037024338	-4.347819490
N	1.023093593	0.307434970	-3.448084641	C	0.342197578	-1.150526775	6.604846821
C	-0.656129590	-0.211556701	-2.110722750	C	-0.342197578	1.150526775	6.604846821
O	-0.704545733	-2.237792794	0.312408139	C	-4.041992651	1.711292305	5.100018015
N	-0.838169094	0.668404865	3.617954157	C	-3.235291808	-0.613429224	4.565391909
N	0.838169094	-0.668404865	3.617954157	C	-2.777236950	3.666899293	4.514629133
O	1.075994324	-2.767050768	-0.936419074	C	-0.540202478	3.537122823	3.360534817
C	0.000000000	0.000000000	-4.262135590	C	4.041992651	-1.711292305	5.100018015
C	2.317778108	0.731749056	-3.876125790	C	3.235291808	0.613429224	4.565391909
N	-1.023093593	-0.307434970	-3.448084641	C	2.777236950	-3.666899293	4.514629133
C	0.000000000	0.000000000	4.426062213	C	0.540202478	-3.537122823	3.360534817
C	-1.911101470	1.501483493	4.068949320	H	-2.052877295	0.616875684	-5.918948509
C	1.911101470	-1.501483493	4.068949320	C	-1.145659735	0.344969726	-7.823574286
C	0.000000000	0.000000000	-5.723103949	C	1.145659735	-0.344969726	-7.823574286
C	3.378831688	-0.176271823	-3.780716617	H	2.052877295	-0.616875684	-5.918948509
C	2.463314533	2.037024338	-4.347819490	H	5.466787953	-0.416843580	-4.177820839
C	-2.317778108	-0.731749056	-3.876125790	C	4.792396417	1.535139687	-4.734799158
C	0.000000000	0.000000000	5.888888451	H	2.232318514	-1.639930614	-2.734235219
C	-3.046226852	0.884922738	4.591319679	C	3.252841319	-2.640092865	-4.304006117
C	-1.754789785	2.888466541	3.987805230	C	4.242612665	-1.871433386	-2.135542529
C	3.046226852	-0.884922738	4.591319679	H	3.880787864	3.423736147	-5.153516863
C	1.754789785	-2.888466541	3.987805230	H	0.419275087	2.514972018	-4.015845897

C	1.047663010	3.555975681	-5.762378747
C	1.579966371	4.159992042	-3.370730117
C	-4.620434452	-0.256755508	-4.230934324
C	-3.207965538	1.570171765	-3.215359384
C	-3.728118965	-2.417782518	-4.781934050
C	-1.320227517	-3.025494360	-4.358313446
H	0.611444954	-2.055723796	6.084706489
C	0.339948388	-1.147490787	7.988132154
C	-0.339948388	1.147490787	7.988132154
H	-0.611444954	2.055723796	6.084706489
H	-4.939762844	1.269214268	5.514119400
C	-3.903474978	3.086801127	5.074932556
H	-2.305458595	-1.072196739	4.225249358
C	-4.311978976	-0.987306291	3.549026623
C	-3.542852474	-1.179443105	5.947156294
H	-2.694534134	4.745602661	4.475104558
H	-0.004490242	2.782223533	2.784789578
C	-0.937416659	4.630914060	2.374967262
C	0.423447133	4.082493501	4.412608112
H	4.939762844	-1.269214268	5.514119400
C	3.903474978	-3.086801127	5.074932556
H	2.305458595	1.072196739	4.225249358
C	4.311978976	0.987306291	3.549026623
C	3.542852474	1.179443105	5.947156294
H	2.694534134	-4.745602661	4.475104558
H	0.004490242	-2.782223533	2.784789578
C	0.937416659	-4.630914060	2.374967262
C	-0.423447133	-4.082493501	4.412608112
H	-2.048447004	0.620216377	-8.354299066
C	0.000000000	0.000000000	-8.523934454
H	2.048447004	-0.620216377	-8.354299066
H	5.769732039	1.850934155	-5.079623411
H	2.444031767	-2.524725047	-5.027475452
H	3.148941841	-3.627354315	-3.850354088
H	4.200656333	-2.612879885	-4.848052057
H	5.247289867	-1.976442466	-2.552639115
H	3.978743773	-2.801745707	-1.631962580
H	4.276945722	-1.082096862	-1.381499048
H	1.896784715	4.128722537	-6.142177736
H	0.180408830	4.219210740	-5.750096373
H	0.847820828	2.744454124	-6.464044962
H	1.709587307	3.778882445	-2.356981562
H	0.731164106	4.845754156	-3.356864963
H	2.474222037	4.724968572	-3.644527739
H	-5.466787953	0.416843580	-4.177820839
C	-4.792396417	-1.535139687	-4.734799158
H	-2.232318514	1.639930614	-2.734235219
C	-3.252841319	2.640092865	-4.304006117
C	-4.242612665	1.871433386	-2.135542529
H	-3.880787864	-3.423736147	-5.153516863
H	-0.419275087	-2.514972018	-4.015845897
C	-1.047663010	-3.555975681	-5.762378747
C	-1.579966371	-4.159992042	-3.370730117
H	0.604985018	-2.053222031	8.518739945
C	0.000000000	0.000000000	8.687766754
H	-0.604985018	2.053222031	8.518739945
H	-4.687672671	3.715046889	5.479692647
H	-5.282546432	-0.579374716	3.840105655
H	-4.407653466	-2.072486826	3.477529871
H	-4.068839881	-0.598080990	2.558744649

H	-2.766976147	-0.913793692	6.667016046
H	-3.608367552	-2.268314647	5.900957630
H	-4.497051574	-0.808318345	6.326711492
H	-1.669117825	4.263365353	1.655728739
H	-0.058716249	4.958738998	1.818063903
H	-1.357070534	5.501599111	2.884027751
H	-0.069066684	4.816017846	5.055723640
H	1.265157669	4.575712320	3.922663842
H	0.828792523	3.294416628	5.049780931
H	4.687672671	-3.715046889	5.479692647
H	5.282546432	0.579374716	3.840105655
H	4.407653466	2.072486826	3.477529871
H	4.068839881	0.598080990	2.558744649
H	2.766976147	0.913793692	6.667016046
H	3.608367552	2.268314647	5.900957630
H	4.497051574	0.808318345	6.326711492
H	1.669117825	-4.263365353	1.655728739
H	0.058716249	-4.958738998	1.818063903
H	1.357070534	-5.501599111	2.884027751
H	0.069066684	-4.816017846	5.055723640
H	-1.265157669	-4.575712320	3.922663842
H	-0.828792523	-3.294416628	5.049780931
H	0.000000000	0.000000000	-9.606825952
H	-5.769732039	-1.850934155	-5.079623411
H	-3.148941841	3.627354315	-3.850354088
H	-4.200656333	2.612879885	-4.848052057
H	-2.444031767	2.524725047	-5.027475452
H	-5.247289867	1.976442466	-2.552639115
H	-3.978743773	2.801745707	-1.631962580
H	-4.276945722	1.082096862	-1.381499048
H	-1.896784715	-4.128722537	-6.142177736
H	-0.180408830	-4.219210740	-5.750096373
H	-0.847820828	-2.744454124	-6.464044962
H	-1.709587307	-3.778882445	-2.356981562
H	-0.731164106	-4.845754156	-3.356864963
H	-2.474222037	-4.724968572	-3.644527739
H	0.000000000	0.000000000	9.770645888
O	-1.075994324	2.767050768	-0.936419074
C	-0.551436218	2.166640313	-0.015718891
O	0.704545733	2.237792794	0.312408139

References

1. G. R. Fulmer, A. J. M. Miller, N. H. Sherden, H. E. Gottlieb, A. Nudelman, B. M. Stoltz, J. E. Bercaw, K. I. Goldberg, *Organometallics* **2010**, *29*, 2176–2179.
2. N. K. T. Ho, B. Neumann, H.-G. Stammler, V. H. Menezes da Silva, D. G. Watanabe, A. A. C. Braga, R. S. Ghadwal, *Dalton Trans*. **2017**, *46*, 12027–12031.
3. R. Suter, Z. Benkő, M. Bispinghoff, H. Grützmacher, *Angew. Chem., Int. Ed.* **2017**, *56*, 11226–11231.
4. M. K. Sharma, D. Rottschäfer, T. Glodde, B. Neumann, H. G. Stammler, R. S. Ghadwal, *Angew. Chem. Int. Ed.* **2021**, *60*, 6414–6418.
5. D. Rottschäfer, F. Ebeler, T. Strothmann, B. Neumann, H.-G. Stammler, A. Mix, R. S. Ghadwal, *Chem. Eur. J.* **2018**, *24*, 3716–3720.
6. a) S. Bontemps, L. Vendier, S. Sabo-Etienne, *Angew. Chem., Int. Ed*. **2012**, *51*, 1671–1674; c) L. J. Murphy, H. Hollenhorst, R. McDonald, M. Ferguson, M. D. Lumsden, L. Turculet, *Organometallics* **2017**, *36*, 3709–3720; b) C.-C. Chia, Y.-C. Teo, N. Cham, S. Y.-F. Ho, Z.-H. Ng, H.-M. Toh, N. Mézailles, C.-W. So, *Inorg. Chem*. **2021**, *60*, 4569–4577.
7. O. V. Dolomanov, L. J. Bourhis, R. J. Gildea, J. A. K. Howard, H. Puschmann, *J. Appl. Crystallogr.* **2009**, *42*, 339–341.
8. G. M. Sheldrick, *Acta Crystallogr. A: Found. Adv.* **2015**, *71*, 3–8.
9. G. M. Sheldrick, *Acta Crystallogr. C: Struct. Chem.* **2015**, *71*, 3–8.
10. L. J. Bourhis, O. V. Dolomanov, R. J. Gildea, J. A. K. Howard, H. Puschmann, *Acta Crystallogr. A: Found. Adv*. **2015**, *71*, 59–75.
11. a) C. Adamo, V. Barone, *J. Chem. Phys.* **1999**, *110*, 6158–6170; b) S. Grimme, S. Ehrlich, L. Goerigk, *J. Comput. Chem.* **2011**, *32*, 1456–1465.
12. F. Neese, *Wiley Interdiscip. Rev. Comput. Mol. Sci*. **2012**, *2*, 73–78.
13. C. A. Bauer, A. Hansen, S. Grimme, *Chem. Eur. J.* **2017**, *23*, 6150–6164.
14. a) D. Doehnert, J. Koutecky, *J. Am. Chem. Soc.* **1980**, *102*, 1789–1796; b) K. Yamaguchi, M. Okumura, K. Takada, S. Yamanaka, *Int. J. Quantum Chem.* **1993**, *48*, 501–515.
15. NBO 6.0. E. D. Glendening, J. K. Badenhoop, A. E. Reed, J. E. Carpenter, J. A. Bohmann, C. M. Morales, C. R. Landis, and F. Weinhold (Theoretical Chemistry Institute, University of Wisconsin, Madison, WI, 2013), http://nbo6.chem.wisc.edu.
16. M. J. Frisch, G. W. Trucks, H. B. Schlegel, G. E. Scuseria, M. A. Robb, J. R. Cheeseman, G. Scalmani, V. Barone, G. A. Petersson, H. Nakatsuji, X. Li, M. Caricato, A. V. Marenich, J. Bloino, B. G. Janesko, R. Gomperts, B. Mennucci, H. P. Hratchian, J. V. Ortiz, A. F. Izmaylov, J. L. Sonnenberg, D. Williams-Young, F. Ding, F. Lipparini, F. Egidi, J. Goings, B. Peng, A. Petrone, T. Henderson, D. Ranasinghe, V. G. Zakrzewski, J. Gao, N. Rega, G. Zheng, W. Liang, M. Hada, M. Ehara, K. Toyota, R. Fukuda, J. Hasegawa, M. Ishida, T. Nakajima, Y. Honda, O. Kitao, H. Nakai, T. Vreven, K. Throssell, J. A. Montgomery, Jr., J. E. Peralta, F. Ogliaro, M. J.

Bearpark, J. J. Heyd, E. N. Brothers, K. N. Kudin, V. N. Staroverov, T. A. Keith, R. Kobayashi, J. Normand, K. Raghavachari, A. P. Rendell, J. C. Burant, S. S. Iyengar, J. Tomasi, M. Cossi, J. M. Millam, M. Klene, C. Adamo, R. Cammi, J. W. Ochterski, R. L. Martin, K. Morokuma, O. Farkas, J. B. Foresman, and D. J. Fox, Gaussian, Inc., Wallingford CT, 2016.

17. a) W. D. Laidig, H. F. Schaefer, *J. Chem. Phys*. **1981**, *74*, 3411–3414; b) E. Miliordos, K. Ruedenberg, S. S. Xantheas, *Angew. Chem., Int. Ed*. **2013**, *52*, 5736–5739.
