# Supplementary material for: Isolation of an Anionic Dicarbene Embedded Sn2P2 Cluster and Reversible CO2 Uptake
Source: Adv Sci (Weinh). 2023 Nov 28;11(5):2305545. doi: 10.1002/advs.202305545 (PMC10837339; doi:10.1002/advs.202305545)

checkCIF/PLATON report

Structure factors have been supplied for datablock(s) 2

THIS REPORT IS FOR GUIDANCE ONLY. IF USED AS PART OF A REVIEW PROCEDURE FOR PUBLICATION, IT SHOULD NOT REPLACE THE EXPERTISE OF AN EXPERIENCED CRYSTALLOGRAPHIC REFEREE.

No syntax errors found. CIF dictionary Interpreting this report

Datablock: 2

```
Bond precision:   C-C = 0.0099 A          Wavelength=0.71073

Cell:           a=14.4055(8)       b=14.7480(7)      c=18.9403(7)
                alpha=88.781(4)    beta=88.691(4)    gamma=85.613(4)
Temperature:    95 K
```

	Calculated	Reported
Volume	4010.3(3)	4010.3(3)
Space group	P -1	P -1
Hall group	-P 1	-P 1
Moiety formula	C68 H78 N4 O2 P2 Sn2 [+ solvent]	2(C34 H39 N2 O P Sn), 4[C4H8O]
Sum formula	C68 H78 N4 O2 P2 Sn2 [+ solvent]	C84 H110 N4 O6 P2 Sn2
Mr	1282.70	1571.07
Dx,g cm-3	1.062	1.301
Z	2	2
Mu (mm-1)	0.699	0.716
F000	1320.0	1640.0
F000'	1318.17	
h,k,lmax	20,20,26	20,20,26
Nref	23461	24743
Tmin,Tmax	0.799,0.899	0.267,1.000
Tmin'	0.778	

```
Correction method= # Reported T Limits: Tmin=0.267 Tmax=1.000
AbsCorr = GAUSSIAN

Data completeness= 1.055          Theta(max)= 30.034
```

R(reflections)= 0.0839(17513)

wR2(reflections)= 0.2163(24743)

S = 1.100   Npar= 720

The following ALERTS were generated. Each ALERT has the format
test-name_ALERT_alert-type_alert-level.
Click on the hyperlinks for more details of the test.

Alert level C

```
PLAT220_ALERT_2_C NonSolvent   Resd 1  C   Ueq(max)/Ueq(min) Range        4.2 Ratio
PLAT220_ALERT_2_C NonSolvent   Resd 2  C   Ueq(max)/Ueq(min) Range        4.0 Ratio
PLAT241_ALERT_2_C High   'MainMol' Ueq as Compared to Neighbors of         P1 Check
PLAT241_ALERT_2_C High   'MainMol' Ueq as Compared to Neighbors of         P2 Check
PLAT242_ALERT_2_C Low    'MainMol' Ueq as Compared to Neighbors of         C4 Check
PLAT242_ALERT_2_C Low    'MainMol' Ueq as Compared to Neighbors of        C38 Check
PLAT250_ALERT_2_C Large U3/U1 Ratio for Average U(i,j) Tensor ....        2.4 Note
PLAT250_ALERT_2_C Large U3/U1 Ratio for Average U(i,j) Tensor ....        2.1 Note
PLAT309_ALERT_2_C Single Bonded Oxygen (C-O > 1.3 Ang) ..........          O2 Check
PLAT342_ALERT_3_C Low Bond Precision on  C-C Bonds ..............     0.00987 Ang.
PLAT766_ALERT_4_C INS Embedded LIST 8 Instruction Should be LIST 4     Please Check
PLAT906_ALERT_3_C Large K Value in the Analysis of Variance ......      2.542 Check
PLAT911_ALERT_3_C Missing FCF Refl Between Thmin & STh/L=    0.600        321 Report
PLAT918_ALERT_3_C Reflection(s) with I(obs) much Smaller I(calc) .          3 Check
```

Alert level G

```
FORMU01_ALERT_2_G  There is a discrepancy between the atom counts in the
            _chemical_formula_sum and the formula from the _atom_site* data.
            Atom count from _chemical_formula_sum:C84 H110 N4 O6 P2 Sn2
            Atom count from the _atom_site data:  C68 H78 N4 O2 P2 Sn2
CELLZ01_ALERT_1_G Difference between formula and atom_site contents detected.
CELLZ01_ALERT_1_G ALERT: Large difference may be due to a
            symmetry error - see SYMMG tests
           From the CIF: _cell_formula_units_Z     2
           From the CIF: _chemical_formula_sum  C84 H110 N4 O6 P2 Sn2
           TEST: Compare cell contents of formula and atom_site data

           atom    Z*formula  cif sites diff
           C        168.00    136.00   32.00
           H        220.00    156.00   64.00
           N          8.00      8.00    0.00
           O         12.00      4.00    8.00
           P          4.00      4.00    0.00
           Sn         4.00      4.00    0.00
PLAT019_ALERT_1_G _diffrn_measured_fraction_theta_full/*_max < 1.0      0.993 Report
PLAT041_ALERT_1_G Calc. and Reported SumFormula    Strings  Differ     Please Check
PLAT042_ALERT_1_G Calc. and Reported MoietyFormula Strings  Differ     Please Check
PLAT083_ALERT_2_G SHELXL Second Parameter in WGHT  Unusually Large      26.83 Why ?
PLAT154_ALERT_1_G The s.u.'s on the Cell Angles are Equal ..(Note)      0.004 Degree
PLAT606_ALERT_4_G Solvent Accessible VOID(S) in Structure ........          ! Info
PLAT869_ALERT_4_G ALERTS Related to the Use of SQUEEZE  Suppressed          ! Info
PLAT870_ALERT_4_G ALERTS Related to Twinning Effects Suppressed ..          ! Info
PLAT910_ALERT_3_G Missing # of FCF Reflection(s) Below Theta(Min).          4 Note
PLAT912_ALERT_4_G Missing # of FCF Reflections Above STh/L=  0.600         26 Note
```

```
PLAT941_ALERT_3_G Average HKL Measurement Multiplicity ..........        1.1 Low
```

```
  0 ALERT level A = Most likely a serious problem - resolve or explain
  0 ALERT level B = A potentially serious problem, consider carefully
 14 ALERT level C = Check. Ensure it is not caused by an omission or oversight
 14 ALERT level G = General information/check it is not something unexpected

  6 ALERT type 1 CIF construction/syntax error, inconsistent or missing data
 11 ALERT type 2 Indicator that the structure model may be wrong or deficient
  6 ALERT type 3 Indicator that the structure quality may be low
  5 ALERT type 4 Improvement, methodology, query or suggestion
  0 ALERT type 5 Informative message, check
```

It is advisable to attempt to resolve as many as possible of the alerts in all categories. Often the minor alerts point to easily fixed oversights, errors and omissions in your CIF or refinement strategy, so attention to these fine details can be worthwhile. In order to resolve some of the more serious problems it may be necessary to carry out additional measurements or structure refinements. However, the purpose of your study may justify the reported deviations and the more serious of these should normally be commented upon in the discussion or experimental section of a paper or in the "special_details" fields of the CIF. checkCIF was carefully designed to identify outliers and unusual parameters, but every test has its limitations and alerts that are not important in a particular case may appear. Conversely, the absence of alerts does not guarantee there are no aspects of the results needing attention. It is up to the individual to critically assess their own results and, if necessary, seek expert advice.

Publication of your CIF in IUCr journals

A basic structural check has been run on your CIF. These basic checks will be run on all CIFs submitted for publication in IUCr journals (*Acta Crystallographica*, *Journal of Applied Crystallography*, *Journal of Synchrotron Radiation*); however, if you intend to submit to *Acta Crystallographica Section C* or *E* or *IUCrData*, you should make sure that full publication checks are run on the final version of your CIF prior to submission.

Publication of your CIF in other journals

Please refer to the *Notes for Authors* of the relevant journal for any special instructions relating to CIF submission.

PLATON version of 10/05/2023; check.def file version of 10/05/2023

Datablock 2 - ellipsoid plot

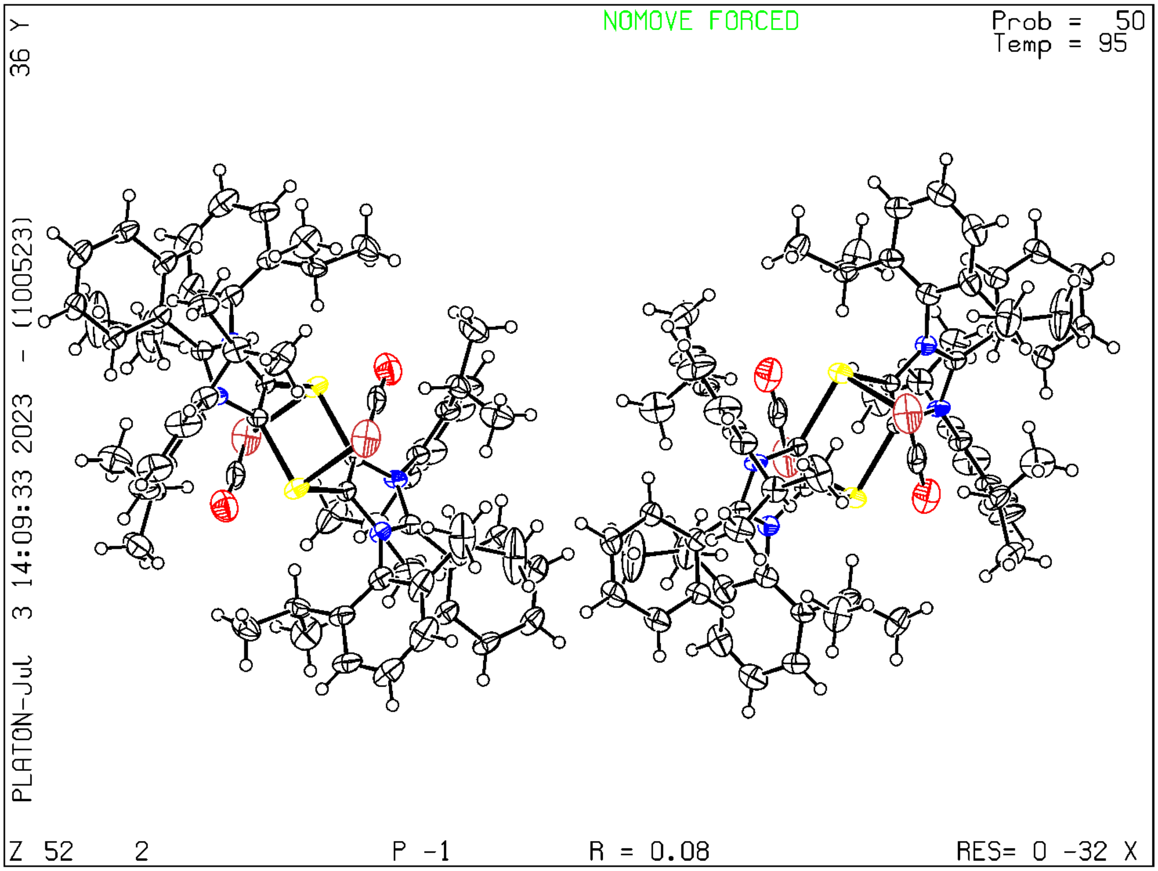

Supplement: Supplementary file 2 — Supporting Information [file ADVS-11-2305545-s002.zip › checkcif_2.pdf]
