# Supplementary material for: Isolation of an Anionic Dicarbene Embedded Sn2P2 Cluster and Reversible CO2 Uptake
Source: Adv Sci (Weinh). 2023 Nov 28;11(5):2305545. doi: 10.1002/advs.202305545 (PMC10837339; doi:10.1002/advs.202305545)

checkCIF/PLATON report

Structure factors have been supplied for datablock(s) 3a_3b

THIS REPORT IS FOR GUIDANCE ONLY. IF USED AS PART OF A REVIEW PROCEDURE FOR PUBLICATION, IT SHOULD NOT REPLACE THE EXPERTISE OF AN EXPERIENCED CRYSTALLOGRAPHIC REFEREE.

No syntax errors found. CIF dictionary Interpreting this report

Datablock: 3a_3b

Bond precision:	C-C = 0.0117 A		Wavelength=0.71073
Cell:	a=28.9585(6)	b=10.5653(2)	c=28.7219(7)
	alpha=90	beta=92.300(2)	gamma=90
Temperature:	100 K		

	Calculated	Reported
Volume	8780.5(3)	8780.5(3)
Space group	I 2	I 1 2 1
Hall group	I 2y	I 2y
Moiety formula	C68 H78 N4 O2 P2 Sn2, 4(C6 H6) [+ solvent]	2(C34 H39 N2 O P Sn), 4(C6 H6), 1[C6H6]
Sum formula	C92 H102 N4 O2 P2 Sn2 [+ solvent]	C98 H108 N4 O2 P2 Sn2
Mr	1595.14	1673.20
Dx,g cm-3	1.207	1.266
Z	4	4
Mu (mm-1)	0.652	0.655
F000	3312.0	3480.0
F000’	3308.57	
h,k,lmax	39,14,39	38,13,39
Nref	23267[12241]	19866
Tmin,Tmax	0.939,0.955	0.756,1.000
Tmin’	0.770	

Correction method= # Reported T Limits: Tmin=0.756 Tmax=1.000
AbsCorr = GAUSSIAN

Data completeness= 1.62/0.85 Theta(max)= 28.953

R(reflections)= 0.0477(17549) wR2(reflections)= 0.1100(19866)

S = 1.032 Npar= 1047

The following ALERTS were generated. Each ALERT has the format
test-name_ALERT_alert-type_alert-level.
Click on the hyperlinks for more details of the test.

Alert level B

PLAT910_ALERT_3_B Missing # of FCF Reflection(s) Below Theta(Min). 18 Note

---

Alert level C

PLAT220_ALERT_2_C NonSolvent Resd 1 C Ueq(max)/Ueq(min) Range 3.1 Ratio
PLAT234_ALERT_4_C Large Hirshfeld Difference C44B --C45B . 0.18 Ang.
PLAT234_ALERT_4_C Large Hirshfeld Difference C56 --C57B . 0.19 Ang.
PLAT234_ALERT_4_C Large Hirshfeld Difference C13 --C14 . 0.17 Ang.
PLAT242_ALERT_2_C Low 'MainMol' Ueq as Compared to Neighbors of C13 Check
PLAT244_ALERT_4_C Low 'Solvent' Ueq as Compared to Neighbors of C77 Check
PLAT250_ALERT_2_C Large U3/U1 Ratio for Average U(i,j) Tensor .... 2.1 Note
PLAT250_ALERT_2_C Large U3/U1 Ratio for Average U(i,j) Tensor .... 2.5 Note
PLAT331_ALERT_2_C Small Aver Phenyl C-C Dist C69 --C74 . 1.37 Ang.
PLAT331_ALERT_2_C Small Aver Phenyl C-C Dist C75 --C80 . 1.36 Ang.
PLAT342_ALERT_3_C Low Bond Precision on C-C Bonds .... 0.01172 Ang.
PLAT911_ALERT_3_C Missing FCF Refl Between Thmin & STh/L= 0.600 5 Report
PLAT913_ALERT_3_C Missing # of Very Strong Reflections in FCF .... 4 Note
PLAT915_ALERT_3_C No Flack x Check Done: Low Friedel Pair Coverage 79 %

Alert level G

FORMU01_ALERT_2_G There is a discrepancy between the atom counts in the
_chemical_formula_sum and the formula from the _atom_site* data.
Atom count from _chemical_formula_sum:C98 H108 N4 O2 P2 Sn2
Atom count from the _atom_site data: C92 H102 N4 O2 P2 Sn2
CELLZ01_ALERT_1_G Difference between formula and atom_site contents detected.
CELLZ01_ALERT_1_G ALERT: Large difference may be due to a
symmetry error - see SYMMG tests
From the CIF: _cell_formula_units_Z 4
From the CIF: _chemical_formula_sum C98 H108 N4 O2 P2 Sn2
TEST: Compare cell contents of formula and atom_site data

atom	Z*formula	cif sites	diff
C	392.00	368.00	24.00
H	432.00	408.00	24.00
N	16.00	16.00	0.00
O	8.00	8.00	0.00
P	8.00	8.00	0.00
Sn	8.00	8.00	0.00

PLAT002_ALERT_2_G Number of Distance or Angle Restraints on AtSite 4 Note
PLAT003_ALERT_2_G Number of Uiso or Uij Restrained non-H Atoms ... 15 Report
PLAT041_ALERT_1_G Calc. and Reported SumFormula Strings Differ Please Check
PLAT042_ALERT_1_G Calc. and Reported MoietyFormula Strings Differ Please Check
PLAT083_ALERT_2_G SHELXL Second Parameter in WGHT Unusually Large 16.21 Why ?
PLAT158_ALERT_4_G The Input Unitcell is NOT Standard/Reduced ..... Please Check

```
PLAT171_ALERT_4_G The CIF-Embedded .res File Contains EADP Records          7 Report
PLAT176_ALERT_4_G The CIF-Embedded .res File Contains SADI Records          1 Report
PLAT178_ALERT_4_G The CIF-Embedded .res File Contains SIMU Records          3 Report
PLAT187_ALERT_4_G The CIF-Embedded .res File Contains RIGU Records          2 Report
PLAT188_ALERT_3_G A Non-default SIMU Restraint Value has been used     0.0010 Report
PLAT188_ALERT_3_G A Non-default SIMU Restraint Value has been used     0.0100 Report
PLAT188_ALERT_3_G A Non-default SIMU Restraint Value has been used     0.0010 Report
PLAT190_ALERT_3_G A Non-default RIGU Restraint Value for First Par     0.0010 Report
PLAT190_ALERT_3_G A Non-default RIGU Restraint Value for SecondPar     0.0010 Report
PLAT190_ALERT_3_G A Non-default RIGU Restraint Value for First Par     0.0010 Report
PLAT190_ALERT_3_G A Non-default RIGU Restraint Value for SecondPar     0.0010 Report
PLAT301_ALERT_3_G Main Residue  Disorder .............(Resd  1  )        27% Note
PLAT302_ALERT_4_G Anion/Solvent/Minor-Residue Disorder (Resd  2  )        21% Note
PLAT328_ALERT_4_G Possible Missing H on sp3? Phosphorus .........         P3B Check
PLAT328_ALERT_4_G Possible Missing H on sp3? Phosphorus .........         P1A Check
PLAT411_ALERT_2_G Short Inter H...H Contact  H53      ..H32B     .       2.09 Ang.
                                     3/2-x,-1/2+y,1/2-z  =      4_645 Check
PLAT411_ALERT_2_G Short Inter H...H Contact  H65      ..H30B     .       1.66 Ang.
                                                x,y,z  =      1_555 Check
PLAT412_ALERT_2_G Short Intra XH3 .. XHn     H42      ..H48F     .       2.07 Ang.
                                                x,y,z  =      1_555 Check
PLAT412_ALERT_2_G Short Intra XH3 .. XHn     H52      ..H57C     .       1.80 Ang.
                                                x,y,z  =      1_555 Check
PLAT412_ALERT_2_G Short Intra XH3 .. XHn     H54      ..H61F     .       1.76 Ang.
                                                x,y,z  =      1_555 Check
PLAT412_ALERT_2_G Short Intra XH3 .. XHn     H57C     ..H58A     .       2.01 Ang.
                                                x,y,z  =      1_555 Check
PLAT412_ALERT_2_G Short Intra XH3 .. XHn     H60A     ..H61F     .       1.89 Ang.
                                                x,y,z  =      1_555 Check
PLAT412_ALERT_2_G Short Intra XH3 .. XHn     H61A     ..H67      .       1.83 Ang.
                                                x,y,z  =      1_555 Check
PLAT412_ALERT_2_G Short Intra XH3 .. XHn     H63      ..H49F     .       1.94 Ang.
                                                x,y,z  =      1_555 Check
PLAT412_ALERT_2_G Short Intra XH3 .. XHn     H12C     ..H29B     .       1.69 Ang.
                                                x,y,z  =      1_555 Check
PLAT412_ALERT_2_G Short Intra XH3 .. XHn     H26B     ..H33B     .       2.06 Ang.
                                                x,y,z  =      1_555 Check
PLAT413_ALERT_2_G Short Inter XH3 .. XHn     H57B     ..H60C     .       2.06 Ang.
                                              x,1+y,z  =      1_565 Check
PLAT432_ALERT_2_G Short Inter X...Y Contact  C53      ..C32B     .       3.09 Ang.
                                     3/2-x,-1/2+y,1/2-z  =      4_645 Check
PLAT432_ALERT_2_G Short Inter X...Y Contact  C54      ..C32B     .       3.12 Ang.
                                     3/2-x,-1/2+y,1/2-z  =      4_645 Check
PLAT432_ALERT_2_G Short Inter X...Y Contact  C65      ..C30B     .       3.02 Ang.
                                                x,y,z  =      1_555 Check
PLAT606_ALERT_4_G Solvent Accessible VOID(S) in Structure ........          ! Info
PLAT802_ALERT_4_G CIF Input Record(s) with more than 80 Characters          2 Info
PLAT860_ALERT_3_G Number of Least-Squares Restraints ............         134 Note
PLAT868_ALERT_4_G ALERTS Due to the Use of _smtbx_masks Suppressed          ! Info
PLAT912_ALERT_4_G Missing # of FCF Reflections Above STh/L=  0.600       1030 Note
PLAT933_ALERT_2_G Number of HKL-OMIT Records in Embedded .res File          1 Note
PLAT978_ALERT_2_G Number C-C Bonds with Positive Residual Density.          0 Info
```

---

0 **ALERT level A** = Most likely a serious problem - resolve or explain
1 **ALERT level B** = A potentially serious problem, consider carefully
14 **ALERT level C** = Check. Ensure it is not caused by an omission or oversight

```
46 ALERT level G = General information/check it is not something unexpected

 4 ALERT type 1 CIF construction/syntax error, inconsistent or missing data
27 ALERT type 2 Indicator that the structure model may be wrong or deficient
14 ALERT type 3 Indicator that the structure quality may be low
16 ALERT type 4 Improvement, methodology, query or suggestion
 0 ALERT type 5 Informative message, check
```

It is advisable to attempt to resolve as many as possible of the alerts in all categories. Often the minor alerts point to easily fixed oversights, errors and omissions in your CIF or refinement strategy, so attention to these fine details can be worthwhile. In order to resolve some of the more serious problems it may be necessary to carry out additional measurements or structure refinements. However, the purpose of your study may justify the reported deviations and the more serious of these should normally be commented upon in the discussion or experimental section of a paper or in the "special_details" fields of the CIF. checkCIF was carefully designed to identify outliers and unusual parameters, but every test has its limitations and alerts that are not important in a particular case may appear. Conversely, the absence of alerts does not guarantee there are no aspects of the results needing attention. It is up to the individual to critically assess their own results and, if necessary, seek expert advice.

Publication of your CIF in IUCr journals

A basic structural check has been run on your CIF. These basic checks will be run on all CIFs submitted for publication in IUCr journals (*Acta Crystallographica*, *Journal of Applied Crystallography*, *Journal of Synchrotron Radiation*); however, if you intend to submit to *Acta Crystallographica Section C* or *E* or *IUCrData*, you should make sure that full publication checks are run on the final version of your CIF prior to submission.

Publication of your CIF in other journals

Please refer to the *Notes for Authors* of the relevant journal for any special instructions relating to CIF submission.

PLATON version of 10/05/2023; check.def file version of 10/05/2023

Datablock 3a_3b - ellipsoid plot

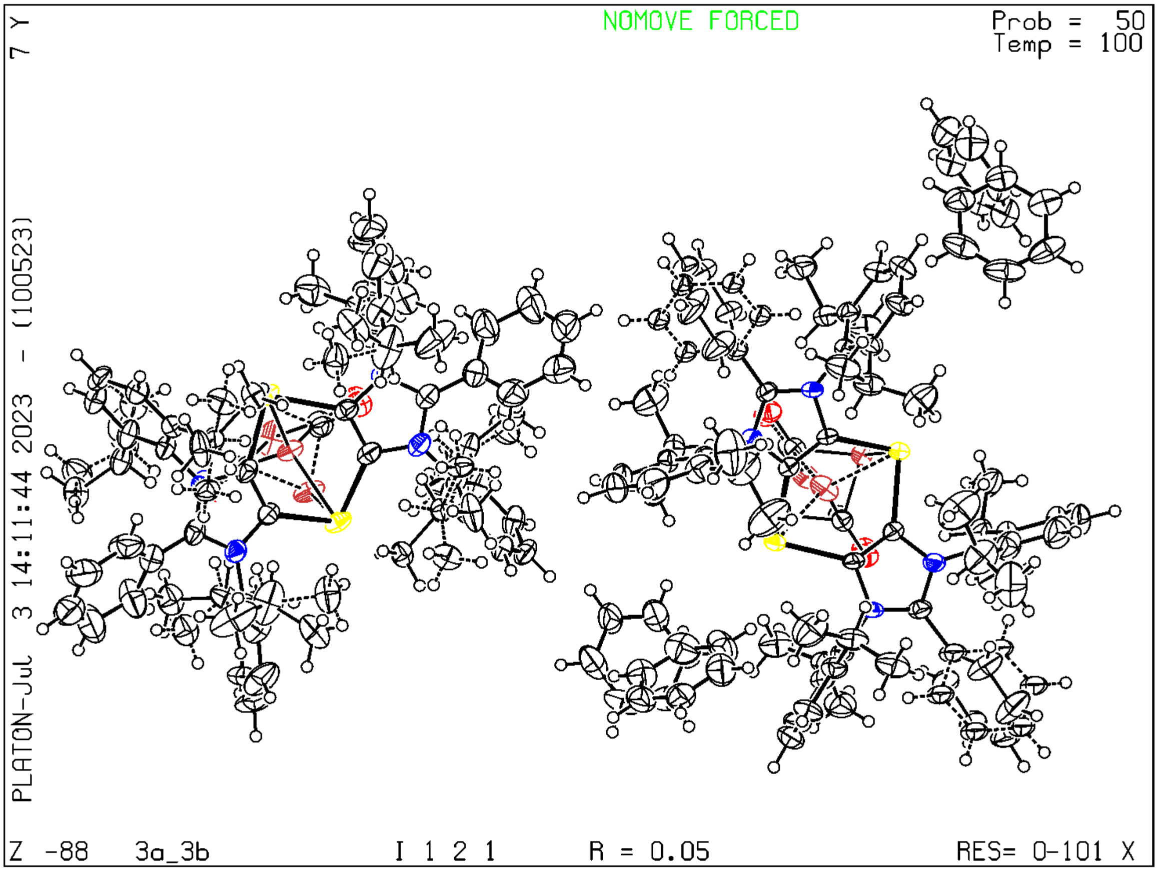

Supplement: Supplementary file 2 — Supporting Information [file ADVS-11-2305545-s002.zip › checkcif_3a_3b.pdf]
