# Supplementary material for: Isolation of an Anionic Dicarbene Embedded Sn2P2 Cluster and Reversible CO2 Uptake
Source: Adv Sci (Weinh). 2023 Nov 28;11(5):2305545. doi: 10.1002/advs.202305545 (PMC10837339; doi:10.1002/advs.202305545)

checkCIF/PLATON report

Structure factors have been supplied for datablock(s) 5

THIS REPORT IS FOR GUIDANCE ONLY. IF USED AS PART OF A REVIEW PROCEDURE FOR PUBLICATION, IT SHOULD NOT REPLACE THE EXPERTISE OF AN EXPERIENCED CRYSTALLOGRAPHIC REFEREE.

No syntax errors found. CIF dictionary Interpreting this report

Datablock: 5

Bond precision:	C-C = 0.0060 A		Wavelength=0.71073
Cell:	a=17.8067(2)	b=17.0832(3)	c=23.8311(4)
	alpha=90	beta=93.578(1)	gamma=90
Temperature:	100 K		

	Calculated	Reported
Volume	7235.18(19)	7235.18(19)
Space group	P 21/c	P 1 21/c 1
Hall group	-P 2ybc	-P 2ybc
Moiety formula	C66 H78 N4 P2 Sn2, 1.5(C6 H6) [+ solvent]	C66 H78 N4 P2 Sn2, 1.5(C6 H6), 0.5[C6H6]
Sum formula	C75 H87 N4 P2 Sn2 [+ solvent]	C78 H90 N4 P2 Sn2
Mr	1343.85	1382.85
Dx,g cm-3	1.234	1.270
Z	4	4
Mu (mm-1)	0.776	0.778
F000	2780.0	2864.0
F000'	2776.33	
h,k,lmax	26,25,35	26,25,35
Nref	25791	24471
Tmin,Tmax	0.751,0.962	0.457,1.000
Tmin'	0.737	

Correction method= # Reported T Limits: Tmin=0.457 Tmax=1.000
AbsCorr = GAUSSIAN

Data completeness= 0.949 Theta(max)= 32.315

R(reflections)= 0.0686(20496)   wR2(reflections)= 0.1537(24471)

S = 1.226   Npar= 883

The following ALERTS were generated. Each ALERT has the format
test-name_ALERT_alert-type_alert-level.
Click on the hyperlinks for more details of the test.

Alert level C

```
PLAT213_ALERT_2_C Atom C37             has ADP max/min Ratio .....        3.5 prolat
PLAT213_ALERT_2_C Atom C37B            has ADP max/min Ratio .....        3.5 prolat
PLAT220_ALERT_2_C NonSolvent   Resd 1  C   Ueq(max)/Ueq(min) Range        4.1 Ratio
PLAT222_ALERT_3_C NonSolvent Resd 1  H   Uiso(max)/Uiso(min) Range        4.3 Ratio
PLAT250_ALERT_2_C Large U3/U1 Ratio for Average U(i,j) Tensor ....        3.6 Note
PLAT334_ALERT_2_C Small <C-C> Benzene Dist.   C37B     -C42B     .       1.37 Ang.
PLAT906_ALERT_3_C Large K Value in the Analysis of Variance ......      6.627 Check
PLAT906_ALERT_3_C Large K Value in the Analysis of Variance ......      2.349 Check
PLAT911_ALERT_3_C Missing FCF Refl Between Thmin & STh/L=    0.600          5 Report
PLAT977_ALERT_2_C Check Negative Difference Density on H74       .      -0.35 eA-3
```

Alert level G

```
FORMU01_ALERT_2_G  There is a discrepancy between the atom counts in the
            _chemical_formula_sum and the formula from the _atom_site* data.
            Atom count from _chemical_formula_sum:C78 H90 N4 P2 Sn2
            Atom count from the _atom_site data:  C75 H87 N4 P2 Sn2
CELLZ01_ALERT_1_G Difference between formula and atom_site contents detected.
CELLZ01_ALERT_1_G ALERT: Large difference may be due to a
            symmetry error - see SYMMG tests
           From the CIF: _cell_formula_units_Z    4
           From the CIF: _chemical_formula_sum  C78 H90 N4 P2 Sn2
           TEST: Compare cell contents of formula and atom_site data

           atom    Z*formula  cif sites diff
           C        312.00    300.00   12.00
           H        360.00    348.00   12.00
           N         16.00     16.00    0.00
           P          8.00      8.00    0.00
           Sn         8.00      8.00    0.00
PLAT041_ALERT_1_G Calc. and Reported SumFormula    Strings  Differ     Please Check
PLAT083_ALERT_2_G SHELXL Second Parameter in WGHT  Unusually Large      23.99 Why ?
PLAT171_ALERT_4_G The CIF-Embedded .res File Contains EADP Records          7 Report
PLAT230_ALERT_2_G Hirshfeld Test Diff for    P2B      --C3       .        7.7 s.u.
PLAT230_ALERT_2_G Hirshfeld Test Diff for    C37      --C42      .        6.0 s.u.
PLAT230_ALERT_2_G Hirshfeld Test Diff for    C55      --C56A     .        6.2 s.u.
PLAT301_ALERT_3_G Main Residue  Disorder ..............(Resd  1  )        23% Note
PLAT328_ALERT_4_G Possible Missing H on sp3? Phosphorus ..........        P1A Check
PLAT328_ALERT_4_G Possible Missing H on sp3? Phosphorus ..........        P2A Check
PLAT328_ALERT_4_G Possible Missing H on sp3? Phosphorus ..........        P1B Check
PLAT328_ALERT_4_G Possible Missing H on sp3? Phosphorus ..........        P2B Check
PLAT335_ALERT_2_G Check Large C6 Ring C-C Range C37      -C42            0.18 Ang.
PLAT335_ALERT_2_G Check Large C6 Ring C-C Range C37B     -C42B           0.77 Ang.
PLAT412_ALERT_2_G Short Intra XH3 .. XHn     H57A     ..H55A     .       1.92 Ang.
                                                      x,y,z  =      1_555 Check
```

```
PLAT412_ALERT_2_G Short Intra XH3 .. XHn     H57B     ..H56E     .       1.91 Ang.
                                                     x,y,z  =      1_555 Check
PLAT605_ALERT_4_G Largest Solvent Accessible VOID in the Structure        340 A**3
PLAT773_ALERT_2_G Check long C-C Bond in CIF: C37B     --C38B          1.73 Ang.
PLAT790_ALERT_4_G Centre of Gravity not Within Unit Cell: Resd.  #          2 Note
              C6 H6
PLAT802_ALERT_4_G CIF Input Record(s) with more than 80 Characters          2 Info
PLAT868_ALERT_4_G ALERTS Due to the Use of _smtbx_masks Suppressed          ! Info
PLAT910_ALERT_3_G Missing # of FCF Reflection(s) Below Theta(Min).          2 Note
PLAT912_ALERT_4_G Missing # of FCF Reflections Above STh/L=  0.600       1314 Note
PLAT933_ALERT_2_G Number of HKL-OMIT Records in Embedded .res File          5 Note
PLAT978_ALERT_2_G Number C-C Bonds with Positive Residual Density.          2 Info
```

```
   0 ALERT level A = Most likely a serious problem - resolve or explain
   0 ALERT level B = A potentially serious problem, consider carefully
  10 ALERT level C = Check. Ensure it is not caused by an omission or oversight
  27 ALERT level G = General information/check it is not something unexpected

   3 ALERT type 1 CIF construction/syntax error, inconsistent or missing data
  18 ALERT type 2 Indicator that the structure model may be wrong or deficient
   6 ALERT type 3 Indicator that the structure quality may be low
  10 ALERT type 4 Improvement, methodology, query or suggestion
   0 ALERT type 5 Informative message, check
```

It is advisable to attempt to resolve as many as possible of the alerts in all categories. Often the minor alerts point to easily fixed oversights, errors and omissions in your CIF or refinement strategy, so attention to these fine details can be worthwhile. In order to resolve some of the more serious problems it may be necessary to carry out additional measurements or structure refinements. However, the purpose of your study may justify the reported deviations and the more serious of these should normally be commented upon in the discussion or experimental section of a paper or in the "special_details" fields of the CIF. checkCIF was carefully designed to identify outliers and unusual parameters, but every test has its limitations and alerts that are not important in a particular case may appear. Conversely, the absence of alerts does not guarantee there are no aspects of the results needing attention. It is up to the individual to critically assess their own results and, if necessary, seek expert advice.

Publication of your CIF in IUCr journals

A basic structural check has been run on your CIF. These basic checks will be run on all CIFs submitted for publication in IUCr journals (*Acta Crystallographica*, *Journal of Applied Crystallography*, *Journal of Synchrotron Radiation*); however, if you intend to submit to *Acta Crystallographica Section C* or *E* or *IUCrData*, you should make sure that full publication checks are run on the final version of your CIF prior to submission.

Publication of your CIF in other journals

Please refer to the *Notes for Authors* of the relevant journal for any special instructions relating to CIF submission.

PLATON version of 10/05/2023; check.def file version of 10/05/2023

Datablock 5 - ellipsoid plot

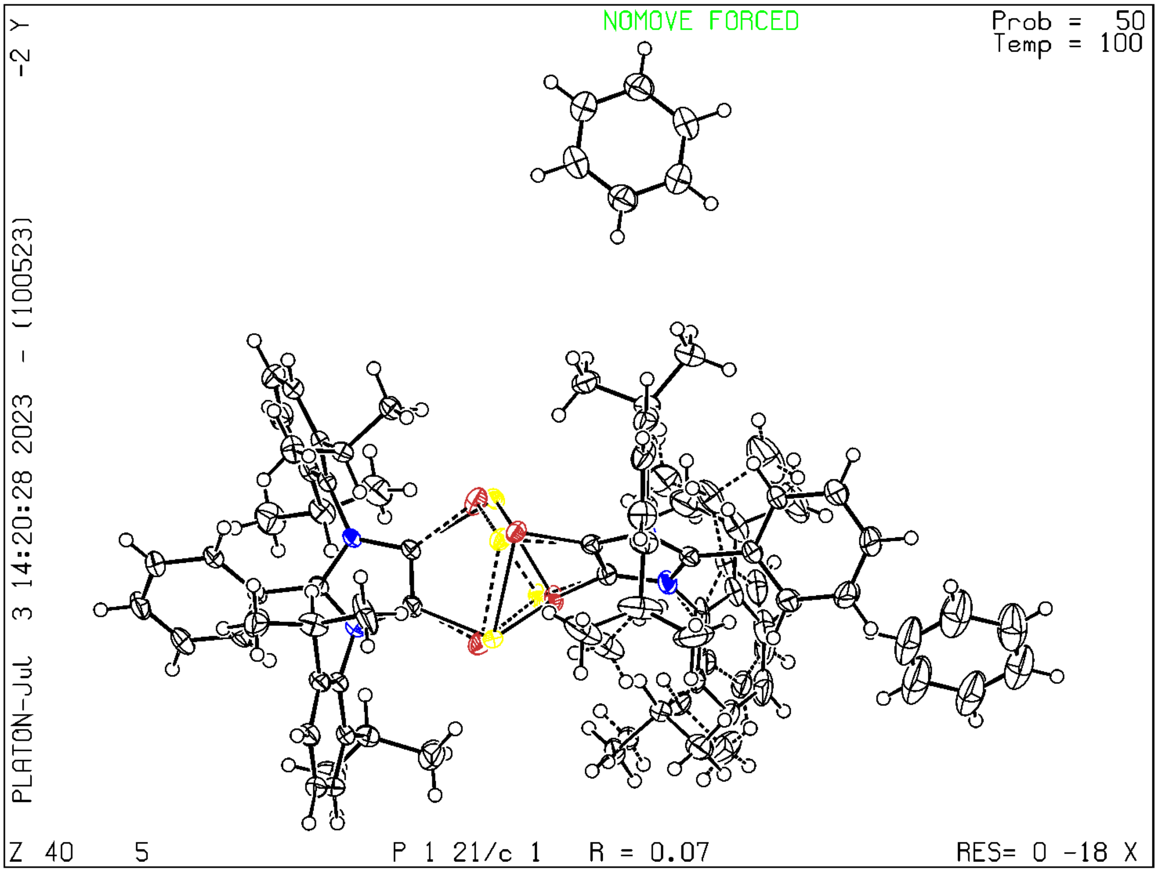

Supplement: Supplementary file 2 — Supporting Information [file ADVS-11-2305545-s002.zip › checkcif_5.pdf]
