# Supplementary material for: Isolation of an Anionic Dicarbene Embedded Sn2P2 Cluster and Reversible CO2 Uptake
Source: Adv Sci (Weinh). 2023 Nov 28;11(5):2305545. doi: 10.1002/advs.202305545 (PMC10837339; doi:10.1002/advs.202305545)

checkCIF/PLATON report

Structure factors have been supplied for datablock(s) 6

THIS REPORT IS FOR GUIDANCE ONLY. IF USED AS PART OF A REVIEW PROCEDURE FOR PUBLICATION, IT SHOULD NOT REPLACE THE EXPERTISE OF AN EXPERIENCED CRYSTALLOGRAPHIC REFEREE.

No syntax errors found. CIF dictionary Interpreting this report

Datablock: 6

Bond precision:	C-C = 0.0096 A		Wavelength=1.54184
Cell:	a=16.1913(3)	b=19.0091(3)	c=24.5120(5)
	alpha=90	beta=92.276(2)	gamma=90
Temperature:	100 K		

	Calculated	Reported
Volume	7538.4(2)	7538.4(2)
Space group	P 21/n	P 1 21/n 1
Hall group	-P 2yn	-P 2yn
Moiety formula	C66.89 H78 N4 O1.77 P2 Sn2, 2(C7 H8)	C66.89 H78 N4 O1.77 P2 Sn2, 2(C7 H8)
Sum formula	C80.89 H94 N4 O1.77 P2 Sn2	C80.89 H94 N4 O1.77 P2 Sn2
Mr	1450.01	1449.97
Dx,g cm-3	1.278	1.278
Z	4	4
Mu (mm-1)	6.024	6.024
F000	3006.1	3006.0
F000'	3015.38	
h,k,lmax	20,23,30	20,23,30
Nref	15854	15216
Tmin,Tmax	0.589,0.835	0.678,0.910
Tmin'	0.521	

Correction method= # Reported T Limits: Tmin=0.678 Tmax=0.910
AbsCorr = GAUSSIAN

Data completeness= 0.960 Theta(max)= 76.554

R(reflections)= 0.0587(12463) wR2(reflections)= 0.1556(15216)

S = 1.014 Npar= 884

The following ALERTS were generated. Each ALERT has the format
test-name_ALERT_alert-type_alert-level.
Click on the hyperlinks for more details of the test.

Alert level C

```
PLAT077_ALERT_4_C Unitcell Contains Non-integer Number of Atoms ..     Please Check
PLAT220_ALERT_2_C NonSolvent   Resd 1  C   Ueq(max)/Ueq(min) Range        5.0 Ratio
PLAT222_ALERT_3_C NonSolvent Resd 1  H   Uiso(max)/Uiso(min) Range        4.3 Ratio
PLAT234_ALERT_4_C Large Hirshfeld Difference C79A     --C80A     .       0.16 Ang.
PLAT242_ALERT_2_C Low    'MainMol' Ueq as Compared to Neighbors of        C13 Check
PLAT244_ALERT_4_C Low    'Solvent' Ueq as Compared to Neighbors of        C72 Check
PLAT250_ALERT_2_C Large U3/U1 Ratio for Average U(i,j) Tensor ....        2.3 Note
PLAT342_ALERT_3_C Low Bond Precision on  C-C Bonds ..............     0.00965 Ang.
PLAT601_ALERT_2_C Unit Cell Contains Solvent Accessible VOIDS of .         36 Ang**3
PLAT906_ALERT_3_C Large K Value in the Analysis of Variance ......      2.720 Check
PLAT911_ALERT_3_C Missing FCF Refl Between Thmin & STh/L=    0.600        168 Report
PLAT972_ALERT_2_C Check Calcd Resid. Dens.  0.64Ang From Sn2B           -1.84 eA-3
PLAT972_ALERT_2_C Check Calcd Resid. Dens.  0.98Ang From Sn1            -1.67 eA-3
PLAT977_ALERT_2_C Check Negative Difference Density on H80C      .      -0.32 eA-3
```

Alert level G

```
PLAT002_ALERT_2_G Number of Distance or Angle Restraints on AtSite         14 Note
PLAT003_ALERT_2_G Number of Uiso or Uij Restrained non-H Atoms ...         14 Report
PLAT083_ALERT_2_G SHELXL Second Parameter in WGHT  Unusually Large      24.64 Why ?
PLAT171_ALERT_4_G The CIF-Embedded .res File Contains EADP Records          4 Report
PLAT174_ALERT_4_G The CIF-Embedded .res File Contains FLAT Records          2 Report
PLAT176_ALERT_4_G The CIF-Embedded .res File Contains SADI Records          6 Report
PLAT178_ALERT_4_G The CIF-Embedded .res File Contains SIMU Records          2 Report
PLAT187_ALERT_4_G The CIF-Embedded .res File Contains RIGU Records          2 Report
PLAT191_ALERT_3_G A Non-default SADI Restraint Value has been used     0.0400 Report
PLAT191_ALERT_3_G A Non-default SADI Restraint Value has been used     0.0400 Report
PLAT191_ALERT_3_G A Non-default SADI Restraint Value has been used     0.0400 Report
PLAT191_ALERT_3_G A Non-default SADI Restraint Value has been used     0.0400 Report
PLAT232_ALERT_2_G Hirshfeld Test Diff (M-X)  Sn1      --P2A      .        6.7 s.u.
PLAT232_ALERT_2_G Hirshfeld Test Diff (M-X)  Sn1      --P2B      .        9.8 s.u.
PLAT301_ALERT_3_G Main Residue  Disorder .............(Resd  1  )          6% Note
PLAT302_ALERT_4_G Anion/Solvent/Minor-Residue Disorder (Resd  3  )       100% Note
PLAT302_ALERT_4_G Anion/Solvent/Minor-Residue Disorder (Resd  4  )       100% Note
PLAT304_ALERT_4_G Non-Integer Number of Atoms in ..... (Resd  1  )     154.66 Check
PLAT304_ALERT_4_G Non-Integer Number of Atoms in ..... (Resd  3  )      11.69 Check
PLAT304_ALERT_4_G Non-Integer Number of Atoms in ..... (Resd  4  )       3.31 Check
PLAT328_ALERT_4_G Possible Missing H on sp3? Phosphorus ..........         P1 Check
PLAT328_ALERT_4_G Possible Missing H on sp3? Phosphorus ..........        P2A Check
PLAT328_ALERT_4_G Possible Missing H on sp3? Phosphorus ..........        P2B Check
PLAT380_ALERT_4_G Incorrectly? Oriented X(sp2)-Methyl Moiety .....       C80A Check
PLAT722_ALERT_1_G Angle   Calc      108.00, Rep      109.50 Dev...       1.50 Degree
              C79B    -C80B    -H80F       1_555  1_555  1_555     #  381 Check
PLAT722_ALERT_1_G Angle   Calc      111.00, Rep      109.50 Dev...       1.50 Degree
              H80D    -C80B    -H80E       1_555  1_555  1_555     #  382 Check
PLAT860_ALERT_3_G Number of Least-Squares Restraints .............        244 Note
PLAT912_ALERT_4_G Missing # of FCF Reflections Above STh/L=  0.600        471 Note
PLAT941_ALERT_3_G Average HKL Measurement Multiplicity ...........        3.2 Low
PLAT978_ALERT_2_G Number C-C Bonds with Positive Residual Density.          0 Info
```

```
 0 ALERT level A = Most likely a serious problem - resolve or explain
 0 ALERT level B = A potentially serious problem, consider carefully
14 ALERT level C = Check. Ensure it is not caused by an omission or oversight
30 ALERT level G = General information/check it is not something unexpected

 2 ALERT type 1 CIF construction/syntax error, inconsistent or missing data
13 ALERT type 2 Indicator that the structure model may be wrong or deficient
11 ALERT type 3 Indicator that the structure quality may be low
18 ALERT type 4 Improvement, methodology, query or suggestion
 0 ALERT type 5 Informative message, check
```

It is advisable to attempt to resolve as many as possible of the alerts in all categories. Often the minor alerts point to easily fixed oversights, errors and omissions in your CIF or refinement strategy, so attention to these fine details can be worthwhile. In order to resolve some of the more serious problems it may be necessary to carry out additional measurements or structure refinements. However, the purpose of your study may justify the reported deviations and the more serious of these should normally be commented upon in the discussion or experimental section of a paper or in the "special_details" fields of the CIF. checkCIF was carefully designed to identify outliers and unusual parameters, but every test has its limitations and alerts that are not important in a particular case may appear. Conversely, the absence of alerts does not guarantee there are no aspects of the results needing attention. It is up to the individual to critically assess their own results and, if necessary, seek expert advice.

Publication of your CIF in IUCr journals

A basic structural check has been run on your CIF. These basic checks will be run on all CIFs submitted for publication in IUCr journals (*Acta Crystallographica*, *Journal of Applied Crystallography*, *Journal of Synchrotron Radiation*); however, if you intend to submit to *Acta Crystallographica Section C* or *E* or *IUCrData*, you should make sure that full publication checks are run on the final version of your CIF prior to submission.

Publication of your CIF in other journals

Please refer to the *Notes for Authors* of the relevant journal for any special instructions relating to CIF submission.

PLATON version of 10/05/2023; check.def file version of 10/05/2023

Datablock 6 - ellipsoid plot

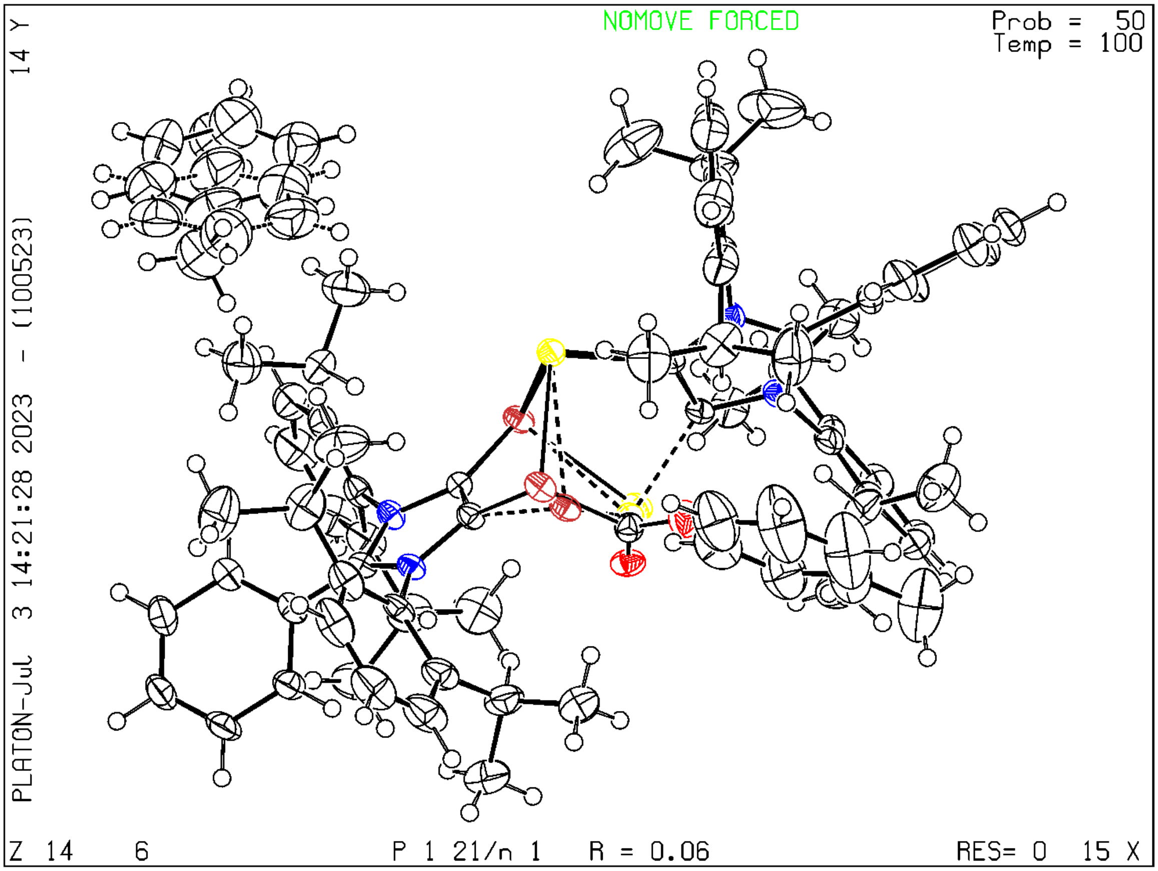

Supplement: Supplementary file 2 — Supporting Information [file ADVS-11-2305545-s002.zip › checkcif_6.pdf]
