# Supplementary material for: Isolation of an Anionic Dicarbene Embedded Sn2P2 Cluster and Reversible CO2 Uptake
Source: Adv Sci (Weinh). 2023 Nov 28;11(5):2305545. doi: 10.1002/advs.202305545 (PMC10837339; doi:10.1002/advs.202305545)

checkCIF/PLATON report

Structure factors have been supplied for datablock(s) 7

THIS REPORT IS FOR GUIDANCE ONLY. IF USED AS PART OF A REVIEW PROCEDURE FOR PUBLICATION, IT SHOULD NOT REPLACE THE EXPERTISE OF AN EXPERIENCED CRYSTALLOGRAPHIC REFEREE.

No syntax errors found. CIF dictionary Interpreting this report

Datablock: 7

Bond precision: C-C = 0.0052 A Wavelength=0.71073

Cell:	a=16.6805(5)	b=21.8588(6)	c=19.7261(6)
	alpha=90	beta=104.185(3)	gamma=90
Temperature:	100 K		

	Calculated	Reported
Volume	6973.2(4)	6973.1(4)
Space group	P 21/c	P 1 21/c 1
Hall group	-P 2ybc	-P 2ybc
Moiety formula	C66 H78 N4 P2 Se2 Sn2, C5 H5 N, C2 H3 N	C66 H78 N4 P2 Se2 Sn2, C2 H3 N, C5 H5 N
Sum formula	C73 H86 N6 P2 Se2 Sn2	C73 H86 N6 P2 Se2 Sn2
Mr	1504.76	1504.82
Dx,g cm-3	1.433	1.433
Z	4	4
Mu (mm-1)	1.851	1.851
F000	3056.0	3053.2
F000'	3051.60	
h,k,lmax	22,29,27	22,28,25
Nref	18771	15989
Tmin,Tmax	0.769,0.897	0.886,1.000
Tmin'	0.733	

Correction method= # Reported T Limits: Tmin=0.886 Tmax=1.000
AbsCorr = GAUSSIAN

Data completeness= 0.852 Theta(max)= 29.140

R(reflections)= 0.0420(13032) wR2(reflections)= 0.1044(15989)

S = 1.046 Npar= 846

The following ALERTS were generated. Each ALERT has the format
test-name_ALERT_alert-type_alert-level.
Click on the hyperlinks for more details of the test.

Alert level C

```
PLAT042_ALERT_1_C Calc. and Reported MoietyFormula Strings  Differ     Please Check
PLAT068_ALERT_1_C Reported F000 Differs from Calcd (or Missing)...     Please Check
PLAT094_ALERT_2_C Ratio of Maximum / Minimum Residual Density ....       2.82 Report
PLAT220_ALERT_2_C NonSolvent   Resd 1  C   Ueq(max)/Ueq(min) Range        3.3 Ratio
PLAT250_ALERT_2_C Large U3/U1 Ratio for Average U(i,j) Tensor ....        2.5 Note
PLAT350_ALERT_3_C Short  C-H (X0.96,N1.08A)  C65      - H65A     .       0.84 Ang.
PLAT906_ALERT_3_C Large K Value in the Analysis of Variance ......      2.273 Check
PLAT911_ALERT_3_C Missing FCF Refl Between Thmin & STh/L=    0.600          6 Report
PLAT971_ALERT_2_C Check Calcd Resid. Dens.  0.80Ang From P2              2.21 eA-3
PLAT971_ALERT_2_C Check Calcd Resid. Dens.  0.94Ang From Se1             2.18 eA-3
PLAT971_ALERT_2_C Check Calcd Resid. Dens.  0.76Ang From P1              2.09 eA-3
```

Alert level G

```
PLAT003_ALERT_2_G Number of Uiso or Uij Restrained non-H Atoms ...         12 Report
PLAT083_ALERT_2_G SHELXL Second Parameter in WGHT  Unusually Large      15.33 Why ?
PLAT178_ALERT_4_G The CIF-Embedded .res File Contains SIMU Records          1 Report
PLAT232_ALERT_2_G Hirshfeld Test Diff (M-X)  Sn2      --P1       .        6.2 s.u.
PLAT302_ALERT_4_G Anion/Solvent/Minor-Residue Disorder (Resd  2  )       100% Note
PLAT302_ALERT_4_G Anion/Solvent/Minor-Residue Disorder (Resd  3  )       100% Note
PLAT304_ALERT_4_G Non-Integer Number of Atoms in ..... (Resd  2  )       6.57 Check
PLAT304_ALERT_4_G Non-Integer Number of Atoms in ..... (Resd  3  )       4.43 Check
PLAT328_ALERT_4_G Possible Missing H on sp3? Phosphorus .........          P1 Check
PLAT328_ALERT_4_G Possible Missing H on sp3? Phosphorus .........          P2 Check
PLAT769_ALERT_4_G CIF Embedded explicitly supplied scattering data     Please Note
PLAT790_ALERT_4_G Centre of Gravity not Within Unit Cell: Resd.  #          2 Note
              C5 H5 N
PLAT790_ALERT_4_G Centre of Gravity not Within Unit Cell: Resd.  #          3 Note
              C5 H5 N
PLAT860_ALERT_3_G Number of Least-Squares Restraints ............         186 Note
PLAT910_ALERT_3_G Missing # of FCF Reflection(s) Below Theta(Min).          3 Note
PLAT912_ALERT_4_G Missing # of FCF Reflections Above STh/L=  0.600       2636 Note
PLAT933_ALERT_2_G Number of HKL-OMIT Records in Embedded .res File          4 Note
PLAT958_ALERT_1_G Calculated (ThMax) and Actual (FCF) Lmax Differ.          2 Units
PLAT960_ALERT_3_G Number of Intensities with I  <   - 2*sig(I) ...          3 Check
PLAT978_ALERT_2_G Number C-C Bonds with Positive Residual Density.          2 Info
PLAT982_ALERT_1_G The  P-f'=    0.1043 Deviates from  IT-value =       0.1023 Check
PLAT982_ALERT_1_G The Se-f'=   -0.0811 Deviates from  IT-value =      -0.0929 Check
PLAT982_ALERT_1_G The Sn-f'=   -0.6211 Deviates from  IT-value =      -0.6537 Check
PLAT983_ALERT_1_G The  P-f"=    0.0967 Deviates from  IT-Value =       0.0942 Check
PLAT983_ALERT_1_G The Se-f"=    2.3083 Deviates from  IT-Value =       2.2259 Check
PLAT983_ALERT_1_G The Sn-f"=    1.4223 Deviates from  IT-Value =       1.4246 Check
```

0 **ALERT level A** = Most likely a serious problem - resolve or explain
0 **ALERT level B** = A potentially serious problem, consider carefully
11 **ALERT level C** = Check. Ensure it is not caused by an omission or oversight
26 **ALERT level G** = General information/check it is not something unexpected

9 ALERT type 1 CIF construction/syntax error, inconsistent or missing data

```
11 ALERT type 2 Indicator that the structure model may be wrong or deficient
 6 ALERT type 3 Indicator that the structure quality may be low
11 ALERT type 4 Improvement, methodology, query or suggestion
 0 ALERT type 5 Informative message, check
```

It is advisable to attempt to resolve as many as possible of the alerts in all categories. Often the minor alerts point to easily fixed oversights, errors and omissions in your CIF or refinement strategy, so attention to these fine details can be worthwhile. In order to resolve some of the more serious problems it may be necessary to carry out additional measurements or structure refinements. However, the purpose of your study may justify the reported deviations and the more serious of these should normally be commented upon in the discussion or experimental section of a paper or in the "special_details" fields of the CIF. checkCIF was carefully designed to identify outliers and unusual parameters, but every test has its limitations and alerts that are not important in a particular case may appear. Conversely, the absence of alerts does not guarantee there are no aspects of the results needing attention. It is up to the individual to critically assess their own results and, if necessary, seek expert advice.

Publication of your CIF in IUCr journals

A basic structural check has been run on your CIF. These basic checks will be run on all CIFs submitted for publication in IUCr journals (*Acta Crystallographica*, *Journal of Applied Crystallography*, *Journal of Synchrotron Radiation*); however, if you intend to submit to *Acta Crystallographica Section C* or *E* or *IUCrData*, you should make sure that full publication checks are run on the final version of your CIF prior to submission.

Publication of your CIF in other journals

Please refer to the *Notes for Authors* of the relevant journal for any special instructions relating to CIF submission.

PLATON version of 10/05/2023; check.def file version of 10/05/2023

Datablock 7 - ellipsoid plot

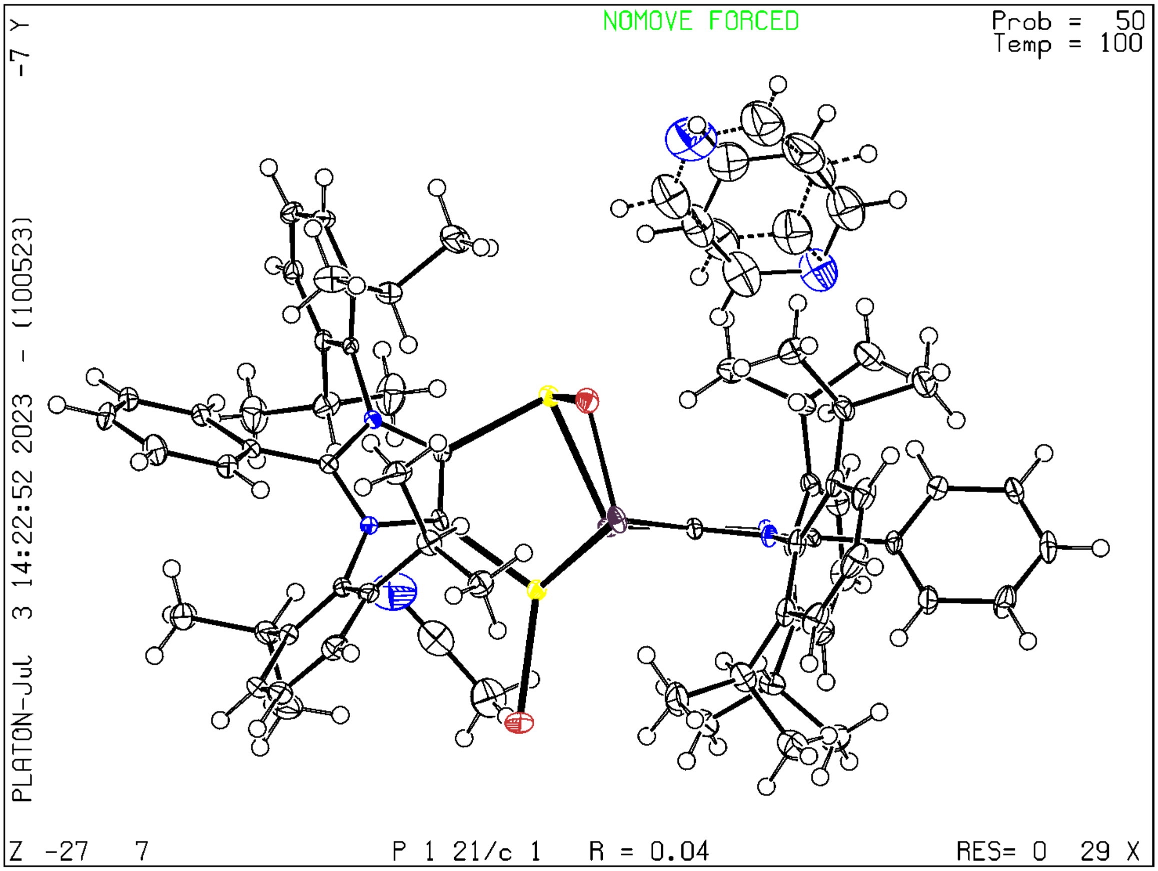

Supplement: Supplementary file 2 — Supporting Information [file ADVS-11-2305545-s002.zip › checkcif_7.pdf]
