# Supplementary material for: Isolation of an Anionic Dicarbene Embedded Sn2P2 Cluster and Reversible CO2 Uptake
Source: Adv Sci (Weinh). 2023 Nov 28;11(5):2305545. doi: 10.1002/advs.202305545 (PMC10837339; doi:10.1002/advs.202305545)

checkCIF/PLATON report

Structure factors have been supplied for datablock(s) 8

THIS REPORT IS FOR GUIDANCE ONLY. IF USED AS PART OF A REVIEW PROCEDURE FOR PUBLICATION, IT SHOULD NOT REPLACE THE EXPERTISE OF AN EXPERIENCED CRYSTALLOGRAPHIC REFEREE.

No syntax errors found. CIF dictionary Interpreting this report

Datablock: 8

Bond precision: C-C = 0.0043 A Wavelength=1.54184

Cell:	a=26.0390(2)	b=15.84659(14)	c=20.44725(16)
	alpha=90	beta=91.6449(7)	gamma=90
Temperature:	100 K		

	Calculated	Reported
Volume	8433.66(12)	8433.64(12)
Space group	P 21/c	P 1 21/c 1
Hall group	-P 2ybc	-P 2ybc
Moiety formula	C74 H78 Fe2 N4 O8 P2 Sn2, 2(C4 H8 O) [+ solvent]	C74 H78 Fe2 N4 O8 P2 Sn2, 2(C4 H8 O), 1[C4H8O]
Sum formula	C82 H94 Fe2 N4 O10 P2 Sn2 [+ solvent]	C86 H102 Fe2 N4 O11 P2 Sn2
Mr	1706.68	1778.73
Dx,g cm-3	1.344	1.401
Z	4	4
Mu (mm-1)	8.164	8.195
F000	3504.0	3664.0
F000'	3506.25	
h,k,lmax	32,19,25	32,19,25
Nref	17702	17630
Tmin,Tmax	0.320,0.486	0.380,0.614
Tmin'	0.242	

Correction method= # Reported T Limits: Tmin=0.380 Tmax=0.614
AbsCorr = GAUSSIAN

Data completeness= 0.996 Theta(max)= 76.436

```
R(reflections)= 0.0337( 15880)                         wR2(reflections)=
                                                       0.0769( 17630)
S = 0.979                    Npar= 963
```

The following ALERTS were generated. Each ALERT has the format
test-name_ALERT_alert-type_alert-level.
Click on the hyperlinks for more details of the test.

Alert level C

```
PLAT220_ALERT_2_C NonSolvent   Resd 1  C   Ueq(max)/Ueq(min) Range        3.8 Ratio
PLAT250_ALERT_2_C Large U3/U1 Ratio for Average U(i,j) Tensor ....        2.1 Note
PLAT413_ALERT_2_C Short Inter XH3 .. XHn      H45      ..H65A     .      2.11 Ang.
                                               x,3/2-y,1/2+z  =      4_576 Check
PLAT911_ALERT_3_C Missing FCF Refl Between Thmin & STh/L=    0.600         16 Report
```

Alert level G

```
FORMU01_ALERT_2_G  There is a discrepancy between the atom counts in the
            _chemical_formula_sum and the formula from the _atom_site* data.
            Atom count from _chemical_formula_sum:C86 H102 Fe2 N4 O11 P2 Sn2
            Atom count from the _atom_site data:  C82 H94 Fe2 N4 O10 P2 Sn2
CELLZ01_ALERT_1_G Difference between formula and atom_site contents detected.
CELLZ01_ALERT_1_G ALERT: Large difference may be due to a
            symmetry error - see SYMMG tests
           From the CIF: _cell_formula_units_Z    4
           From the CIF: _chemical_formula_sum  C86 H102 Fe2 N4 O11 P2 Sn2
           TEST: Compare cell contents of formula and atom_site data

           atom    Z*formula  cif sites diff
           C        344.00    328.00   16.00
           H        408.00    376.00   32.00
           Fe         8.00      8.00    0.00
           N         16.00     16.00    0.00
           O         44.00     40.00    4.00
           P          8.00      8.00    0.00
           Sn         8.00      8.00    0.00
PLAT002_ALERT_2_G Number of Distance or Angle Restraints on AtSite          4 Note
PLAT003_ALERT_2_G Number of Uiso or Uij Restrained non-H Atoms ...          6 Report
PLAT041_ALERT_1_G Calc. and Reported SumFormula    Strings  Differ     Please Check
PLAT051_ALERT_1_G Mu(calc) and Mu(CIF) Ratio Differs from 1.0 by .       0.38 %
PLAT083_ALERT_2_G SHELXL Second Parameter in WGHT  Unusually Large      21.73 Why ?
PLAT142_ALERT_4_G s.u. on b - Axis Small or Missing .............    0.00014 Ang.
PLAT143_ALERT_4_G s.u. on c - Axis Small or Missing .............    0.00016 Ang.
PLAT176_ALERT_4_G The CIF-Embedded .res File Contains SADI Records          1 Report
PLAT178_ALERT_4_G The CIF-Embedded .res File Contains SIMU Records          1 Report
PLAT188_ALERT_3_G A Non-default SIMU Restraint Value has been used     0.0100 Report
PLAT230_ALERT_2_G Hirshfeld Test Diff for    O5       --C71      .        5.5 s.u.
PLAT230_ALERT_2_G Hirshfeld Test Diff for    O7       --C73      .        7.0 s.u.
PLAT232_ALERT_2_G Hirshfeld Test Diff (M-X)  Sn1      --P2       .        9.0 s.u.
PLAT232_ALERT_2_G Hirshfeld Test Diff (M-X)  Sn2      --P1       .       11.3 s.u.
PLAT232_ALERT_2_G Hirshfeld Test Diff (M-X)  Sn2      --P2       .       10.0 s.u.
PLAT232_ALERT_2_G Hirshfeld Test Diff (M-X)  Fe1      --C67      .        5.7 s.u.
PLAT232_ALERT_2_G Hirshfeld Test Diff (M-X)  Fe1      --C69      .        6.1 s.u.
PLAT232_ALERT_2_G Hirshfeld Test Diff (M-X)  Fe1      --C70      .        5.9 s.u.
```

```
PLAT232_ALERT_2_G Hirshfeld Test Diff (M-X)  Fe2      --C71      .        8.7 s.u.
PLAT232_ALERT_2_G Hirshfeld Test Diff (M-X)  Fe2      --C72      .        5.4 s.u.
PLAT232_ALERT_2_G Hirshfeld Test Diff (M-X)  Fe2      --C73      .        9.3 s.u.
PLAT232_ALERT_2_G Hirshfeld Test Diff (M-X)  Fe2      --C74      .        6.5 s.u.
PLAT302_ALERT_4_G Anion/Solvent/Minor-Residue Disorder (Resd  2  )        60% Note
PLAT328_ALERT_4_G Possible Missing H on sp3? Phosphorus ..........         P1 Check
PLAT328_ALERT_4_G Possible Missing H on sp3? Phosphorus ..........         P2 Check
PLAT398_ALERT_2_G Deviating  C-O-C   Angle From 120 for O9       .      109.2 Degree
PLAT605_ALERT_4_G Largest Solvent Accessible VOID in the Structure        162 A**3
PLAT720_ALERT_4_G Number of Unusual/Non-Standard Labels ..........          3 Note
PLAT860_ALERT_3_G Number of Least-Squares Restraints .............         75 Note
PLAT868_ALERT_4_G ALERTS Due to the Use of _smtbx_masks Suppressed          ! Info
PLAT910_ALERT_3_G Missing # of FCF Reflection(s) Below Theta(Min).          1 Note
PLAT912_ALERT_4_G Missing # of FCF Reflections Above STh/L=  0.600         54 Note
PLAT933_ALERT_2_G Number of HKL-OMIT Records in Embedded .res File         16 Note
PLAT978_ALERT_2_G Number C-C Bonds with Positive Residual Density.          0 Info
```

---

0 **ALERT level A** = Most likely a serious problem - resolve or explain
0 **ALERT level B** = A potentially serious problem, consider carefully
4 **ALERT level C** = Check. Ensure it is not caused by an omission or oversight
37 **ALERT level G** = General information/check it is not something unexpected

4 ALERT type 1 CIF construction/syntax error, inconsistent or missing data
22 ALERT type 2 Indicator that the structure model may be wrong or deficient
4 ALERT type 3 Indicator that the structure quality may be low
11 ALERT type 4 Improvement, methodology, query or suggestion
0 ALERT type 5 Informative message, check

It is advisable to attempt to resolve as many as possible of the alerts in all categories. Often the minor alerts point to easily fixed oversights, errors and omissions in your CIF or refinement strategy, so attention to these fine details can be worthwhile. In order to resolve some of the more serious problems it may be necessary to carry out additional measurements or structure refinements. However, the purpose of your study may justify the reported deviations and the more serious of these should normally be commented upon in the discussion or experimental section of a paper or in the "special_details" fields of the CIF. checkCIF was carefully designed to identify outliers and unusual parameters, but every test has its limitations and alerts that are not important in a particular case may appear. Conversely, the absence of alerts does not guarantee there are no aspects of the results needing attention. It is up to the individual to critically assess their own results and, if necessary, seek expert advice.

Publication of your CIF in IUCr journals

A basic structural check has been run on your CIF. These basic checks will be run on all CIFs submitted for publication in IUCr journals (*Acta Crystallographica*, *Journal of Applied Crystallography*, *Journal of Synchrotron Radiation*); however, if you intend to submit to *Acta Crystallographica Section C* or *E* or *IUCrData*, you should make sure that full publication checks are run on the final version of your CIF prior to submission.

Publication of your CIF in other journals

Please refer to the *Notes for Authors* of the relevant journal for any special instructions relating to CIF submission.

PLATON version of 10/05/2023; check.def file version of 10/05/2023

Datablock 8 - ellipsoid plot

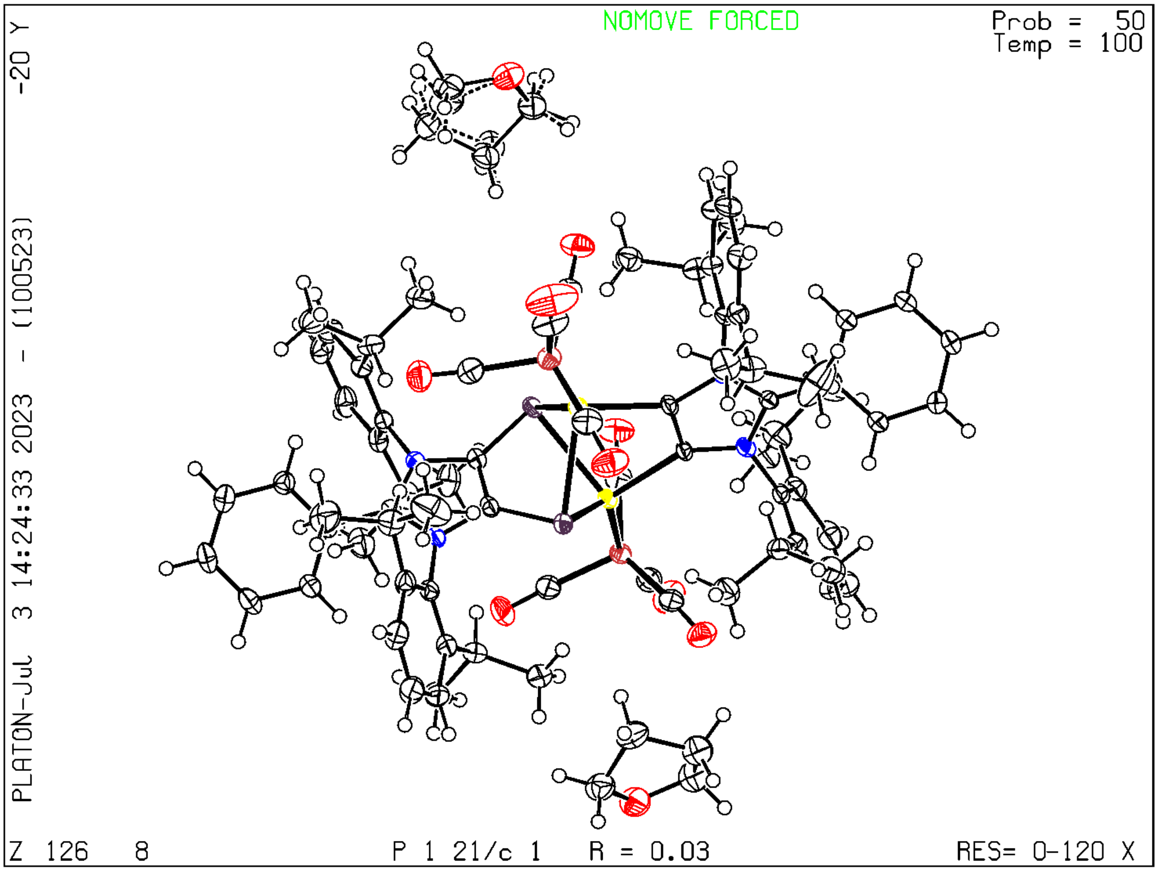

Supplement: Supplementary file 2 — Supporting Information [file ADVS-11-2305545-s002.zip › checkcif_8.pdf]
